# High levels of sewage contamination released from urban areas after storm events: A quantitative survey with sewage specific bacterial indicators

**DOI:** 10.1371/journal.pmed.1002614

**Published:** 2018-07-24

**Authors:** Hayley T. Olds, Steven R. Corsi, Deborah K. Dila, Katherine M. Halmo, Melinda J. Bootsma, Sandra L. McLellan

**Affiliations:** 1 School of Freshwater Sciences, UW-Milwaukee, Milwaukee, Wisconsin, United States of America; 2 United States Geological Survey, Upper Midwest Water Science Center, Middleton, Wisconsin, United States of America; Africa Program, UNITED STATES

## Abstract

**Background:**

Past studies have demonstrated an association between waterborne disease and heavy precipitation, and climate change is predicted to increase the frequency of these types of intense storm events in some parts of the United States. In this study, we examined the linkage between rainfall and sewage contamination of urban waterways and quantified the amount of sewage released from a major urban area under different hydrologic conditions to identify conditions that increase human risk of exposure to sewage.

**Methods and findings:**

Rain events and low-flow periods were intensively sampled to quantify loads of sewage based on two genetic markers for human-associated indicator bacteria (human *Bacteroides* and Lachnospiraceae). Samples were collected at a Lake Michigan estuary and at three river locations immediately upstream. Concentrations of indicators were analyzed using quantitative polymerase chain reaction (qPCR), and loads were calculated from streamflow data collected at each location. Human-associated indicators were found during periods of low flow, and loads increased one to two orders of magnitude during rain events from stormwater discharges contaminated with sewage. Combined sewer overflow (CSO) events increased concentrations and loads of human-associated indicators an order of magnitude greater than heavy rainfall events without CSO influence. Human-associated indicator yields (load per km^2^ of land per day) were related to the degree of urbanization in each watershed. Contamination in surface waters were at levels above the acceptable risk for recreational use. Further, evidence of sewage exfiltration from pipes threatens drinking water distribution systems and source water. While this study clearly demonstrates widespread sewage contamination released from urban areas, a limitation of this study is understanding human exposure and illness rates, which are dependent on multiple factors, and gaps in our knowledge of the ultimate health outcomes.

**Conclusions:**

With the prediction of more intense rain events in certain regions due to climate change, sewer overflows and contamination from failing sewer infrastructure may increase, resulting in increases in waterborne pathogen burdens in waterways. These findings quantify hazards in exposure pathways from rain events and illustrate the additional stress that climate change may have on urban water systems. This information could be used to prioritize efforts to invest in failing sewer infrastructure and create appropriate goals to address the health concerns posed by sewage contamination from urban areas.

## Abstract 2

Sandra McLellan and colleagues reveal the extent of sewage contamination of urban waterways following storm events. Climate change will likely see an increase in storm events and waterborne pathogens in waterways

## Author summary

**Why was this study done?:**

- Waterborne illness has been linked to extreme rainfall, and climate change is expected to bring about more intense storms in some parts of the US.
- Fecal pollution is widespread in the environment after rainfall, but high-risk sources such as sewage are difficult to distinguish from animal sources without specialized testing.
- Most water assessments are based on measuring concentrations of general indicators in grab samples; here, we set out to quantify the total amount of sewage released from a city after rainfall using automated, high-frequency sampling over multiple days.
- Understanding sewage contamination in response to rainfall dynamics and loads is important for understanding risk and how this might change with climate change.

**What did the researchers do and find?:**

- We found widespread evidence of sewage contamination following rainfall at levels high enough to exceed acceptable risk (0.03) for illness if exposed to during recreation in rivers or swimming at nearby beaches.
- Rainfall that exceeded 2 inches (50 mm) in 24 hours in spring was associated with much larger amounts of sewage contamination in the rivers and estuary compared with other rain events.
- Under conditions of very heavy rain, combined sewer overflows occurred and released 10 times more sewage compared with when there were heavy rain events with no sewage overflows.
- Sewage contamination levels were directly linked with the amount of urbanization and density of impervious surfaces in watersheds.

**What do these findings mean?:**

- Human risk could be reduced by discouraging contact with river water or nearby beaches after rainfall.
- Leaking sewage from sanitary sewer pipes can threaten surface waters and drinking water distribution pipes when they are under low pressure, such as during water main breaks.
- Quantification of sewage releases on a city-wide scale allows cities to evaluate the integrity of their sanitation systems and gauge serious infrastructure problems under increasing pressures from climate change.

## Introduction

Waterborne illness is predicted to increase as climate change alters rainfall patterns [1–4]. In particular, an increased frequency in extreme rain events is predicted for the Northeast, Pacific Northwest, and Great Lakes regions, which can increase exposure to pathogens [5]. Heavy rainfall has been linked with increased waterborne disease outbreaks [4,6]. Most notably, the waterborne outbreaks of *Escherichia coli* 0157:H7 and *Campylobacter jejuni* in Walkerton, Ontario [7,8] and *Cryptosporidium* in Milwaukee, Wisconsin [9] were preceded by extreme rainfall events, although these outbreaks also involved failures in drinking water treatment, monitoring, and human error [5,7,9]. The most common waterborne disease is gastrointestinal (GI) illness, and endemic occurrence in the community is difficult to quantify because most waterborne cases are sporadic and often not recognized as associated with water exposures [10]. However, studies estimate there are 11 to 19 million cases of GI illness from contaminated drinking water [11–13] and an estimated 90 million cases from exposure to recreational waters [14] each year.

Waterborne pathogens are carried in fecal pollution from animals and humans [10]. Humans can be infected following exposure (often through ingestion) to contaminated drinking or recreational water. Fecal pollution has been found to be widespread in the environment following rainfall events and/or snowmelt [15,16]; however, the source of contamination is difficult to discern using standard fecal bacteria indicators. Human fecal contamination, i.e., untreated sewage, has the highest potential to cause disease because humans are the reservoirs for many human pathogens [17], although agricultural runoff can also carry zoonotic pathogens [10]. In urban areas, untreated sewage released from failing sewer infrastructure can leach into soil and migrate into groundwater [18,19] and into drinking water distribution systems under conditions of reduced pressure [20]. Stormwater systems have been found to be frequently contaminated by sanitary sewage as a result of infiltration of leaking sewage or illicit cross-connections, resulting in untreated sewage discharging directly into rivers and streams [21,22]. Furthermore, under extreme precipitation events, sewer systems can become inundated with rainwater and cause sewer overflows [23,24]. Combined sewer systems are particularly vulnerable to overflows, as they collect runoff from impervious surfaces and convey sanitary sewage and stormwater to wastewater treatment plants. The US Environmental Protection Agency (EPA) estimates 850 billion gallons of untreated sewage is discharged annually into US waterways by combined sewer overflows (CSOs) and up to 10 billion gallons from separated sewer overflows (SSOs) [25]. Leaking septic systems may also contaminate groundwater or surface waters in suburban and rural areas [26,27].

*E*. *coli*, enterococci, and fecal coliforms are all commonly used as indicators of fecal pollution because they are present in the GI tract of humans and most warm-blooded animals and are easily grown in a laboratory [28]. These standard indicators, however, are not specific to the source of fecal contamination, which is important information needed to more accurately estimate risk to human health and assess sources of contamination [29,30]. Genetic markers for human-associated indicator bacteria, such as human *Bacteroides* (HB) and human Lachnospiraceae (Lachno2), can be used as proxies for human sewage. These indicators are highly correlated in sewage; thus, using them in tandem increases the reliability of tracking sewage in an urban environment where nonhuman fecal sources are also present [31,32].

Our study site in Milwaukee, Wisconsin is typical of highly urbanized areas, and by using human-associated indicators, our lab has documented frequent sewage contamination in rivers and nearshore Lake Michigan [16,31,32]. In this study, we aimed to assess the amount of sewage released from an urban area following rain events and evaluate which watershed, each with different land-use characteristics, was the largest contributor to sewage contamination. The oldest parts of the city have a combined sewer system, which are common in cities in the Northeast, Pacific Northwest, and Great Lakes regions [33]. In Milwaukee, this system overflows 1–3 times per year under conditions of extreme rainfall. We used automated, high-frequency sampling over several days to (1) quantify sewage loads discharged into Lake Michigan through the Milwaukee estuary and the three rivers upstream during low-flow periods and rain events; (2) compare the concentrations and loads produced during rain events to CSO events in relation to potential health risk; (3) investigate relationships between the degree of urbanization in watersheds and the fluxes of sewage they are contributing; and (4) establish quantitative benchmarks of sewer infrastructure integrity that can be used to monitor improvement or further deterioration.

## Methods

### Study sites and sampling methods

This study was conducted in metropolitan Milwaukee, Wisconsin, at the Milwaukee estuary and at the lower reaches of the three major rivers forming the estuary—the Milwaukee (MKE), Menomonee (MN), and Kinnickinnic (KK) Rivers. The MKE River drains the largest area and has mainly rural and agricultural land uses in the headwaters and a dense urban area near the mouth. The MN River drains a much smaller area with mainly urban and residential land uses. The KK River drains the smallest area, with nearly all urban and industrial land uses and over half of the watershed covered by impervious surfaces. For more details about the sampling locations, see **S1 Text** and **S1 Table**. The sampling and data analysis plan is provided as **S2 Text**.

**Fig 1:**
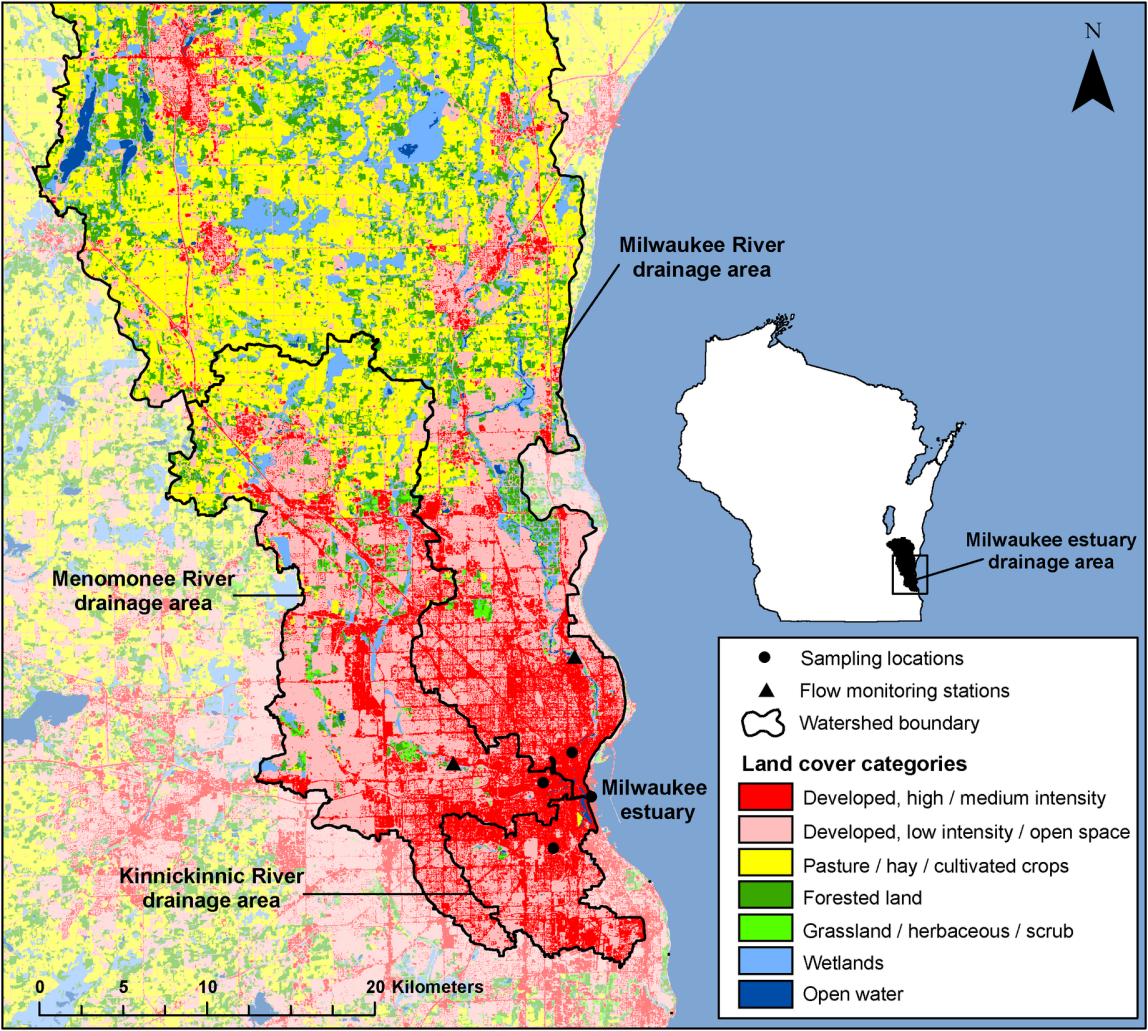
Sampling site and flow monitoring station locations, drainage areas, and land use in Milwaukee, Wisconsin. Flow monitoring stations in the KK River and Milwaukee estuary were at the sampling locations. Map inset represents the entire Milwaukee estuary drainage area in relation to the state of Wisconsin. Base data comes from the National Land Cover Database (2011) Land Cover dataset [34]. The map was generated using GIS. GIS, geographic information system; KK, Kinnickinnic.

Sampling was conducted at four sites—one in each of the three rivers and one in the Milwaukee estuary. In April through September 2014 and 2015, samples were collected across the hydrograph using automated Teledyne ISCO 3700 full-size, portable, sequential samplers housed within US Geological Survey (USGS) and Milwaukee Metropolitan Sewerage District (MMSD) monitoring stations (**Fig 1**, **S2 Table**). Samples were collected during storm events with a variety of characteristics, and routine samples were collected during periods of dry weather three to four times per sampling season. Over 2,000 samples were collected during a variety of hydrologic events. During sampling periods, a 250-mL sample was collected by the automated sampler every 15 minutes into 1-L bottles and composited in the field. For rain- and CSO-event sampling, the samplers were ideally activated a minimum of two hours prior to expected rainfall and samples were collected continuously for at least 24 hours following the rainfall event. Two 1-L sample bottles were composited in the field, resulting in two-hour composite samples with eight subsamples, for rain and CSO sampling. For dry-weather sampling, the samplers were activated after at least 48 hours of dry weather. Four 1-L bottles were composited in the field, resulting in four-hour composite samples with 16 subsamples, for dry-weather sampling. Samples were retrieved daily and processed in the laboratory within six hours of collection. Sample bottles were cleaned in the field by vigorously rinsing three times with deionized water. One field bottle blank was collected per sampling event to verify that no significant contamination was caused by residual bacteria. Field bottle blanks were collected by cleaning the sample bottles according to standard procedure, pouring deionized water into the sample bottle, and transporting and processing the blank along with the environmental samples. The full method can be found at dx.doi.org/10.17504/protocols.io.prrdm56. All field blanks were nondetections, except for one blank collected during a CSO event, which had HB and Lachno2 concentrations of 550 and 699 copy numbers (CN)/100 mL, respectively. The field procedure for rinsing bottles three times before resetting the sampler after a 24-hour sampling may not have been adequate for the high concentrations in samples during a CSO. However, this one instance of residual contamination would not affect calculations, as it is a very small fraction (<0.2%) of the concentrations detected in the samples.

### Culture-based methods, DNA extraction, and quantitative polymerase chain reaction assays

All samples collected by the automated samplers were analyzed by culture for *E*. *coli*, enterococci, and total fecal coliforms using standard methods [35–37]. A volume of 200 mL or 400 mL, depending on expected concentrations, of each sample was filtered onto a 0.22-μm-pore–sized mixed cellulose esters filter (47-mm diameter; Millipore, Billerica, MA) and stored at −80°C prior to conducting DNA extraction using the MPBIO FastDNA SPIN Kit for Soil (MP Biomedicals, Santa Anna, CA). Quantitative polymerase chain reaction (qPCR) was conducted using an Applied Biosystems StepOne Plus Real-Time PCR System Thermal Cycling Block (Applied Biosystems; Foster City, CA) with Taqman hydrolysis probe chemistry. Samples were analyzed by qPCR for the HB, Lachno2, and ruminant-specific assays using previously published methods [32,38]. Only samples from the MKE River and Milwaukee estuary were analyzed for the ruminant-specific indicator, because these sites were expected to have upstream agricultural influences. For additional details about qPCR analysis, assay slope, intercept, efficiency, and limit of quantification, see **S3 Table**. Overall, due to time and financial constraints, a representative subset of 11 rain events that spanned a range of rainfall conditions, four low-flow periods, and two CSO events were analyzed by qPCR. In total, 755 of the 2,048 samples were analyzed by qPCR for human- and ruminant-specific indicators.

### Statistical analysis

For statistical analysis, results that had detectable, but not quantifiable, concentrations below the limit of quantification were assigned a value equal to the limit of quantification (225 CN/100 mL for samples in which 200 mL were filtered; 112.5 CN/100 mL for samples in which 400 mL were filtered). Results that were nondetections (<15 CN/100 mL for samples in which 200 mL were filtered; <7.5 for samples in which 400 mL were filtered) were assigned a value of zero CN/100 mL. The Spearman’s rank correlation (rho) was used to determine correlations between quantities of genetic markers for human-associated indicator bacteria and streamflow, rainfall, or standard fecal indicators. Correlations between the two human markers were assessed using Pearson’s correlation on log10-transformed data. Differences in the ratio of these markers binned by spring/summer-fall or rain/low flow were assessed by the Wilcoxon rank–sum test. All tests were considered significant at *p* ≤ 0.05. The R suite of packages [39] was used for all statistical analyses. The stats package in R was used for Spearman’s rank correlations, Pearson’s correlations, and Wilcoxon rank–sum tests.

### Calculating maximum 24-hour mean concentrations, loads, and fluxes

Hydrologic and CSO events were defined by visually inspecting the MKE, MN, and KK River hydrographs to identify the urban runoff portion of each event. The beginning of each event was defined as the beginning of the rising limb of the hydrograph. The end of each event was designated as the approximate inflection point of the falling limb of the hydrograph, which was defined as the point where the falling limb begins to change concavity, indicating that most of the flow can be attributed to baseflow rather than runoff [40].

Maximum 24-hour mean concentrations were computed for each event in the MKE River. Streamflow was retrieved from USGS continuous monitoring stations on each river (**S2 Table**). Instantaneous loads of HB and Lachno2 were determined by multiplying streamflow by concentration. Event loads for each genetic marker were computed by integrating the product of flow and concentration over the duration of the hydrograph [41]. Individual concentrations were multiplied by the associated flow volume to compute incremental loadings. Results that had detectable, but not quantifiable, concentrations below the limit of quantification were assigned a value equal to the limit of quantification (225 CN/100 mL for samples in which 200 mL were filtered; 112.5 CN/100 mL for samples in which 400 mL were filtered). Results that were nondetections (<15 CN/100 mL for samples in which 200 mL were filtered; <7.5 for samples in which 400 mL were filtered) were assigned a value of 15 or 7.5 CN/100 mL, depending on the volume of sample filtered. The associated flow volume was estimated by summing the volumes halfway between the samples collected before and after the current sample. For the first sample of each event, volume was summed for the time period between the first and second sample with the first sample as the centroid. For the last sample of each event, volume was summed for the time period between the last and penultimate sample with the last sample as the centroid. Incremental loads from each individual sample were summed for a final event load. Daily fluxes were calculated by dividing the event load by the duration of the event in days. This allowed for a comparison between low-flow periods and storm events that were sampled over different time periods.

### Rainfall accumulation data retrieval

Watershed summaries for each sampling location were performed in a geographic information system (GIS). Watershed boundaries were defined for each site: upstream portions of basins were composed of existing linework from the Southeastern Wisconsin Regional Planning Commission [42], and downstream portions were composed of linework that was manually delineated while referencing seamless, online USGS topographic maps available through ESRI. The lower MKE River watershed was used to represent the urban-influenced area of the Milwaukee estuary and MKE River watersheds. Average one-hour rainfall accumulation for each watershed-defined area was determined using radar-indicated rainfall models retrieved from the National Weather Service North-Central River Forecast Center [43] or using MMSD rain gage data (see **S1 Text** for more information).

### Calculation of untreated sewage equivalents

The geometric mean concentrations of HB and Lachno2 markers, previously determined in 98 untreated sewage influent samples from Jones Island and South Shore wastewater treatment plants in Milwaukee, Wisconsin collected from 2009 to 2011 [44], were used to estimate the equivalent amount of untreated sewage released from the Milwaukee area following rain events. The Lachno2 marker displayed lower variability in untreated sewage and had a geomean concentration of 5.94 × 10^7^ CN/100 mL, which equates to 2.25 × 10^9^ CN/gallon. Comparisons were expressed as gallons to parallel wastewater treatment plant reporting units on volumes treated and volumes of overflows.

## Results

### Sewage concentrations and loads from rainfall events in the Milwaukee estuary

Concentrations and event loads of genetic markers for human-associated fecal indicator bacteria in the Milwaukee estuary displayed seasonal patterns, as well as relationships with rainfall and river streamflow to the estuary. In 2014 and 2015, events were sampled from early spring to late summer, with total rainfall amounts ranging from 7.4 mm during an event on August 21 and 22, 2014, to 86.9 mm during a CSO event on June 17, 2014. Events sampled in the spring of each year generally had greater total rainfall depths and mean event streamflow, with higher genetic marker concentrations measured (**Table 1**). Maximum 24-hour mean concentrations of HB and Lachno2 were up to 15 and 6 times greater during CSOs compared to the largest rain event, respectively.

**Table 1:** Peak instantaneous concentrations and maximum 24-hour mean concentrations of human *Bacteroides* (HB) and human Lachnospiraceae (Lachno2), total rainfall, and mean streamflow of storm events sampled in the Milwaukee estuary in Milwaukee, Wisconsin in 2014 and 2015.

				Peak instantaneous concentration (CN/100 mL)		Maximum 24-hour mean concentration (CN/100 mL)	
Event number	Dates	Total rainfall depth (mm)	Mean event streamflow (m^3^/s)	HB	Lachno2	HB	Lachno2
1	4/13/2014–4/15/2014	58.3	167	56,000	270,000	39,000	190,000
2	4/28/2014–4/30/2014	32.9	53	11,000	79,000	7,100	30,000
3	5/12/2014–5/14/2014	56.7	98	19,000	50,000	11,000	31,000
4	6/11/2014–6/12/2014	22.6	32	4,700	4,800	2,800	3,300
5	8/18/2014–8/20/2014	28.9	43	4,500	4,200	2,700	2,500
6	8/21/2014–8/22/2014	7.4	29	2,200	2,000	1,400	1,200
7	9/10/2014–9/11/2014	9.2	11	1,800	1,900	1,100	1,100
8	6/11/2015–6/13/2015	33.2	40	9,900	16,000	4,700	7,800
9	6/14/2015–6/15/2015	20.4	47	2,000	3,100	1,100	1,800
10	7/6/2015–7/8/2015	32.0	18	4,400	4,300	3,200	2,900
11	9/8/2015–9/9/2015	32.8	35	4,800	4,200	2,500	2,600
	6/17/2014–6/19/2014 CSO	86.9	119	850,000	2,000,000	370,000	860,000
	4/9/2015–4/11/2015 CSO	69.6	243	1,000,000	2,000,000	570,000	1,100,000
	Low flow^a^	0.00	12	1,000	1,700	340	520

^a^ Peak instantaneous concentrations of low flow represents the peak concentration of all samples collected during low-flow periods. Maximum 24-hour mean concentrations of low-flow periods represent the mean concentrations of all samples collected during low-flow periods.

**Abbreviations:** CN, copy number; HB, human *Bacteroides*; Lachno2, human Lachnospiraceae.

**Fig 2:**
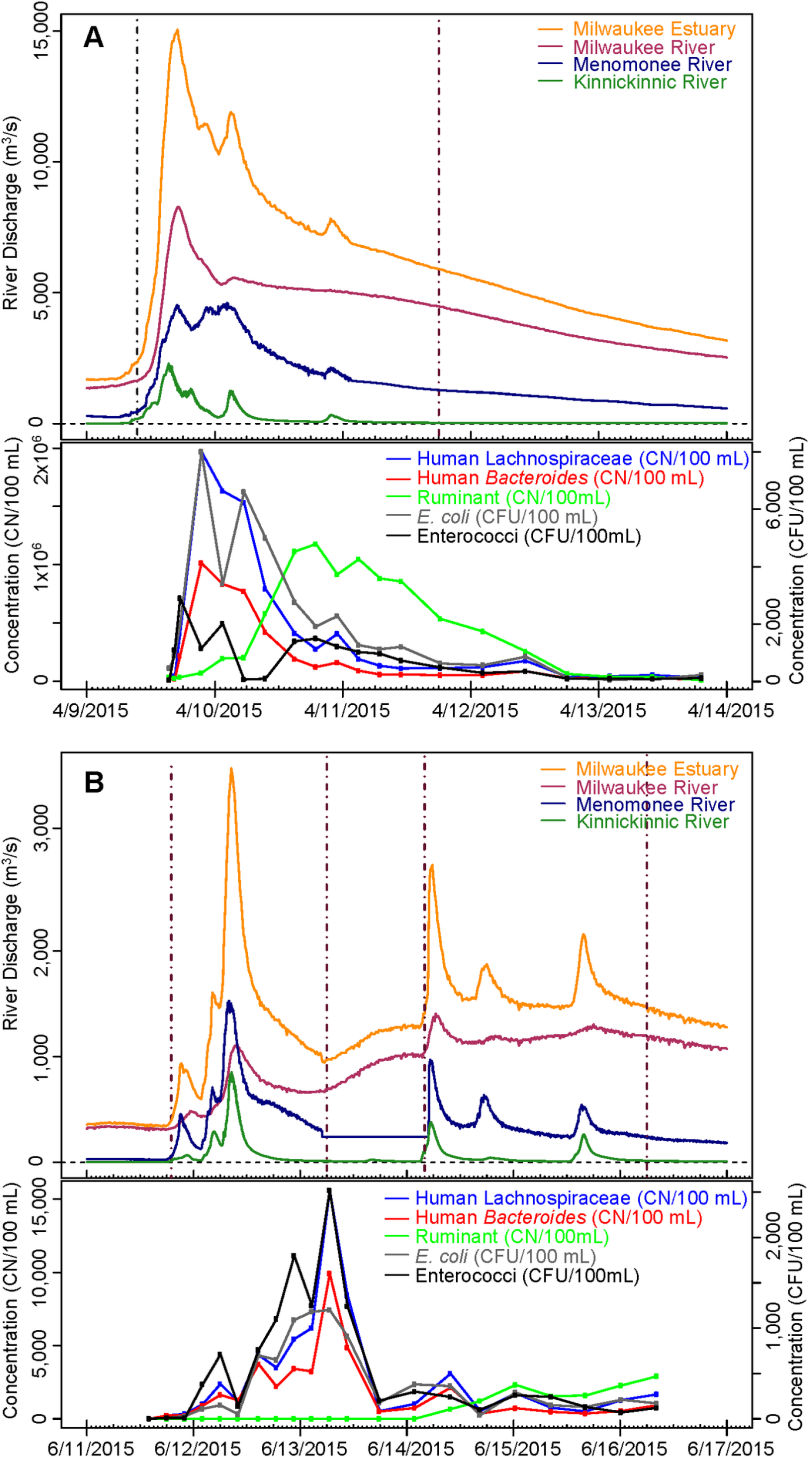
(A) Streamflow (upper panel) and corresponding HB, Lachno2, ruminant, *E*. *coli*, and enterococci indicator concentrations (lower panel) measured during a combined sewer overflow in the Milwaukee estuary in Milwaukee, Wisconsin from 4/9/2015–4/13/2015 and (B) streamflow (upper panel) and corresponding HB, Lachno2, ruminant, *E*. *coli*, and enterococci indicator concentrations (lower panel) measured during two rain events in the Milwaukee estuary in Milwaukee, Wisconsin from 6/11/2015–6/13/2015 (event 8) and 6/14 /2015–6/16/15 (event 9). Vertical black dashed lines represent the beginning and ending dates and times that were defined for each event. HB, human *Bacteroides*; Lachno2, human Lachnospiraceae.

The lowest concentrations of genetic markers were found during low-flow periods. Concentrations of genetic markers showed a consistent pattern of increased concentrations with increased flow across the hydrograph measured at the estuary (**Fig 2**, **S2 Fig**). The three rivers that collectively drain to the estuary mirrored this pattern (**S3 Fig**, **S4 Fig**, **S5 Fig**). The full set of hydrographs and corresponding host-associated indicators for all events at the estuary and three rivers are shown in **S2 Fig**, **S3 Fig**, **S4 Fig**, and **S5 Fig**. Of all samples collected in the Milwaukee estuary during a variety of weather conditions in 2014 and 2015 that were analyzed by qPCR (*n* = 188, Milwaukee estuary site only), concentrations of the two human indicators were significantly correlated to event streamflow volume (HB rho = 0.69, Lachno2 rho = 0.74, *p* < 0.05) and maximum river streamflow (HB rho = 0.71, Lachno2 rho = 0.75, *p* < 0.05) measured during sample collection.

A ruminant-specific genetic marker was also analyzed in Milwaukee estuary samples because of the rural and agricultural land uses in the headwaters of the MKE River. The ruminant signal was generally either absent or present at low levels throughout the duration of a rain event but then was detected at greater levels several days following rainfall (**Fig 2B**, **S2 Fig** and **S5 Fig**). General indicators *E*. *coli* and enterococci were frequently elevated when either human or ruminant markers increased, illustrating the lack of specificity of these indicators (**S2 Fig** and **S5 Fig**).

HB and Lachno2 concentrations were highly correlated among all Milwaukee estuary samples (*r* = 0.99; *p* < 0.05; *n* = 188), with Lachno2 concentrations on average 1.9 times higher than HB concentrations. Season and/or temperature appeared to influence the ecology of these indicators differently. The ratio between Lachno2 and HB peak instantaneous and maximum 24-hour mean concentrations were significantly higher (*p* < 0.01) in samples collected in the spring than those collected during the summer and fall. Concentrations of Lachno2 in samples collected in the spring ranged from two to five times higher than HB concentrations, whereas samples collected in the summer and during low flow had ratios of Lachno2 to HB that ranged from 1 to 1.5.

**Fig 3:**
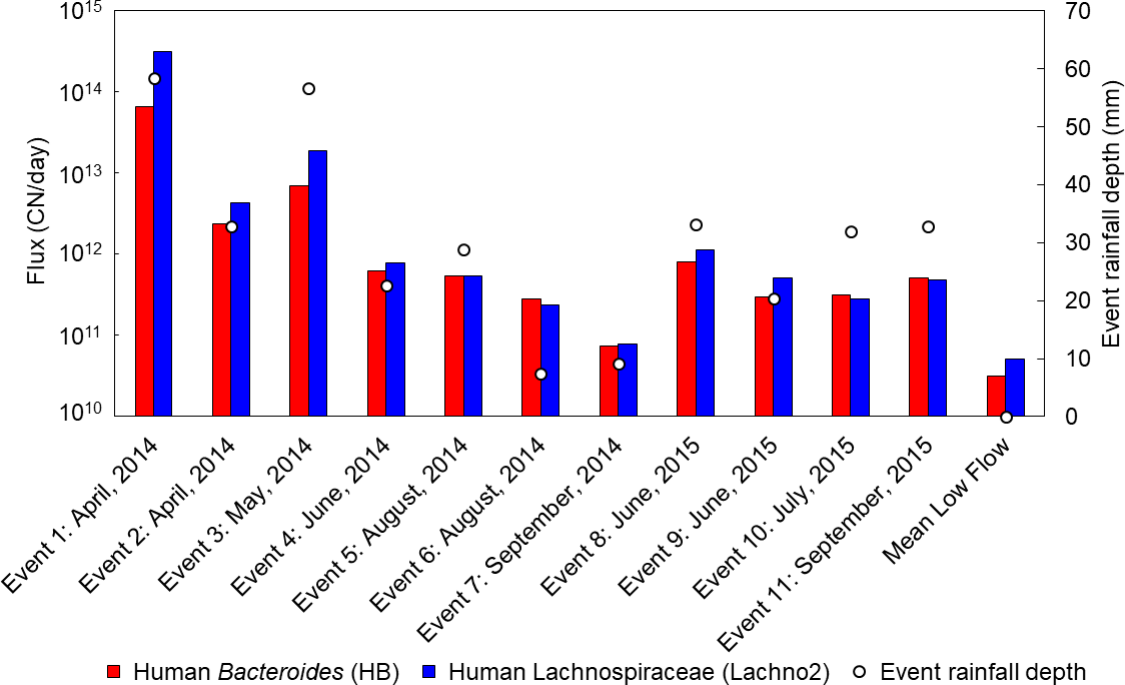
Daily fluxes of HB and Lachno2 during 11 storm events and mean daily fluxes of these human-associated indicators collected during low flow and event rainfall depth of each event and low-flow sampling period in the Milwaukee estuary in Milwaukee, Wisconsin in 2014 and 2015. HB, human *Bacteroides*; Lachno2, human Lachnospiraceae.

Daily flux of HB and Lachno2 were calculated for rain events with no CSOs and low-flow periods to examine the amount of unrecognized sewage inputs as a result of rainfall (**Fig 3**). Rain event fluxes and total rainfall depth were significantly correlated for both human indicators (HB rho = 0.90, Lachno2 rho = 0.84, *p* < 0.05). Rain event daily fluxes ranged from 7.4 × 10^10^ CN/day of HB and 7.9 × 10^10^ CN/day of Lachno2 released in the estuary during an event in September 2014 (Event 7) to 6.6 × 10^13^ CN/day of HB and 3.1 × 10^14^ CN/day of Lachno2 during a storm event in April 2014 (Event 1). This equates to a 1,000-fold difference in amount of sewage released from a very light rain (9.2 mm) in fall versus a heavy rain (58.3 mm) in spring.

### Individual watershed contributions to sewage loading in the Milwaukee estuary

**Fig 4:**
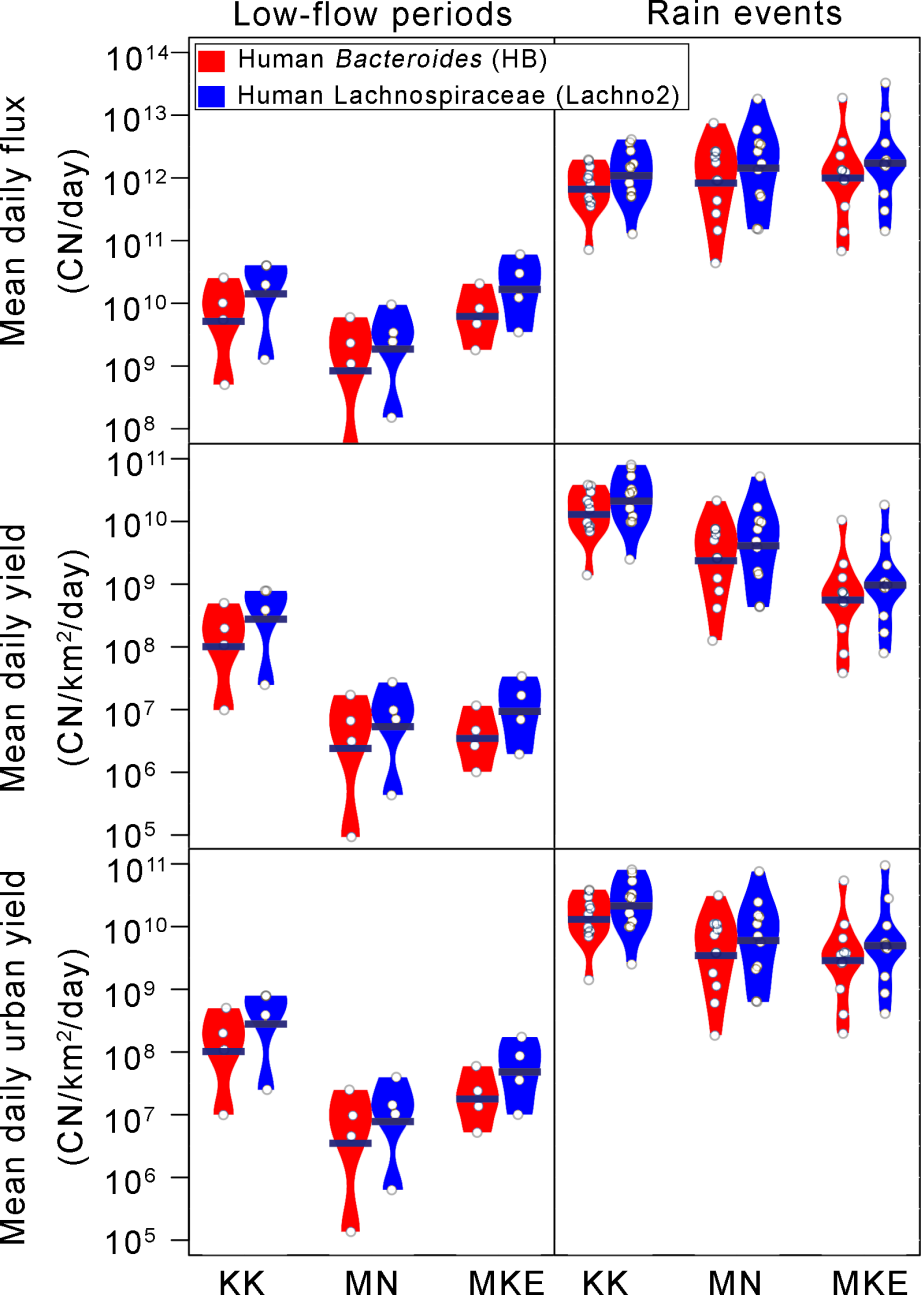
Mean daily fluxes, mean daily yields, and mean daily fluxes per unit urban area of HB and Lachno2 in the KK River, MN River, and MKE River for 11 rain events and four low-flow periods collected in Milwaukee, Wisconsin in 2014 and 2015. Y-axis is plotted on a log scale. HB, human *Bacteroides*; KK, Kinnickinnic; Lachno2, human Lachnospiraceae; MKE, Milwaukee; MN, Menomonee.

We measured sewage loading from the three rivers that discharge to the estuary to examine the association between sewage releases and the different size and land use of each watershed. In general, the MKE River, which has the highest flow and drainage area, had higher human genetic marker event loads and daily fluxes during rain events compared to the other rivers, but on average, this difference was modest (**Fig 4**).

The flux (CN per day) from each watershed following rain was driven by size, as we found that during the study period in 2014 and 2015, MKE River streamflow was on average two times greater than MN River streamflow and about five times greater than KK River streamflow, and the differences in flux of human markers from the three watersheds was proportional to this difference in flow. However, when fluxes were normalized by drainage area to calculate yield per day, mean daily yields (CN/km^2^ of entire watershed per day) of both human genetic markers in the KK River were approximately three times greater than those in the MN River and approximately 11 times greater than those in the MKE River (**Fig 4**).

We also compared sewage loading from different watersheds based on the amount of urbanization and imperviousness (increased runoff) by calculating the yield as a daily flux per urban land cover (CN/km^2^ of urban area per day). During rain events, urban yields of both genetic markers were more similar across the three watersheds, with average urban yields of genetic markers in the KK River only two times greater than the MN and MKE Rivers (**Fig 4**). Across the range of different rainfall amounts, the sewage signal from the KK River was more consistent than the MN or MKE Rivers.

During low-flow periods, mean daily fluxes of HB and Lachno2 were 30 to 40 times greater in the KK River than the MN and MKE Rivers. The KK River also has a much larger urban yield than the other two watersheds, suggesting that more sewage was released per unit urban area particularly under low-flow conditions. (**Fig 4**).

### Standard water quality indictors and relationship to sewage contamination

**Fig 5:**
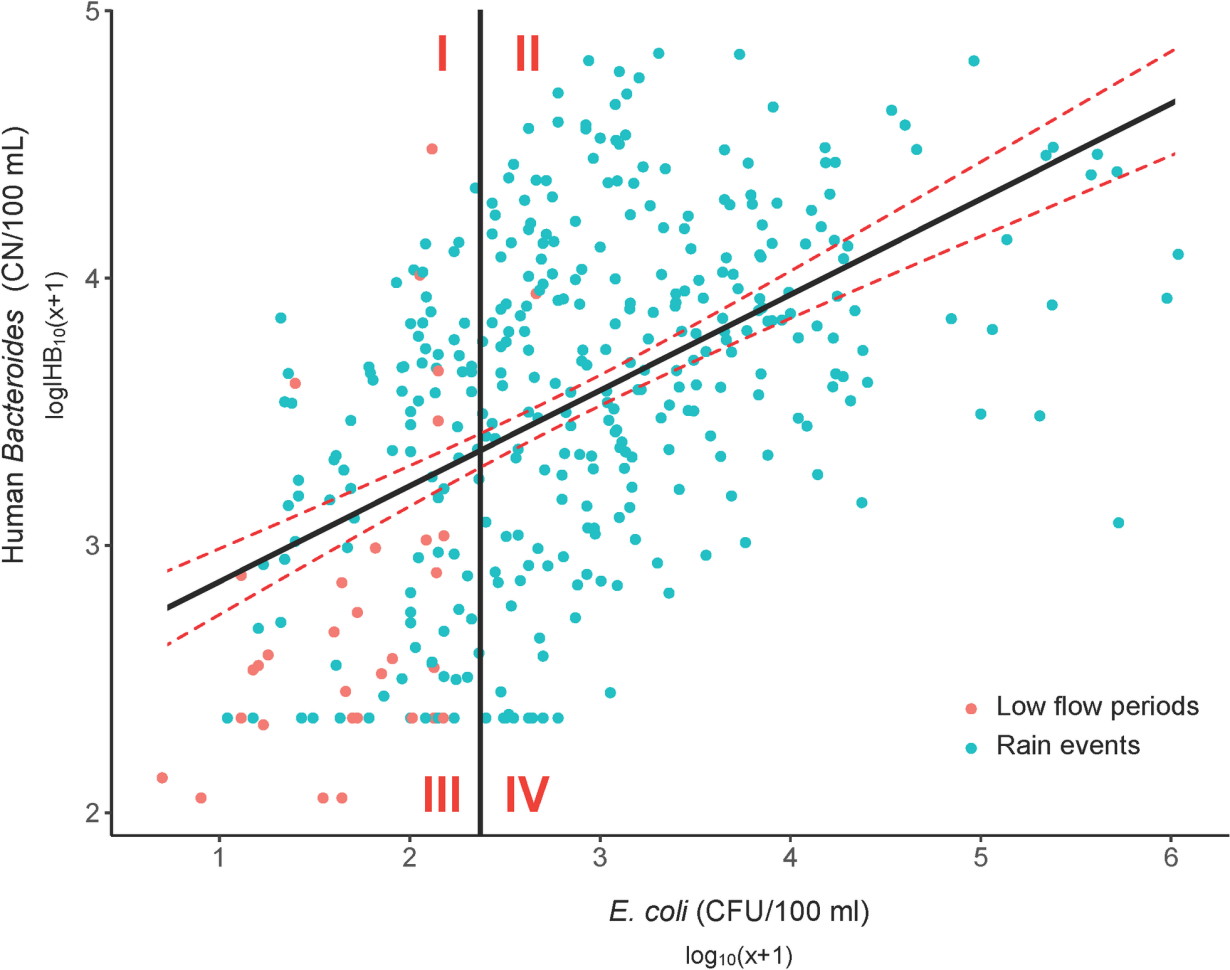
Scatter plot of log10-transformed *E*. *coli* culture results and the HB genetic marker. Quadrants were defined as *E*. *coli* above and below the water quality advisory limit of 235 CFU/100 mL, and the regression line value of HB at this limit. Interval confidence (dashed lines) have been calculated using a linear model with an r-squared of 0.27. HB, human *Bacteroides*.

The KK, MN, and MKE Rivers and the estuary consistently exceeded water quality standards for *E*. *coli*, enterococci, and fecal coliforms (**S1 Fig**), particularly under rainfall conditions. There was overall a poor relationship between general and human indicators, with a rho = 0.29 for *E*. *coli* versus HB. These weak correlations likely reflect the nonspecific nature of these general fecal indicators. We explored the distribution of HB values in respect to *E*. *coli* above or below the water quality advisory limit of 235 colony-forming units (CFU)/100 mL (**Fig 5**). Very few values fell within the 90% confidence interval, illustrating the poor overall relationship. We were primarily interested in the number of values that fell in quadrant I, where *E*. *coli* values were below 235 CFU/100 mL and were outside the 90% confidence interval of what would be predicted for HB; these samples represent the greatest concern for protecting human health because *E*. *coli* would not indicate sewage contamination was present.

### Combined sewer overflow events

Two CSO events were sampled on June 18 and 19, 2014, and April 9 and 10, 2015. Based on volumes reported by MMSD, approximately 341.2 million gallons (MG) of untreated sewage mixed with stormwater was released during the 2014 CSO, and 681.1 MG was released during the 2015 CSO. The majority of sewage released during the CSOs occurred at discharge points from the combined sewer system along the KK, MN, and MKE Rivers. The loads of HB and Lachno2 captured at the automated sampling station at each river were determined, and assuming the markers were primarily from CSOs, these loads were proportional to the volume of release in each river (**S4 Table**). This illustrates the ability of the two human-associated markers to quantify sewage releases reliably.

During CSOs, large volumes of stormwater are mixed with untreated sewage and discharged into urban waterways, making it difficult to estimate what portion of the discharged water is raw sewage and what portion is stormwater. Concentrations of genetic markers for human-associated fecal indicator bacteria in untreated sewage influent samples at the Jones Island and South Shore wastewater treatment plants were used to estimate how many gallons of untreated sewage were discharged into the rivers and the estuary. Lachno2 marker was used because it was the most consistent of the two human makers in treatment plant influent. The geometric mean concentration of Lachno2 in sewage influent samples collected by from 2009–2011 (*n* = 98) was 2.25 × 10^9^ CN/gallon (i.e., 5.94 × 10^8^ CN/L). Loads of the Lachno2 indicator were converted to gallons of “untreated sewage equivalents” in waterways (**Table 2**).

**Table 2:** Sewage equivalents calculated for two high-intensity rain events and two CSO events, as well as volumes of sewage released during each CSO in the KK, MN, and MKE Rivers in Milwaukee, Wisconsin in 2014 and 2015.

				Untreated sewage equivalents (gallons)			
Event type	Rainfall amount (mm)^a^	Rainfall intensity (mm/hr)^b^	Dates	KK River	MN River	MKE River	Milwaukee estuary^c^
Mean low flow per day	0.0	0.0	varies	11	1.7	12	23
Rain Event	58.3	0.96	4/13/2014–4/15/2014	1,400	8,700	1.6 × 10^4^	1.5 × 10^5^
Rain Event	56.7	1.16	5/12/2014–5/15/2014	550	3,600	7,800	1.5 × 10^4^
CSO	86.9	2.41	6/17/2014–6/20/2014	1,400	7.6 × 10^5^	2.1 × 10^5^	6.2 × 10^5^
CSO	69.6	2.40	4/9/2015–4/11/2015	1,200	8.5 × 10^5^	4.0 × 10^5^	1.5 × 10^6^

^a^ Rainfall amounts are reported for the Milwaukee estuary watershed as a whole.

^b^ Rainfall intensities are reported for the Milwaukee estuary watershed as a whole and are an average for the event.

^c^ Backflow of the harbor into the estuary may have reduced measured levels and therefore reduced estimates of untreated sewage equivalents in cases in which numbers are lower than the sum of the three rivers.

**Abbreviations:** CSO, combined sewer overflow; KK, Kinnickinnic; MKE, Milwaukee; MN, Menomonee.

## Discussion

To understand climate influences on waterborne disease threats, drivers of contamination and exposure pathways need to be better characterized [5,10]. A key component is to understand mechanisms of sewage contamination of waterways and identify “high-risk” rainfall conditions, such as extreme events. Moving past simply measuring concentrations of standard fecal indicator bacteria in water to using highly specific indicators indicative of human fecal sources will allow us to quantitatively assess urban infrastructure vulnerabilities and provide better estimates of potential risk due to waterborne pathogens.

### Quantification of sewage with two human-associated indicators

This study showed the utility of using two human genetic markers, HB and Lachno2, in tandem to reliably track sewage contamination. The steady-state concentrations in untreated sewage reflected the overall contribution of a human population of approximately 1 million people in the service area. Levels of human-associated fecal indicators have been related to pathogen concentrations in untreated sewage to estimate potential human health risk [44–46]. These assays, as well as other genetic marker assays that are used for microbial source tracking, are generally specific to humans but have been found to sporadically amplify animal fecal sources [47–50]; therefore, using two human-associated indicators improved reliability. In this study, HB and Lachno2 were highly correlated in river and estuary samples, indicating there is a high probability that the fecal pollution is from a human sewage source [31]. We found differences in the ratio of the two indicators in spring rain events compared with other sample times, suggesting Lachnospiraceae (a gram-positive organism) and *Bacteroides* (a gram-negative organism) have different survival characteristics in the environment. Further work to understand the variables (e.g., time and temperature) that account for different ratios would be useful to create a metric for how long sewage contamination has been in the environment, which would influence the infectivity of waterborne pathogens.

### Rainfall as a driver of human fecal pollution in urban waterways

There is accumulating evidence that sewage leaking from sanitary sewer infrastructure can be mobilized during rain events and contaminate stormwater systems [21,22]. In this study, we attempt to quantify this “pulse” of sewage from a metropolitan area under different rainfall conditions. The majority of our urbanized study area has separated sewer systems, with only 12% of the sewer service area comprised of combined sewers. We found increased concentrations of human-associated indicators with increased flow across all three watersheds and in the estuary, suggesting sewage sources were dependent on hydrologic influences. These results are consistent with other studies which have also found that concentrations of fecal indicator bacteria increase and decrease with changes in streamflow [51,52].

We observed two inches (50 mm) of rain in the spring resulted in dramatic increases in sewage contamination in the absence of reported overflows. This suggests there may be a critical threshold for municipal sanitary sewers in separated sewer systems, similar to the levels of rainfall that we found can trigger a CSO [53]. This is important because there is a predicted increase in frequency of these types of rain events for certain parts of the country [2,5]. Rainwater infiltration and inflow to separated sanitary sewer pipes can drastically increase the volumes of water in the sewer system [25]. Monitoring programs in urban areas could be designed to intensively sample during large rainfalls to determine what critical rainfall amounts overwhelm separated sanitary systems their city.

### Combined sewer overflow events and climate change

Combined sewer systems in the oldest parts of some US cities are legacy infrastructure from the early 1900s, and the EPA permits a certain number of discharges from these systems [25]. Under the largest storm events, CSOs introduce pathogens, particularly human viruses, into receiving waters [54,55]. In 2014, 187 communities released 22 billion gallons of untreated sewage mixed with stormwater into the Great Lakes [33], which are a drinking water source to nearly 40 million people and have more than 500 beaches along the 4,500 miles of coastline. The highest density of combined sewer systems are in the Northeast, Pacific Northwest, and Great Lakes regions, which are the same regions that are predicted to have the largest increase in extreme events due to climate change [2,5,56]. Although CSOs pose an obvious health risk over a few days per year, this is rivaled by the chronic health risk caused by lesser but more persistent contamination introduced after rainfall.

### Potential targets for remediation

We demonstrated that across three watersheds with varying drainage areas and land use, urbanization can primarily account for sewage yields, which suggests our results could be generalized to other urban areas in the US. We found the KK River was a consistent sewage source regardless of rainfall. The KK River is the most urbanized and downstream watershed of the three, suggesting that even low amounts of rainfall effectively mobilize sewage that has escaped the sanitary sewer pipes through failing infrastructure or illicit connections, whereas in the MN and MKE River watersheds, larger rainfalls were needed to mobilize this system. These results might suggest that failing infrastructure is more problematic in the KK watershed. Differences in transport or attenuation of contamination from this small watershed near the estuary compared with the larger MN and MKE watershed may also play a role. Numerous stormwater outfalls line the concrete channel of the KK River and likely serve as a conduit for leaking sanitary sewers and sewage from illicit cross-connections to reach the river [22,32].

### Distinguishing sewage from nonhuman sources of fecal contamination

Standard fecal indicators can exceed standards when sewage indicators are at low levels (**Fig 5**, indicated by quadrant IV). The majority of samples with these characteristics were in the MN and MKE Rivers, which suggests a portion of the water quality exceedances in these rivers may be attributable to sources other than sewage. In contrast, the KK river appears to have sewage as the major source of contamination. Possible nonhuman sources include pet waste and urban wildlife (in urban areas) and animal manure and wildlife (in rural and agricultural areas). Following rainfall, a clear signal from the ruminant genetic marker, with decreases in sewage markers, indicated that agricultural runoff is a likely source of fecal pollution in the MKE River watershed late in the event. While human sewage is considered the highest risk, agricultural runoff can also carry human pathogens [57].

### Exposure pathways and health outcomes

Epidemiology studies and evaluation of outbreaks have identified an association between rain events and GI illness, particularly in children [4,6,58,59]. Release of untreated sewage is the major pathway for introduction of waterborne pathogens into the environment, creating exposure routes through recreational and drinking water. In our study area, drinking water is drawn from Lake Michigan several kilometers from the harbor and intakes are at a depth of approximately 20 meters, so any contamination in source water is highly diluted; however, communities that draw their drinking from rivers near urban areas may have higher concentrations of sewage contamination. Furthermore, drinking water treatment is designed to remove pathogens to levels safe for consumption, but the Milwaukee *Cryptosporidium* outbreak of 1993 illustrates the consequences of failures in this protective barrier [9].

Drinking water distribution systems may be more the more likely route by which humans are exposed to pathogens from sewage. Release of untreated sewage through stormwater systems is an indicator of sewage exfiltration from failing sanitary sewer pipes [21,60]. This leaking sewage can infiltrate drinking water distribution pipes under conditions of low water pressure or when there is a water main break [61]. Sewage can also contaminate groundwater that is used as a drinking water source, which is of high concern [18,19,59,62], particularly if it is untreated [63]. Drinking water systems, along with wastewater and sewer conveyance infrastructure, are ranked as a D− and D, respectively, by the American Association of Civil Engineers [64], and can be expected to deteriorate further over time without significant investments.

Exposure to pathogens through recreational water is not trivial, which is illustrated by a study that estimates there are 90 million cases of illness related to recreational contact with contaminated water per year [14]. Urban beaches are often located near river discharge that can impact those sites, especially following sewer overflows [55,65]. Kayaking and rowing in urban rivers is also becoming more popular, but these waterways often do not have monitoring or water quality advisory systems in place. After rainfall, concentrations of the human-associated indicator HB ranging from 4,200 to 7,800 CN/100 mL have been estimated to be equivalent to a 0.03 risk of illness following typical recreational exposure [44,57,46]. Concentrations and loads during CSO events were approximately 10-fold higher than rain events with no sewage overflows, and studies have noted that a 1:30 dilution of CSO water still presents a serious health risk [54]. Delivery of diluted CSO-contaminated water to nearby beaches is of high concern since culturable indicators may be short lived and contamination may go unrecognized using standard beach monitoring methods [65].

### Challenges to understanding the links between water, climate, and health

Linking pathogen levels in the environment to human health outcomes is complex and not feasible to do within a single study framework. Pathogens are generally at low levels and intermediately present in the environment, making them difficult to quantify in exposure pathways. In this study, source-specific indicators of sewage helped fill this gap and can infer environmental concentrations of waterborne pathogens [30,44]. These indicators can be used to assess recreational waters and surface waters; however, documenting sewage intrusion into drinking water distribution systems is not practical because contamination occurs infrequently and sporadically in small segments of the system [20]. Large epidemiology studies have linked viruses in tap water with illness in communities served by untreated groundwater, but measurements were taken over a fixed 12-week period and did not focus on rainfall events [63]. Multiple studies have linked rainfall occurrence with illness, which in and of itself is challenging. Most health outcomes are based on either outbreak data [4] or hospital or clinic visits [58,59], but the major health effects are likely sporadic occurrences of GI illness, which usually go unreported [1,5]. Quantitative microbial risk assessment can bridge some of these gaps and provide insights into intermediate risk factors through determining levels of sewage contamination in exposure pathways. Better characterization of actual exposure rates and adverse outcomes needs to be linked with pathogen burdens in the environment to fully understand health outcomes due to rain events.

### Conclusions

GI illness has been shown to increase in the community following rainfall [4,6,58,59]. We demonstrated that sewage contamination, which carries many GI pathogens, is widespread in urban waterways following rainfall and 10-fold higher following CSOs. Human exposure could be reduced by limiting contact with recreational waters and following boil water advisories when they are issued due to water main breaks or other breaches in drinking water systems. Furthermore, vulnerable populations such as those that are immunocompromised should be cautious about exposure to surface waters after rainfall. Sewage contamination was related to the degree of urbanization in the watershed, illustrating the widespread nature of urban sewer infrastructure problems. Urban sewer infrastructure is currently under stress during rainfall due to deterioration of pipes and legacy combined sewer systems and may be more vulnerable in the future with changing rainfall patterns under climate change conditions. Future investments in repairing these systems and public health messages that are informative about potential exposure could reduce the endemic waterborne illness burden due to sewage contamination.

## Supporting information

S1 Text Detailed study area and sampling location descriptions; detailed methods for qPCR analysis, flow data retrieval, and rainfall data retrieval; and discussion about complications to load and mass balance computations. qPCR, quantitative polymerase chain reaction.
(PDF)

S2 Text QAPP for this study. QAPP, Quality Assurance Project Plan.
(PDF)

S1 Table Watershed characteristics of sampling sites in Milwaukee, Wisconsin. (PDF)

S2 Table USGS flow-monitoring station used to retrieve continuous river discharge data. USGS, US Geological Survey.
(PDF)

S3 Table qPCR assays slopes, Y intercepts, and efficiencies for Lachno2 and HB. HB, human *Bacteroides*; Lachno2, human Lachnospiraceae; qPCR, quantitative polymerase chain reaction.
(PDF)

S4 Table Volumes, in MG, and percentages of total volumes released from CSO outfalls upstream of automated sampling locations in the KK, MN, and MKE Rivers during CSO events in 2014 and 2015, as reported by the MMSD. Loads of HB and Lachno2 for each river and the percentages of the total load (sum of three rivers) at automated sampling locations are shown. CSO, combined sewer overflow; HB, human *Bacteroides*; KK, Kinnickinnic; Lachno2, human Lachnospiraceae; MG, million gallons; MKE, Milwaukee; MMSD, Milwaukee Metropolitan Sewerage District; MN, Menomonee.
(PDF)

S1 Data Concentrations and instantaneous loads of human-associated indicator bacteria computed for samples collected in the KK River, MN River, MKE River, and Milwaukee estuary (MKE estuary) in Milwaukee, Wisconsin in 2014 and 2015. CN, number of copies; HB, human Bacteroides; KK, Kinnickinnic; km2, square kilometers; Lachno2, human Lachnospiraceae; m3, cubic meters; MKE, Milwaukee; mL, milliliters; mm, millimeters; MN, Menomonee; NA, not-applicable or not-analyzed; UTC, Universal Time Coordinated.
(XLSX)

S2 Data Loads and fluxes computed for 11 rain event periods, four low-flow periods, and two combined sewer overflow events in the KK River, MN River, MKE River, and Milwaukee estuary (MKE estuary) in Milwaukee, Wisconsin in 2014 and 2015. CN, number of copies; HB, human Bacteroides; KK, Kinnickinnic; km2, square kilometers; Lachno2, human Lachnospiraceae; m3, cubic meters; MKE, Milwaukee; mL, milliliters; mm, millimeters; MN, Menomonee; NA, not-applicable or not-analyzed; UTC, Universal Time Coordinated.
(XLSX)

S3 Data Concentrations of standard fecal indicator bacteria for samples collected in the KK River, MN River, MKE River, and Milwaukee estuary (MKE estuary) in Milwaukee, Wisconsin in 2014 and 2015. CFU, colony-forming units; KK, Kinnickinnic; MKE, Milwaukee; mL, milliliters; MN, Menomonee; NA, not applicable or not analyzed; UTC, Universal Time Coordinated.
(XLSX)

S1 Fig Concentrations of standard fecal indicator bacteria, *E*. *coli*, enterococci, and fecal coliforms, measured in the KK, MN, and MKE Rivers, as well as the Milwaukee estuary during low-flow periods (white plots) and rain events (gray plots) in Milwaukee, Wisconsin in 2014 and 2015. Red dotted lines represent the ambient water quality standards for geometric means for *E*. *coli* (126 CFU/100 mL), enterococci (35 CFU/100 mL), and fecal coliforms (200 CFU/100 mL). CFU, colony-forming unit; KK, Kinnickinnic; MKE, Milwaukee; mL, milliliters; MN, Menomonee.
(PDF)

S2 Fig **Streamflow (upper panel) and corresponding HB, Lachno2, ruminant, *E. coli*, and enterococci indicator concentrations (lower panel) measured during rain event and low-flow periods in the Milwaukee estuary in Milwaukee, Wisconsin in 2014 and 2015**. Each letter (A–N) represents a different sampling period. Vertical black dashed lines represent the beginning and ending dates and times that were defined for each event or low-flow period. HB, human *Bacteroides*; Lachno2, human Lachnospiraceae.
(PDF)

S3 Fig **Streamflow (upper panel) and corresponding HB, Lachno2, *E*. *coli*, and enterococci indicator concentrations (lower panel) measured during rain event and low-flow periods in the KK River in Milwaukee, Wisconsin in 2014 and 2015**. Each letter (A–N) represents a different sampling period. Vertical black dashed lines represent the beginning and ending dates and times that were defined for each event or low-flow period. HB, human *Bacteroides*; KK, Kinnickinnic; Lachno2, human Lachnospiraceae.
(PDF)

S4 Fig **Streamflow (upper panel) and corresponding HB, Lachno2, *E*. *coli*, and enterococci indicator concentrations (lower panel) measured during rain event and low-flow periods in the MN River in Milwaukee, Wisconsin in 2014 and 2015**. Each letter (A–N) represents a different sampling period. Vertical black dashed lines represent the beginning and ending dates and times that were defined for each event or low-flow period. HB, human *Bacteroides*; Lachno2, human Lachnospiraceae; MN, Menomonee.
(PDF)

S5 Fig **Streamflow (upper panel) and corresponding HB, Lachno2, *E*. *coli*, and enterococci indicator concentrations (lower panel) measured during rain event and low-flow periods in the MKE River in Milwaukee, Wisconsin in 2014 and 2015**. Each letter (A–N) represents a different sampling period. Vertical black dashed lines represent the beginning and ending dates and times that were defined for each event or low-flow period. HB, human *Bacteroides*; Lachno2, human Lachnospiraceae; MKE, Milwaukee.
(PDF)

## Abbreviations

CN: copy numbers
CFU: colony-forming units
CSO: combined sewer overflow
EPA: Environmental Protection Agency
GI: gastrointestinal
GIS: geographic information system
HB: human *Bacteroides*
KK: Kinnickinnic
Lachno2: human Lachnospiraceae
MG: million gallons
MKE: Milwaukee
MMSD: Milwaukee Metropolitan Sewerage District
MN: Menomonee
qPCR: quantitative polymerase chain reaction
rho: Spearman’s rank correlation coefficient
SSO: separated sewer overflow
USGS: US Geological Survey
