# Supplementary material for: High levels of sewage contamination released from urban areas after storm events: A quantitative survey with sewage specific bacterial indicators
Source: PLoS Med. 2018 Jul 24;15(7):e1002614. doi: 10.1371/journal.pmed.1002614 (PMC6057621; doi:10.1371/journal.pmed.1002614)
Supplement: S1 Text — qPCR, quantitative polymerase chain reaction. (PDF) [file pmed.1002614.s001.pdf]

S1 Text. Detailed study area and sampling location descriptions; detailed methods for qPCR analysis, flow data retrieval, and rainfall data retrieval; and discussion about complications to load and mass balance computations. qPCR, quantitative polymerase chain reaction

Any use of trade, firm, or product names is for descriptive purposes only and does not imply endorsement by the U.S. Government.

Study area and sampling locations

S1 Table represents drainage area, land use, and impervious surface percentages for the Milwaukee (MKE), Menomonee (MN), and Kinnickinnic (KK) Rivers in Milwaukee, Wisconsin. Land use and impervious surface percentages for each watershed were determined by using the ESRI ArcGIS® software package and data from the 2011 National Land Cover Database (Jin et al., 2013; Xian et al., 2011). Urban land use was defined as any land cover classified as developed land, including low, medium, and high intensity developed land, and developed open space. This includes all low to moderately developed areas, including urban parks, golf courses, and residential areas, as well as highly developed commercial and industrial areas. The Milwaukee estuary site was at the USGS station at Jones Island water reclamation facility in Milwaukee, Wisconsin (USGS station number 04087170). The MKE River sampler was housed within an MMSD real-time water quality station beneath the Cherry Street Bridge in Milwaukee, WI. The MN River sampler was housed within the USGS monitoring station on 16th Street in Milwaukee, WI (USGS station number 04087142). The KK River sampler was housed within the USGS monitoring station on 11th and Harrison Streets in Milwaukee, WI (USGS station number 04087159).

Flow data retrieval

River streamflow was retrieved from USGS continuous monitoring stations on the KK River at 11th Street (USGS station number 04087159), the MN River at Wauwatosa, WI (USGS station number 04087120), and the MKE River at Milwaukee, WI (USGS station number 04087000), which are represented in S2 Table. MN River at Wauwatosa, WI streamflow measurements were multiplied by a drainage area ratio correction of 1.0976 and MKE River at Milwaukee, WI streamflow measurements were multiplied by a drainage area ratio correction of 1.0117 to

correct for the difference in watershed area between the continuous monitoring stations and the locations at which samples were collected. Streamflow at the Milwaukee estuary was calculated by summing the instantaneous streamflow values of the MKE, MN, and KK Rivers and multiplying the streamflow of the MN and KK Rivers by a drainage area ratio correction of 1.229.

qPCR analysis

For qPCR, standard curves were run with DNA serially diluted from 1.5×10^6 to 1.5×10^1 copies per reaction and standards were run in triplicate. HB, Lachno2, and ruminant assay slopes, intercepts, and efficiency are shown in S3 Table. Each qPCR reaction results in a raw number of copies (CN) per reaction, which is then converted to a concentration of copies per 100 mL of original sample based on the proportion of the sample used in the reaction. For samples collected in 2014, 200 mL was filtered for all samples. For samples collected in 2015, 200 mL was filtered for all storm events and 400 mL was filtered for low flow samples. The limit of reliable quantification was determined to be 15 copies per reaction, or 225 CN/100 mL. Therefore, any samples with positive amplification, but were below 15 copies were reported as below the limit of quantification (BLQ).

Rainfall data retrieval

For the majority of events, average one-hour rainfall accumulation was computed for each watershed-defined area using radar-indicated rainfall models, retrieved from the National Weather Service North-Central River Forecast Center (National Weather Service, 2015). For events during which rainfall amounts from the National Weather Service were missing, the Thiessen polygon method was used in ESRI ArcGIS® to determine weights that were placed on MMSD rain gauges in each watershed. Rainfall depths from each rain gauge within each watershed were multiplied by their respective weights and these values were summed to compute average hourly rainfall depths across each watershed.

Complications to load and mass balance computations in a freshwater estuary

Although samples were collected in the KK, MN, and MKE Rivers and the Milwaukee estuary during nearly the same time periods, due to the complexity of the Milwaukee estuary,

determining a mass balance between the sewage loads measured in the rivers and the loads measured in the estuary is more complicated than simply adding up the loads from the three rivers and expecting it to be equal to loads measured in the estuary. Because of the proximity of the Milwaukee estuary to Lake Michigan, there is a significant amount of backflow which dilutes the concentrations measured in the samples collected in this study. The sampling location in the Milwaukee estuary is upstream of where the effluent from the Jones Island water reclamation facility is discharged, yet backflow could cause some of this effluent to flow upstream and potentially contribute to the concentrations measured in the estuary. Another issue is the distance between each sampling locations on the rivers and the sampling location in the estuary. Each of the rivers has a different velocity, effecting the travel time between the river monitoring sites and the estuary monitoring site. The bacteria we are measuring in the rivers may degrade or settle into the sediment before reaching the estuary, and likewise, bacteria can be re-suspended from the sediment into the water column. Settling could be significant due to the slowing of velocity in the estuary where the channels cross sections are much larger, and water surface slopes decrease due to influence from Lake Michigan. Additionally, samples are collected in the estuary from a relatively shallow intake of approximately two meters below the surface, meaning there is a potential for bacteria to be settled further down in the water column before reaching our sampling point. Finally, additional contributions from the urban areas located between the sampling locations on the rivers and in the estuary were not accounted for; however, for most events the sum of the loads from the three rivers were greater than the load in the estuary, suggesting settling and dilution outweighs these additional contributions.

References

Jin, S., Yang, L., Danielson, P., Homer, C., Fry, J., Xian, G., 2013. A comprehensive change detection method for updating the National Land Cover Database to circa 2011. Remote Sens. Environ. 132, 159–175. https://doi.org/10.1016/j.rse.2013.01.012

National Weather Service, 2015. National Weather Service, Advanced Hydrologic Prediction Service.

Xian, G., Homer, C., Dewitz, J., Fry, J., Hossain, N., Wickham, J., 2011. Change of Impervious Surface Area Between 2001 and 2006 in the Conterminous United States. Photogramm. Eng. Remote Sensing 77, 758–762.
