# Supplementary material for: High levels of sewage contamination released from urban areas after storm events: A quantitative survey with sewage specific bacterial indicators
Source: PLoS Med. 2018 Jul 24;15(7):e1002614. doi: 10.1371/journal.pmed.1002614 (PMC6057621; doi:10.1371/journal.pmed.1002614)
Supplement: S2 Text — QAPP, Quality Assurance Project Plan. (PDF) [file pmed.1002614.s002.pdf]

Identification and Quantification of Sanitary Sewage Contamination in the Milwaukee Estuary Area of Concern

Quality Assurance Project Plan

EPA Grant Funding Source: GLRI
Grant #: GL00E01206 sub 3

Project Coordinator: Name: Dr. Sandra McLellan
Affiliation: UW – Milwaukee School of Freshwater Sciences
Address: 600 E. Greenfield Ave.
Milwaukee, WI 53204

Principal Investigator: Sandra McLellan
Co-Investigations: Deb Dila, Hayley Templar

Prepared: 08/04/2014
Revision #: 0

**Approvals:**	**Date:**
Sandra McLellan, Project Coordinator, UWM - SFS	8-4-14
Hayley Templar, UWM - SFS	8/4/14
Deb Dila, Research Specialist (Laboratory), UWM - SFS	8/4/14
Cheryl Nenn, Milwaukee Riverkeeper	8/14/14
Stacy Hron, Milwaukee Estuary AOC Coordinator, WDNR	
Donalea Dinsmore, WDNR Great Lakes Quality Assurance Coordinator	
Jennifer Conner, GLNPO Grant Manager	

TABLE OF CONTENTS

Distribution List....3
Executive Summary....4
A. Project Organization....5
  Problem Definition/Background:....6
  Project Objectives....7
  Project/Task Description and Schedule....7
    Tasks....10
    Schedule.... **Error! Bookmark not defined.**
    Personnel, Special Equipment or Supplies....12
  Special Training Requirements or Certifications....12
  Documentation and Records....12
    Field Records....12
    Laboratory Records.... **Error! Bookmark not defined.**
    Project Records....12
    Final Report....12
    Project File Final Disposition and Record Retention....13
B. Measurement/Data Acquisition....14
  Sample Process Design (Experimental Design)....14
  Sampling Method Requirements....19
  Sample Handling and Custody Requirements....19
  Analytical Requirements....20
  Quality Control Requirements....21
  Data Management....21
C. Assessment/Oversight....21
  Assessments and Response Actions....22
  Reports to Management....23
D. Data Validation and Usability....24
  Data Review, Validation, or Verification....24
  Reconciliation with Data Quality Objectives....24

Distribution List

PDF copies of the approved QAPP and any revisions will be sent to everyone below unless a hard copy is requested:

1. Sandra McLellan, Professor, School of Freshwater Sciences, University of Wisconsin-Milwaukee
2. Donalea Dinsmore, Great Lakes Funding and Quality Assurance Coordinator, WDNR
3. Stacy Hron, Milwaukee Estuary Area of Concern Coordinator, WDNR
4. Jenny Kehl, Associate Professor and Director of the Center for Water Policy, School of Freshwater Sciences, University of Wisconsin-Milwaukee
5. Steve Corsi, Research Hydrologist, U.S. Geological Survey
6. Cheryl Nenn, Riverkeeper, Friends of Milwaukee Rivers
7. Deb Dila, Research Specialist, School of Freshwater Sciences, University of Wisconsin-Milwaukee

Note: Donalea Dinsmore will provide an electronic copy of the QAPP to the GLNPO grant manager and QA Track system.

Executive Summary

This project seeks to delineate fecal pollution sources entering the Milwaukee Estuary Area of Concern (AOC), identify the presence of human sewage pollution entering Lake Michigan from the estuary, and quantify the sewage pulse entering the estuary from the Milwaukee, Menomonee, and Kinnickinnic Rivers. The Milwaukee Estuary has been classified as an AOC since the late 1980s. Eleven of the fourteen possible Beneficial Use Impairments (BUIs) are listed for the estuary. Although there are many causes of these impairments, point source and runoff pollution is a major cause of each of them. Sanitary sewer (SSO) and combined sewer overflows (CSO) are of concern as point source fecal coliform pollution; however, overflows have not been as great of an issue since the installation of the Milwaukee Metropolitan Sewerage District's (MMSD) Deep Tunnel stormwater storage system in the early 1990s. Water quality modeling of nonpoint source runoff in the rivers entering the Milwaukee Estuary have predicted levels of fecal coliform much lower than the observed levels, suggesting that a large portion of fecal coliforms are from other sources (point source pollution from illicit sewage/industrial discharges, leaking sewage or combined sewage lines, malfunctioning sewage gates, etc.). Because these high fecal coliform levels are observed in the absence of a CSO or SSO, it is evident that there must be more to the story of fecal pollution in the rivers and estuary. Fecal coliform levels by themselves are not indicative of the source of pollution (human or non-human). Human-specific *Bacteroides* and *Lachnospiraceae* are two genetic markers used to quantify the amount of sewage pollution present in a sample. This project will identify and map unrecognized sanitary sewage contamination and determine the contribution of sewage to pathogen and fecal indicator loads to the Milwaukee Estuary. Fecal coliform and sewage loads will also be calculated for the Milwaukee Estuary, and Milwaukee, Menomonee, and Kinnickinnic Rivers. Findings will be transferred to local municipalities responsible for mitigating sanitary sewage discharges, to water resource managers working on Total Maximum Daily Loads (TMDLs), and to the Department of Natural Resources (DNR) to support their watershed-based permitting efforts. This information will be a useful steppingstone for local agencies such as MMSD, as well as agencies at the state and federal level to create appropriate TMDLs and Remedial Action Plan (RAP) goals to delist BUIs.

A. Project Organization

Table 1 - Roles and Responsibilities

Individuals Assigned:	Responsible For:	Authorized To:
Sandra McLellan - Professor and Senior Scientist UWM-SFS	Principle Investigator. Responsible for overall project execution and reporting.	Write reports and submit to EPA. Perform implementation tasks and inform others how to do so. Supervise graduate students. Prepare findings for scientific publication.
Steve Corsi - Research Hydrologist U.S. Geological Survey	Responsible for USGS Samples and associated analysis.	Supervise field technicians and all USGS analysis of samples. Prepare findings for scientific publication.
Jenny Kehl - Director of the Center for Water Policy and Associate Professor UWM-SFS	Responsible for dissemination of research findings, including presentations and publications, to end-users.	Supervise graduate students. Prepare outreach materials for end-users.
Hayley Templar - Graduate Student UWM-SFS	ISCO automated sampler fieldwork and analysis of sewage contamination loads in the Menomonee, Kinnickinnic and Milwaukee rivers and the Milwaukee estuary.	Supervise undergraduates doing field sampling. Perform fieldwork and microbiological analysis. Assist with preparation of findings for publication.
Cheryl Nenn - Riverkeeper - Milwaukee Riverkeeper	Outfall project coordination and Riverkeeper outreach.	Milwaukee Riverkeeper project coordination. Supervise Riverkeeper personnel.
Joe Rath - Water Quality Specialist - Milwaukee Riverkeeper	Outfall sampling fieldwork and GIS mapping.	Supervise outfall collection fieldwork. Perform outfall collection fieldwork and GIS mapping of data. Review data for accuracy.
Melinda Bootsma - Laboratory Technician UWM-SFS	Genetic marker assays and QA/QC on samples.	Supervise students doing PCR and qPCR. Perform PCR, qPCR, fieldwork and microbiological analysis.
Deb Dila - Research Specialist UWM-SFS	QA/QC management, communication with MMSD and assist with preparation of findings for publication. Posting end-user publications to McLellan Lab website. Quarterly invoicing through UWM accounting office.	Supervise fieldwork and microbiological analysis. Perform fieldwork and microbiological analysis. QA/QC management, communication with MMSD, post end-user publications online and assist with preparation of findings for scientific publication. Send quarterly invoice breakdown to accounting office for submittal to WDNR.
Donalea Dinsmore – Great Lakes Quality Assurance Coordinator	Approve project quality documentation consistent with grant requirements, GLAS updates, coordinate semi-annual grant reporting	Communication with project team and GLNPO, approve changes to project quality documentation

Organizational Chart

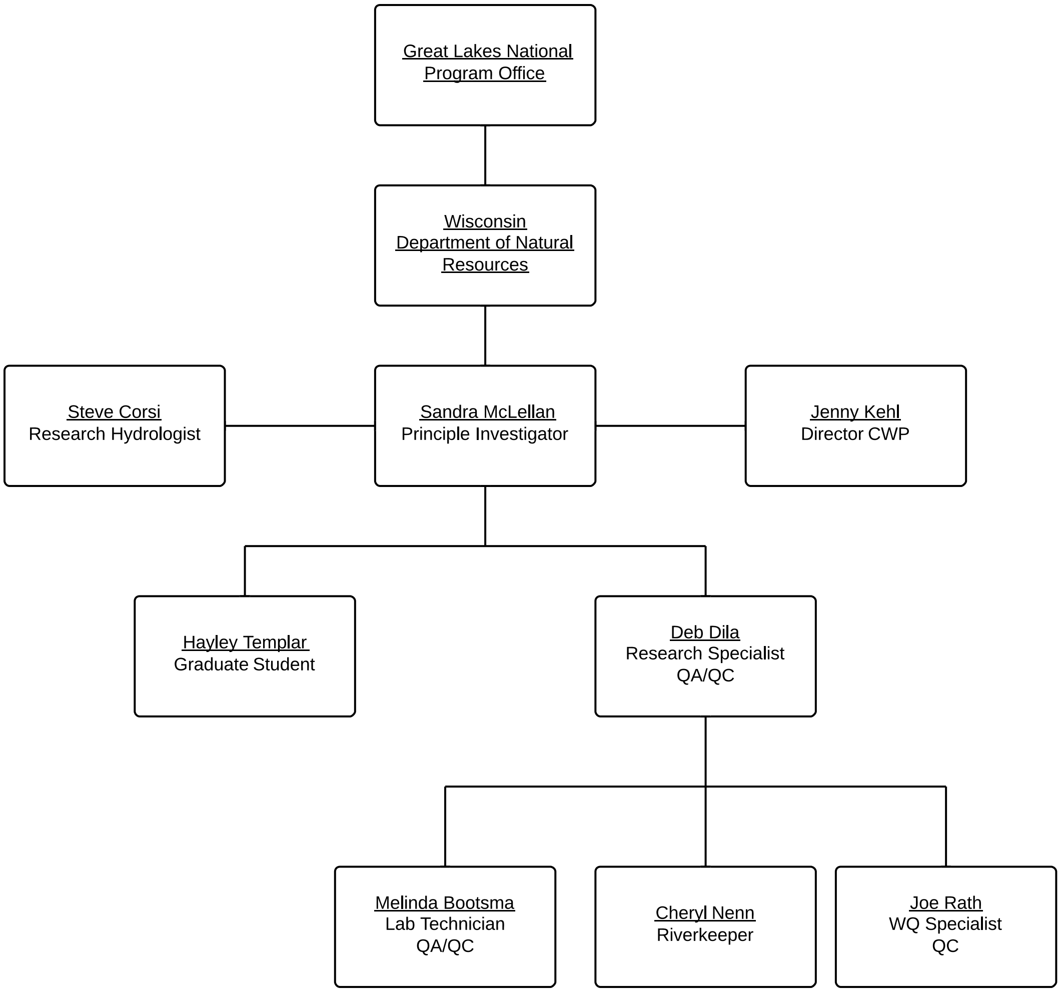

Problem Definition/Background:

Despite substantial improvements in sewage treatment in the Milwaukee Estuary AOC, water quality standards for recreation are still regularly exceeded in the AOC, and pose a significant challenge to removing the beach closings and recreational restrictions impairment. The cause of these exceedances is largely attributed to contamination by urban stormwater. High levels of fecal indicator bacteria have been found in urban stormwater discharges and are the largest contributor to water quality impairments for bacteria in Milwaukee's urban rivers. Water quality models have shown that 60-75% of the fecal coliform loads cannot be explained by nonpoint source runoff from rooftops, parking lots, streets, and other impervious surfaces, especially for the Menomonee and KK Rivers. Preliminary data demonstrates that exfiltration (leaking) from failing sanitary sewer infrastructure is a major source of fecal indicator bacteria and pathogens in urban stormwater that impacts the AOC. This means that stormwater systems are acting as conduits for conveying sewage from failing infrastructure into surface waters used for drinking water and recreation. Sanitary waste poses a more direct threat to human health, since it is more likely to contain pathogens than urban stormwater runoff. This problem is particularly difficult to address because potentially thousands of localized breeches could be involved. Therefore, completing this project to identify and quantify sanitary sewage contamination of stormwater in the AOC provides a crucial, and currently missing, link in efficiently and effectively addressing the beach closings and recreational restrictions impairment.

Project Objectives

Objectives

The overall goal of our project is to delineate fecal pollution sources entering the AOC. A major part of this project is to identify unrecognized sanitary sewage contamination and determine the contribution of sewage to pathogen and fecal indicator loads to the Milwaukee Estuary. This information is necessary to direct mitigation efforts towards reducing pathogens in the AOC. The specific objectives in this proposal are critical elements in our overall efforts and they address data needs that are not currently funded, or are only partially funded. Below are specific objectives and a brief description of each.

Objective 1. *Map and sample stormwater outfalls along the lower Menomonee and Kinnickinnic and perform up the pipe investigations to identify illicit discharges.*

This objective will provide a comprehensive map of illicit discharges in the lower Menomonee and Kinnickinnic River watersheds, which can be used as a resource by municipalities. Human-specific fecal indicators will be quantified in terminal outfalls and results mapped using GIS and will include drainage areas, elevations, and sewer configuration. We will also map age of development and use these different variables to determine correlations to "high likelihood of failing infrastructure" (Figure 2). Outfalls will be prioritized based on the concentration and load of human-specific indicators and up the pipe investigations conducted in collaboration with municipalities and the Milwaukee Metropolitan Sewerage District (MMSD).

The analytical methods for the human-specific indicators *Bacteroides* and *Lachnospiraceae* have been previously described by our laboratory (Newton et al., 2011). We are also developing methods for new assays that target non-human sources of fecal pollution and will incorporate this testing as appropriate. These new markers will continue to improve our resolution in confirming, and positively identifying other sources of fecal pollution in stormwater (e.g., urban wildlife). Concentrations determined by the analytical quantitative polymerase chain reaction (qPCR) method help to determine the extent of human sewage contamination, and can be used in conjunction with storm sewer drainage areas as a proxy for pathogen loads.

We have mapped 185 different terminal outfalls to date, which represents 70% coverage of the terminal outfalls in the lower Menomonee River (between Burleigh Ave and Hawley Ave) and 10% coverage of the Kinnickinnic watersheds. We found 80 of these terminal outfalls (>40%) have low to moderate levels of sewage contamination and 28 have very high levels of sewage contamination (15%). In this project, we would complete the mapping of the lower Menomonee and KK watersheds and conduct a minimum of 15 up-the-pipe-investigations each year, targeting areas of the highest priority, e.g. sites with the highest human fecal pollution signal and/or load. In all, we anticipate analyzing 150 terminal outfalls and 150 up-the-pipe samples using traditional microbiology and qPCR for source-specific indicators over 2013-2015 Up-the-pipe sampling is important in helping to better define the location of infrastructure failure within the storm sewer drainage area that leads to each terminal stormwater outfall at the river discharge location, and MMSD has been conducting this sampling as an in-kind contribution to this project. This effort would provide comprehensive coverage for the two most urbanized watersheds impacting the AOC.

Objective 2. *Quantify sewage contamination in the Milwaukee Estuary and Milwaukee, Menomonee, and Kinnickinnic Rivers.*

Our ongoing sampling program with USGS will allow us to collect integrated water samples across the hydrograph at downstream locations in the Milwaukee, Menomonee, and Kinnickinnic Rivers. We have been quantifying human-specific markers and general indicators at the channel leading to Lake Michigan (Figure 3). There is a clear human signal from these watersheds in the absence of combined or sanitary sewage overflows.

In collaboration with USGS, we will deploy ISCO sequential samplers at an estuary site (Jones Island), the Kinnickinnic River (11th and Harrison), the Menomonee River (16th Street), and the Milwaukee River (Cherry Street). The downstream river locations will provide a critical dataset for the evaluation of fecal bacteria loads just prior to the estuary and the estuary site will provide a critical dataset for calculation of bacterial loads just prior to Milwaukee's inner harbor.

As sewage-contaminated waters are closely associated with human pathogens, the cause of the beach closings and recreational restrictions impairment, we will analyze baseflow and storm event samples collected from 2012 through 2014 to determine the relative contribution of sewage sources to the overall fecal coliform levels. We will use the same analytical procedures (microbiology and qPCR) used for outfall sampling to differentiate the two forms of pollution.

Objective 3. *Fill data gaps and interface with TMDL efforts to prioritize implementation strategies.*

Current Great Lakes Restoration Initiative funding is supporting TMDL development for the three watersheds within the Milwaukee River Basin and estuary. This project is designed to meet a major need for TMDLs, i.e. identification of sources.

For the TMDLs, load calculations are based upon fecal coliforms for the watersheds and *E. coli* for the estuary. However, unrecognized sanitary inputs contribute additional fecal coliforms and *E. coli* beyond what is estimated from land use and runoff calculations. Comprehensive mapping and river sampling will allow priority ranking of sites suspected as major contributors to sewage derived TMDLs and result in more effective remediation of both local and downstream loads, ultimately targeting the pathogens that give rise to one of the estuary's beneficial use impairments.

Historically, concentrations of fecal coliform bacteria in the estuary portions of the Kinnickinnic, Menomonee, and Milwaukee Rivers regularly exceeded the estuary variance standard of 1,000 CFU/100 ml. According to MMSD datasets, between 1975 and 2004 the median concentration of fecal coliform bacteria in the Milwaukee Harbor estuary was about 930 CFU/100 ml. Fecal coliform counts in the estuary varied over seven orders of magnitude during this period (from 1 CFU/100 ml to 2,400,000 CFU/ 100 ml), regularly exceeding the variance standard and almost always exceeding the standard for full recreational use (200 CFU/100 ml). From 2000-2002, MMSD measured levels of *E. coli* in the estuary varied over six orders of magnitude, ranging from <1 CFU/100 ml to 240,000CFU/100 ml.

Current MMSD data illustrates the substantial and ongoing contamination problems in the AOC. In 2012 during June, July, and August, 53% of MMSD samples collected in the estuary (n=30) had fecal coliform levels greater than 200 CFU/100 ml and 10% had levels greater than 1000 CFU/100 ml. The *E. coli* levels were greater than 235 CFU/100 ml in 20% of the samples and 7% were greater than 1000 CFU/100 ml,

Wisconsin standards for water quality advisories and beach closings respectively. Importantly, 2012 was during an extreme drought, thereby representing a "best case" scenario for storm-driven pollution levels.

MMSDs comprehensive sampling program provides ongoing data for the AOC and the upstream rivers that impact the AOC. This sampling program is the primary data source for developing the TMDLs and sites correspond to assessment points used in the TMDL. As the McLellan lab became engaged in the TMDL project, they requested split samples from MMSD's monitoring program in the Kinnickinnic and lower Menomonee rivers, whereby MMSD took 2 sets of samples—one for their lab and one for the McLellan lab. This has enabled the McLellan lab to archive an entire sampling season with minimal budget investment so that these samples would be immediately available for analysis during winter of 2012/2013. As a result, more than 200 samples have been analyzed for *E. coli* and enterococci by culture methods and archived by freezing samples for later qPCR testing. We will obtain river samples from MMSD in 2013 and each year analyze the most relevant samples by qPCR. We estimate that we will use qPCR to analyze a max of 200 in-stream samples**.**

Center for Water Policy

The second part of this objective is to interface with the Center for Water Policy to disseminate our findings to inform policy. We will dedicate one graduate Master's student to these efforts, who can help translate our findings into information useful in other efforts in our region. The project results will be translated into research briefs and policy briefs designed to be useful for decision makers, stakeholders, municipalities, and water resource managers. The articulation and communication needs will be assessed for targeted groups. The graduate student will generate the briefs under the guidance of Dr. Kehl, who will review them with and the project team. The results will be disseminated to decision-makers at MMSD and the WDNR, as well as through stakeholder meetings, links to research briefs, and direct access to policy briefs, providing for feedback loops.

Project/Task Description and Schedule

Tasks and Schedules

Objective 1 will provide a comprehensive map of illicit discharges from terminal stormwater outfalls in two urbanized watersheds, which can be used as a resource by municipalities.

Objective 1 Tasks and Schedule:

1. Outfall sample collection by Milwaukee Riverkeeper.
2. Up-the-pipe investigations of outfalls determined to be high priority.
3. Analyze samples using culture methods and quantitative polymerase chain reaction (qPCR) to determine concentrations of traditional indicators (*Escherichia coli*, enterococci, and total fecal coliforms) and human sewage indicators (Human-specific *Bacteroides* and *Lachnospiraceae*).
4. Statistical analyses and mapping of data:
   a. Correlations to drainage area, elevations, and sewer configuration
   b. GIS map human sewage indicator concentrations

Task	Schedule
1. Outfall Sampling (Riverkeeper)	April-October 2014 and 2015
2. Up-the-pipe Sampling (MMSD)	April-October 2014 and 2015
3. Sample Processing/Analysis	April-November 2014 and 2015
4. Data Analysis	October-December 2014 and 2015

Objective 2 will quantify sewage contamination loads in the Milwaukee, Menomonee, and Kinnickinnic Rivers and in the Milwaukee Estuary.

Objective 2 Tasks and Schedule:

1. Sample storm events, baseline conditions, and any sewer overflow events using automated samplers at Jones Island wastewater treatment plant (Milwaukee Estuary site) and one location on each of the Milwaukee, Menomonee, and Kinnickinnic Rivers. A storm event will be sampled if rainfall amounts are a tenth of an inch or greater in the urban areas of each river's watershed.
2. Analyze samples using culture methods and qPCR to determine concentrations of traditional indicators (*Escherichia coli*, enterococci, and total fecal coliforms) and human sewage indicators (Human-specific *Bacteroides* and *Lachnospiraceae*).
3. Calculate sewage loads using human sewage indicator concentrations and discharge from the USGS gauging stations at or near each sampling location.

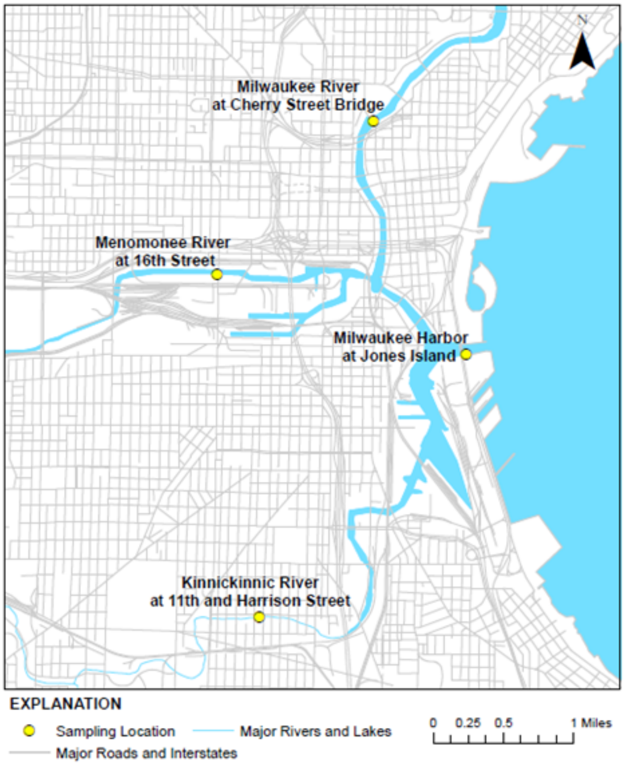

Task	Schedule
1. ISCO sampling	March-October 2014 and 2015
2. Sample processing/Analysis	March-November 2014 and 2015
3. Data analysis	October-December 2014 and 2015

Objective 3 will fill data gaps and interface with TMDL efforts to prioritize implementation strategies by identification of sources.

<u>Objective 3 Tasks and Schedule:</u>

1. Interface with TMDL investigators to determine river assessment points targeted for load reductions.
2. Based on assessment points, select relevant archived DNA samples for qPCR analysis. Assay for general fecal indicator bacteria and human sewage indicators (Human-specific *Bacteroides* and *Lachnospiraceae*).
3. Calculate bacterial loads near assessment points.
4. Map river hot-spots based on bacterial loads and map nearby terminal stormwater outfalls.
5. Interface with the UWM Center for Water Policy to create research briefs, policy briefs, and science-based policy solutions.
6. Communication: Inform policy-makers, stakeholders, municipalities, and water resource managers of these findings. Post research and policy briefs on McLellan Lab website.

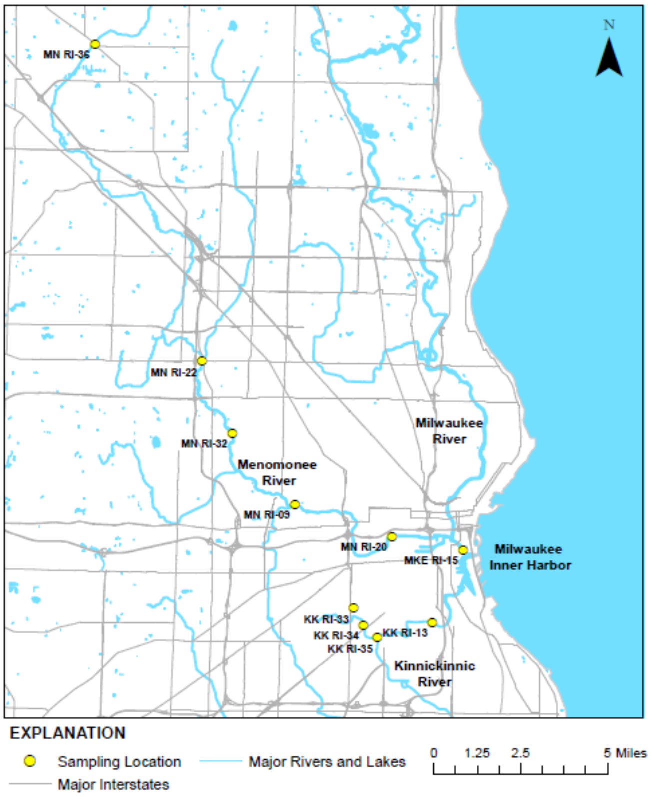

Task	Schedule
1. Get load reduction assessment points	January 2014
2. Select archived DNA for analysis/assay for genetic markers	January-March 2014
3. Calculate bacterial loads near assessment points	March-May 2014
4. Map river hot-spots	May-September 2014
5. Center for Water Policy data assimilation	October 2014-January 2015
6. End-user communication	February 2015-July 2015

Special Equipment or Supplies

We require four ISCO 3700 automated samplers with the 24 1L bottle configuration (for Objective 2).

Personnel, Special Training Requirements or Certifications

Technicians, graduate students, undergraduate students, and volunteers in the McLellan Lab are provided with specific training in the collection and laboratory analysis of microbiology samples prior to beginning work in the lab. Training is given to ensure sample collection in the field and sample processing in the lab are performed according to the appropriate SOPs (Routine sample collection and processing procedures are described in Appendices A-1, B-1, and B-2). Training on the operation of ISCO automated samplers will be provided by USGS, to ensure that the samplers are operated and samples are collected according to the appropriate SOPs (ISCO operation and sample collection are described in Appendix A-2). Trained and experienced technicians will conduct qPCR analysis according to the appropriate SOP (qPCR procedures are described in Appendix B-4).

Documentation and Records

Field and Laboratory Records

For samples collected by MMSD or Riverkeeper, sample site data is collected by the field technician and is included on the label for each sample, which is removed and saved for future reference after sample processing is complete. McLellan lab personnel use specialized field sheets to record necessary information during the collection of samples from ISCO automated samplers (Field sheets used for ISCO sampling are represented in Appendix D). Samples from MMSD were tracked by their Laboratory Information Management System (LIMS) number and associated with a McLellan Lab Fate and Transport (FT) number. Samples collected by Milwaukee Riverkeeper or the McLellan Lab are assigned an FT number for tracking. Sites also have location informative site codes assigned to them by the group sampling them (i.e., MMSD, Riverkeeper or McLellan Lab). Laboratory personnel will record when the sample is received and tested. Data including field crew, water body, sampling point, date/time of collection and weather conditions are entered into a field logbook. Test results are entered into a laboratory notebook and then transferred to an MS Access® database and Excel® spreadsheets. Database entries are reviewed for accuracy by the McLellan Lab project manager and the Riverkeeper field manager.

Project Records

Project records will include documentation of original proposal, scope of work for McLellan Lab and Riverkeeper, letters of commitment, FT sheets, field sheets, field logbooks, laboratory notebooks containing data results, quarterly reports, policy briefs, updated Stormwater Report and mapped data for print and online materials.

Final Report

A comprehensive final report will be prepared for submission to the Wisconsin Department of Natural Resources. The report will include details of findings for each objective and will summarize the conclusions we reach.

Objective 1. Graphs and/or tables will compare and rank (according to definitions previously described in the 2008-2012 Greater Milwaukee Stormwater Report) the sewage pollution signal found in stormwater

from ~150 terminal outfall samples and ~150 up-the-pipe samples. Maps of sewage contamination levels will be included.

Objective 2. Graphs and/or tables will compare watershed sewage loads calculated from river and estuary samples collected during a variety of hydrographic conditions. A minimum of 200 samples will be qPCR analyzed for sewage markers to make these calculations.

Objective 3. Graphs and/or tables will compare levels of sewage pollution found in ~200 archived river samples corresponding to TMDL assessment points. Maps will be included to highlight areas with suspected sewage sources as major contributors to fecal coliform loads and beneficial use impairments. Related policy briefs, created by the Center for Water Policy, will be included to convey findings and educate water resource managers and the general public.

Project File Final Disposition and Record Retention

Storage, access to, and final disposition of all records are subject to the requirements of Wisconsin Department of Natural Resources.

Raw data will be stored in the McLellan Lab Microsoft Access database on a MySQL server that is backed up daily at UWM-School of Freshwater Sciences. Data is retained in the McLellan database for no less than five years.

B. Measurement/Data Acquisition

Sample Process Design (Experimental Design)

Site Selection Criteria	Objective 1	Objective 2	Objective 3
Consultation with stormwater group*	•		
Concentration of human sewage indicators	•		
Frequency of contamination	•		
Contaminated outflow during dry weather	•		
Small drainage areas	•		
Urban river reach		•	
Secure locations for equipment		•	
Power source		•	
Part of MMSD twice monthly WQR monitoring program			•
Correspond to TMDL assessment points			•

*Staff from McLellan Lab, Riverkeeper and MMSD meet quarterly to review recent results and prioritize outfalls and pipes.

Mapping illicit discharges from terminal stormwater outfalls in two urbanized watersheds (Objective 1):

Milwaukee Riverkeeper will complete the terminal outfall sample collections. Sampling will focus on outfalls in the Kinnickinnic River watershed, but will also include outfalls of concern in the Menomonee River watershed. A total of 150 grab samples will be collected. The majority of sampling will be during rain events, when sewage contaminated stormwater pipes show evidence of human fecal genetic markers in their outflow. Additionally, at least one dry weather survey will be completed each field season to identify outfalls that leak sewage during dry weather. (Sampling procedure is detailed in Appendix A–1)

After analysis of outfall samples for sewage contamination, as measured by human fecal markers, Metropolitan Milwaukee Sewerage District (MMSD) will conduct up-the-pipe investigations of high priority outfalls to pinpoint contamination sources. Prioritization for this follow-up investigation is based on: 1) concentration of human-specific *Bacteroides* and *Lachnospiraceae* in outfall sample, 2) frequency of outfall contamination and 3) contaminated outflow during dry weather. Outfalls with small drainage areas are also given preference for follow-up as locating the source of contamination can be faster. 150 up-the-pipe samples will be collected using both automated sampling and grab sampling techniques. Automated samplers will be used during wet weather sampling and grab samples will be taken during dry weather sampling. Dry weather samples will be taken from pipes that leak sewage during dry weather. (Sampling procedure is detailed in Appendix A–3)

Milwaukee Riverkeeper and MMSD will return all samples on ice to the McLellan Lab. Samples will be analyzed using plate culture methods and quantitative polymerase chain reaction (qPCR) to determine concentrations of traditional indicators (*Escherichia coli*, enterococci, and total fecal coliforms) and human

sewage indicators (Human-specific *Bacteroides* and *Lachnospiraceae*). (Laboratory procedures are detailed in Appendices B–1, B–2, B–3, and B–4).

GIS mapping and maintenance of GIS databases will be conducted by Milwaukee Riverkeeper. They will maintain and improve an existing GIS database and update maps with current measures of human fecal contamination at terminal outfalls. (GIS database and mapping procedures are detailed in Appendix C–1).

Quantifying sewage contamination loads in the Milwaukee, Menomonee, and Kinnickinnic Rivers and in the Milwaukee Estuary (Objective 2):

Storm events, baseline conditions, and sewer overflow events will be sampled using automated samplers located in the Milwaukee Estuary Channel and in the Milwaukee, Menomonee, and Kinnickinnic Rivers. The sampler in the Milwaukee Estuary Channel will be located within the USGS gauging station at Jones Island water reclamation facility. The sampler in the Milwaukee River will be located within an MMSD real-time station beneath the Cherry Street Bridge. The samplers in the Menomonee River and Kinnickinnic Rivers will be located within the USGS gauging stations on 16th Street and 11th Street, respectively. These locations were chosen because each have a secure structure to prevent damage to the equipment, each has a power source, and they are each in locations which allow us to capture much of the urban area influencing the rivers.

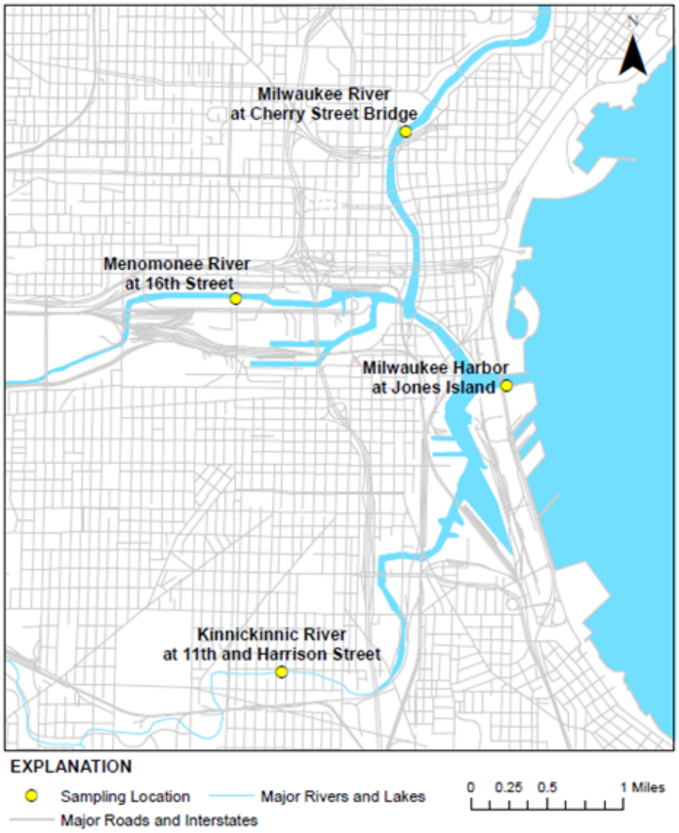

Each site will contain one ISCO 3700 automated sampler. Sample lines will be run from the ISCOs into the river and attached to a permanent structure. Samplers will be remotely triggered to begin sampling before predicted rain events to capture changes in levels of sewage contamination as measured by human-specific *Bacteroides* and *Lachnospiraceae.* Baseline conditions will measure levels of contamination present at these sites when there has been no precipitation for at least 48 hours prior. Any sewer overflow event will be followed for one to three days after the event, depending on weather conditions. (Sampling procedure is detailed in Appendix A–2).

Decision matrix for ISCO sample collection:

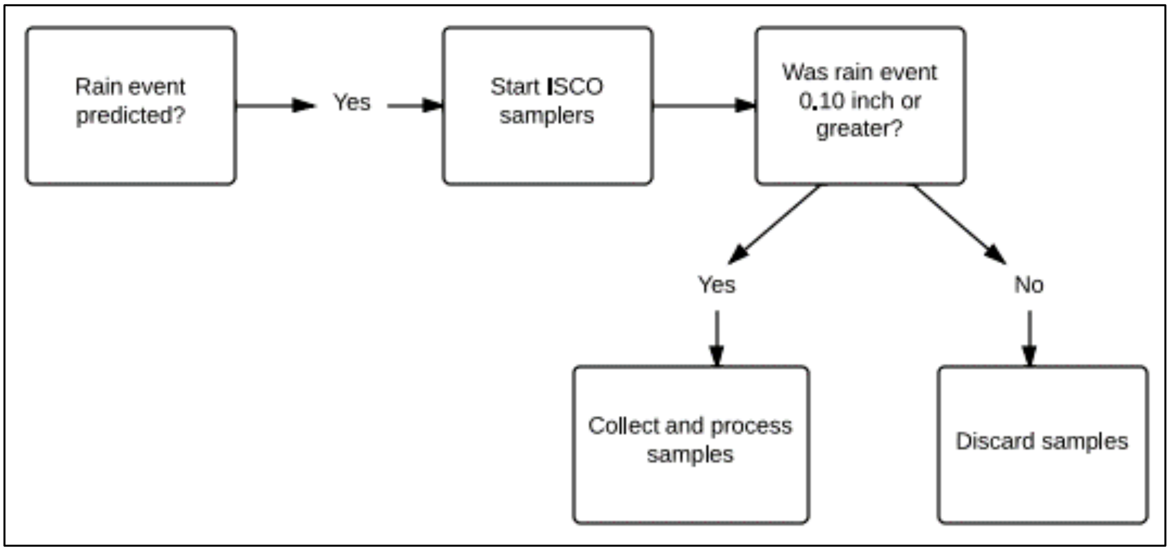

Samples will be analyzed using culture methods to determine concentrations of traditional indicators (*Escherichia coli,* enterococci, and total fecal coliforms). (Laboratory procedures are detailed in Appendices B–1, B–2, B–3, and B–4).

Decision matrix for sample processing and handling:

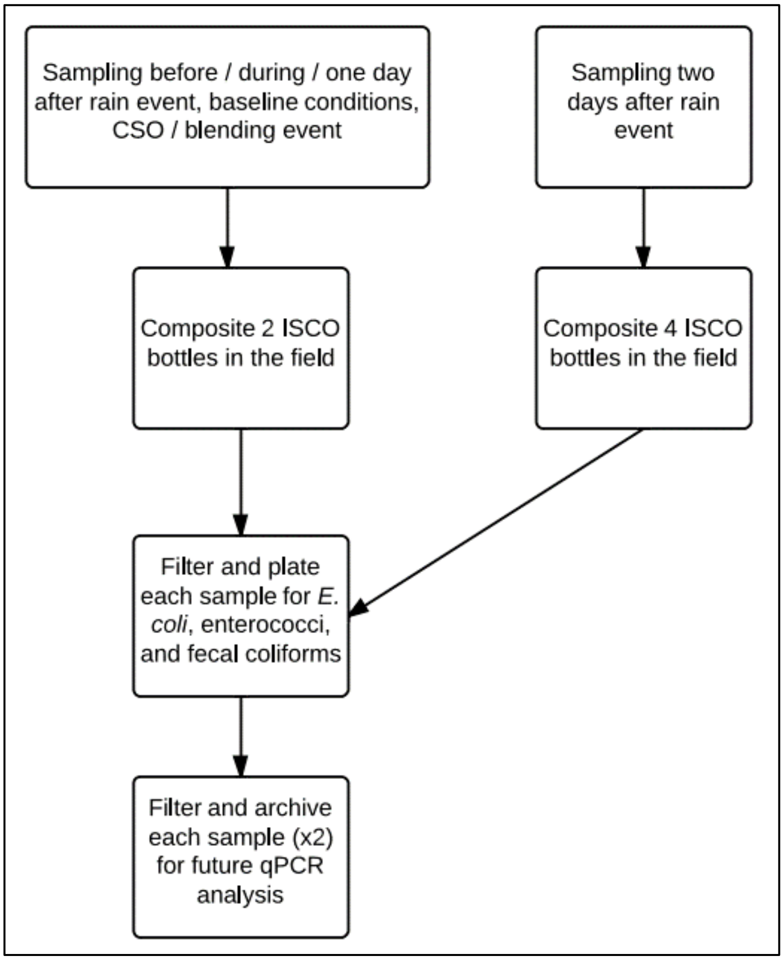

Quantitative PCR will be used to determine concentrations of human sewage indicators (Human-specific *Bacteroides* and *Lachnospiraceae*) using the laboratory procedures detailed in Appendix B-4. Samples will be evaluated based on plate counts of *E. coli* and enterococci and chosen for qPCR analysis depending on the characteristics of each events' hydrograph. Although each sample represents a two hour interval, not every sample will be chosen for qPCR analysis. Depending on their location in the hydrograph of the event, every sample, every other sample, or every 4 samples will be analyzed to represent the entire event. Additional samples may be chosen when further information is needed to analyze patterns across the hydrograph.

Decision diagram for qPCR analysis of ISCO samples:

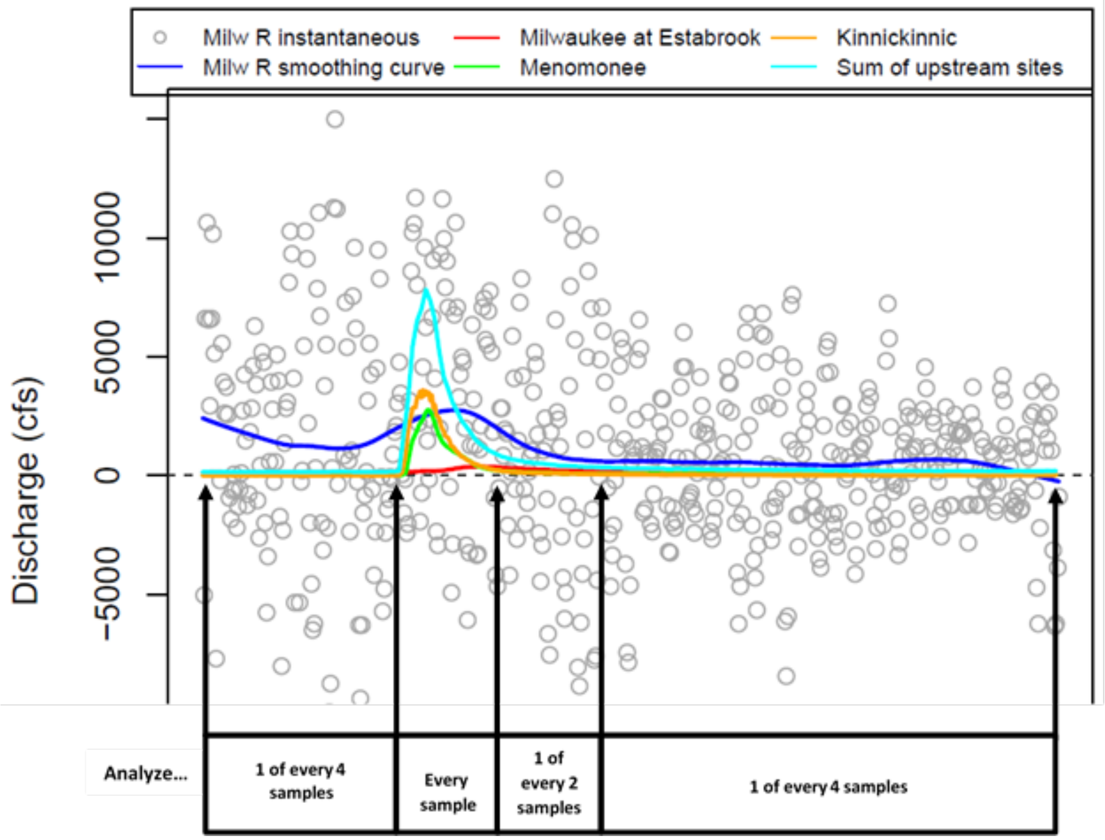

Overall a total of approximately 100 samples per year will be analyzed for human-specific indicators, as described by the following table:

**Number/Types of Events Analyzed**	**Number of Samples Analyzed per Event**	**Total Number of Samples Analyzed**
1 – 2 Baseline	4 – 6	10
4 – 5 Rain	25 – 50	50
1 – 2 CSO	25 – 50	40
Total		100

Sewage loads in each of the four locations will be calculated using human sewage indicator concentrations and discharge from the USGS gauging stations at or near each sampling location. Continuous discharge data from each USGS gauging station will be downloaded from the USGS National Water Information System (NWIS). Microsoft Excel, R, and other appropriate programs will be used to calculate instantaneous and event loads using human sewage indicator bacteria concentrations and downloaded discharge data.

Site	GPS Coordinates	
	Latitude	Longitude
Milwaukee Harbor at Jones Island	43°01'28"N	87°53'54"W
Milwaukee River at Cherry Street	43°02'55"N	87°54'41"W
Menomonee River at 16th Street	43°01'58"N	87°55'59"W
Kinnickinnic River at 11th and Harrison Street	42°59'50"N	87°55'36"W

Fill data gaps and interface with TMDL efforts to prioritize implementation strategies by identification of sources (Objective 3):

We will meet with TMDL investigators and key TMDL personnel (CDM Smith consultants) to determine river assessment points targeted for bacterial load reductions. Based on assessment point locations, we will select corresponding archived DNA samples collected during MMSDs 2012 and 2013 river sampling program. MMSD river sampling is a comprehensive year-round program and the primary data source for developing the Milwaukee area TMDLs. The river sampling sites correspond to assessment points used in the TMDL. More than 200 samples have been analyzed for *E. coli* and enterococci by culture methods and archived (by freezing) for follow-up qPCR testing. We will use qPCR analysis to assay for general fecal indicator bacteria (*E. coli* and enterococci) and human sewage indicators (human-specific *Bacteroides* and *Lachnospiraceae*). (Sampling procedure is detailed in Appendix A–4), (Laboratory procedures are detailed in Appendices B–1, B–2, B–3, and B–4).

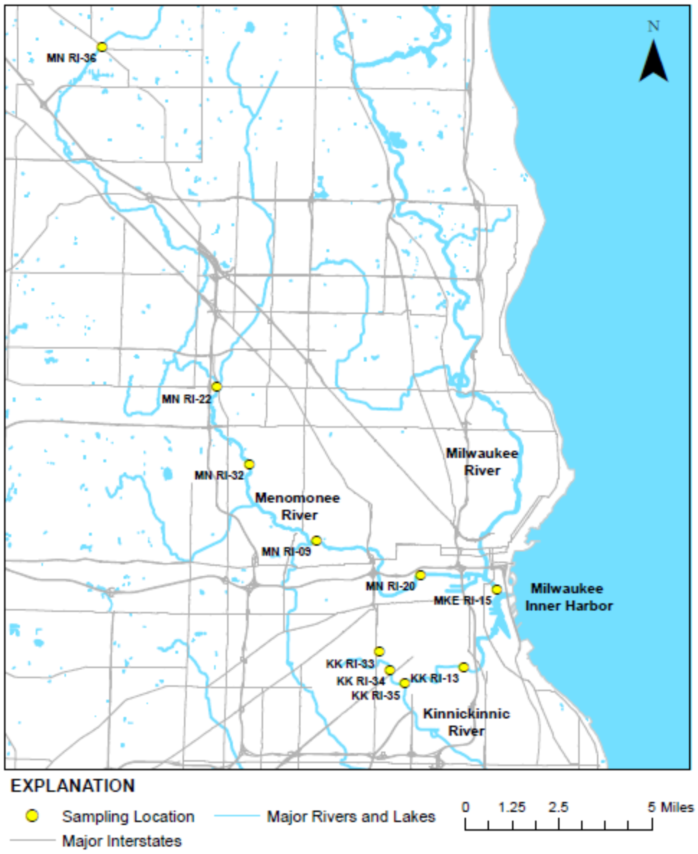

Following qPCR analysis for human-specific sewage indicators, bacterial loads will be estimated near assessment points. Based on loads, river "hotspots" and nearby terminal outfalls will be mapped. Comprehensive mapping will allow priority ranking of sites suspected as major contributors to sewage derived TMDLs and result in more effective remediation of both local and downstream loads, ultimately targeting the pathogens that give rise to one of the estuary's beneficial use impairments.

The results will be translated into research briefs and policy briefs designed to be useful for decision makers, stakeholders, municipalities, and water resource managers. The articulation and communication needs will be assessed for targeted groups. The results will be disseminated through stakeholder meetings, links to research briefs, and direct access to policy briefs, providing for feedback loops.

Site	GPS Coordinates	
	Latitude	Longitude
KK RI-13	42.59'48.9"	87.55'16.2"
KK RI-33	43.00'08.9"	87.57'57.8"
KK RI-34	42.59'42.4"	87.57'38.1"
KK RI-35	42.59'25.5"	87.57'08.5"
MKE RI-15	43.01'38.8"	87.54'15.6"
MN RI-09	43.02'43.8"	88.00'00.0"
MN RI-20	43.01'57.2"	87.56'40.7"
MN RI-22	43.06'16.8"	88.03'16.9"
MN RI-32	43.04'29.6"	88.02'11.3"
MN RI-36	43.14'10.5"	88.07'02.6"

Sampling Method Requirements

All sampling methods are detailed in Appendix A.

Sample Handling and Custody Requirements

Objective 1. Milwaukee Riverkeeper and MMSD will keep all field samples on ice until delivery to the McLellan Lab. In the field, samples will be collected and labeled by trained Riverkeeper or MMSD personnel. Riverkeeper or MMSD field personnel will have exclusive custody of any sample from the time of collection until the sample is deposited with the McLellan Lab. There are no custody or transmittal forms. Sampling site data is collected by the field technician and is included on the label for each sample, which is removed and saved for future reference after sample processing is complete. Samples will be delivered to the lab and filtering for microbiological plate counts and for environmental DNA (Appendices B-1 and B-2 respectively) will begin within 4 hours of collection. DNA filters will be stored at -80° C until further use.

Objective 2. Trained McLellan Lab personnel will collect/label field samples and keep them on ice while in the field. Field personnel will have exclusive custody of any sample from the time of collection until the sample is brought to the McLellan lab. Samples will be taken to the lab and filtering for microbiological plate counts and for environmental DNA (Appendices B-1 and B-2 respectively) will begin within 4 hours of collection. DNA filters will be stored at -80° C until further use.

Sample processing and handling matrix:

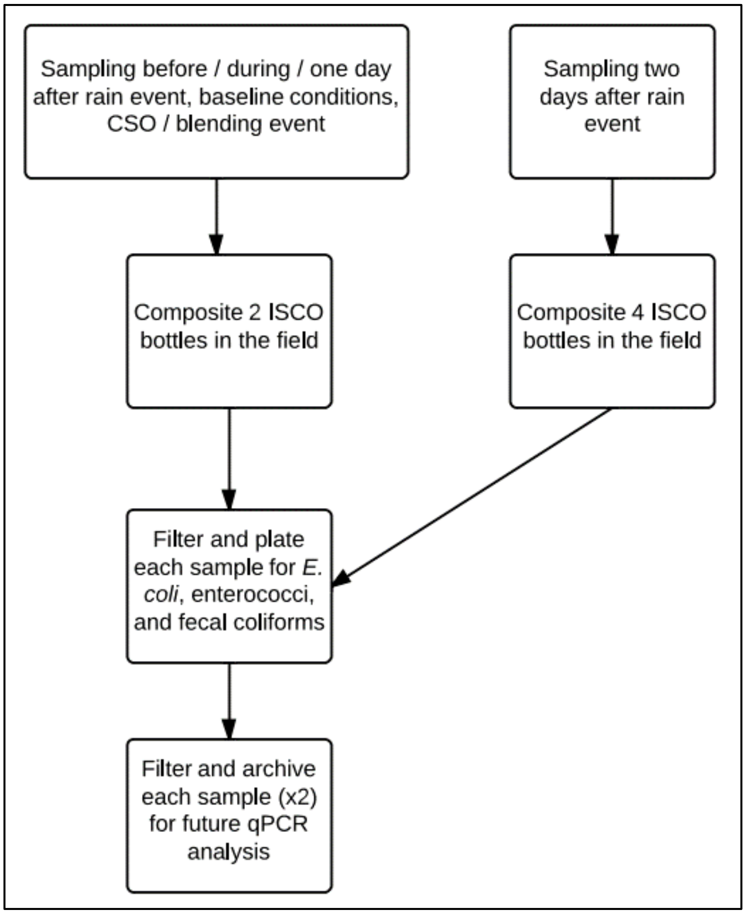

Objective 3. MMSD will keep all field samples on ice until delivery to the McLellan Lab. In the field, samples will be collected and labeled by trained MMSD personnel. MMSD field personnel will have exclusive custody of any sample from the time of collection until the sample cooler is picked-up by a member of the McLellan Lab. Samples will be delivered to the lab and filtering for microbiological plate counts and for environmental DNA (Appendices B-1 and B-2 respectively) will begin within 8 hours of collection. DNA filters will be stored at -80° C until further use.

Analytical Requirements

All laboratory procedures are detailed in Appendix B.

Data Acquisition Requirements (Non-direct Measurements)

As described previously, flow measurements will be obtained from USGS gauging stations. The procedures used to establish these stations are well established and considered authoritative. Documentation is readily available from USGS.

Quality Control Requirements

- All laboratory staff will adhere to current and generally accepted practices for safe handling, testing of samples, and chain of custody measures.
- Only clear, accurate, results will be used in calculations. Uncertain results, missing results (due to field limitations or laboratory incident) will not be used in calculations, but will be entered in lab books and databases as NA.
- Field blanks will be collected once per field collection (collect at a midpoint in site):
  1. Fill a clean container with blank Milli-Q water and place in the cooler to travel with the survey.
  2. Once on site, pour the blank water from the container into sample bottle in the same location where the field sample will be filled.
  3. In the lab, process blank in the same manner as other samples and notify field personnel if contamination is present.

- Laboratory blanks will be collected once a week:
  1. Fill a clean container with blank Milli-Q water.
  2. Clean filter set-up as would between site samples and run blank through filter apparatus.
  3. Plate for fecal indicators.
  4. If fecal contamination is present:
     1. Run 10% bleach through filter cups and manifold apparatus.
     2. Wash bleached filter cup and base with soap one time and thoroughly rinse three times.

Data Management

The McLellan Lab maintains a Microsoft Access database on a MySQL server that is backed up daily at UWM-School of Freshwater Sciences. Data is copied to the database from two sources:

1. From a project notebook
2. From a qPCR master spreadsheet

Data is entered into the database by trained McLellan lab personnel.

Database entries are checked for accuracy against the project notebook bi-weekly – the lab manager and a trained technician randomly check 10% of new entries.

The McLellan Lab manager and the Riverkeeper water quality specialist crosscheck Riverkeeper data throughout the season.

C. Assessment/Oversight

Assessments and Response Actions

The fieldwork and laboratory processes that are required for this project are commonly used by the collaborators involved in individual objectives. Therefore, all collaborators are able to assess compliance with fieldwork and laboratory expectations concerning their portion of the project. Any modifications or corrective actions taken in field collections or laboratory processing will be reported to the McLellan Lab manager and reported in the project notebook and may be added as a comment in the McLellan database.

Problems in ISCO automated sample collections will be apparent if the bottle rosette is incompletely filled or if individual bottles do not contain a full sample. If there is a problem the samplers will be reprogrammed, flushed and recalibrated.

Problems in sample collection process will be identified by fecal indicator contamination of field blanks. Blanks will be analyzed by qPCR for human-specific indicator bacteria. Any contamination between zero and three orders of magnitude lower than the samples collected during that sampling period will be considered negligible. Blanks with contamination greater than three orders of magnitude lower than the samples will result in samples from this sampling period to be discarded.

Monthly will take place to assess which samples will be included in follow-up qPCR assays. Samples will be evaluated based on plate counts of E. coli and/or enterococci. Samples will be chosen for qPCR analysis depending on the characteristics of each events' hydrograph. Although each sample represents a two hour interval, not every sample will be chosen for qPCR analysis. Depending on their location in the hydrograph of the event, every sample, every other sample, or every 4 samples will be analyzed to represent the entire event. Additional samples may be chosen when further information is needed to analyze patterns across the hydrograph.

Decision diagram for qPCR analysis of ISCO samples:

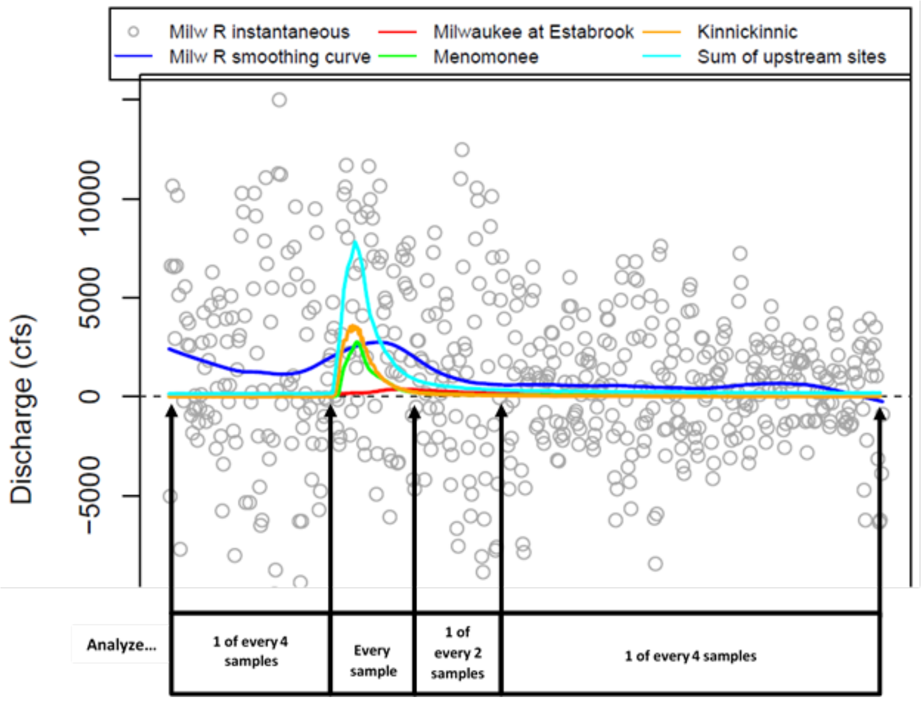

Reports to Management

Quarterly updates prepared by the McLellan Lab will be supplied to Stacy Hron, Milwaukee Estuary Area of Concern Coordinator at WDNR.

Quarterly Project team check in will be held with McLellan Lab, Riverkeeper and Stacy Hron.

In Quarter 7 of this project SFS Center for Water Policy will complete draft policy briefs for review by WDNR (OGL and TMDL staff).

In Quarter 8 of this project the McLellan Lab will update the comprehensive 2012-2015 Stormwater Report and distribute to MMSD and WDNR. The report will also be available for download off the McLellan Lab website.

D. Data Validation and Usability

Data Review, Validation, or Verification

All sample data will be reviewed by one or more McLellan Lab personnel:

1. Lab manager
2. Qualified QC technician
3. Qualified McLellan Lab graduate student directly involved in a project objective

Anomalous results found in database queries will be verified against projects notebook. If transcription errors are found, immediate corrections will be made.

Anomalous data will be reviewed by the McLellan Lab Director and a decision will be made on any actions to take if problems are found.

Reconciliation with Data Quality Objectives

Objective 1: Sewage contamination, as measured by human fecal indicator concentrations and loads, will be compared between outfalls to prioritize up-the-pipe investigations and remediation strategies, reaching our overall objective of reducing sewage pollution loads to the AOC. Average and frequency measurements will be used to develop a comprehensive map of illicit discharges in the lower Menomonee and Kinnickinnic River watersheds, which can be used as a resource by municipalities. Human-specific fecal indicators will be quantified in terminal outfalls and results mapped using GIS. Mapping will include drainage areas, elevations, sewer configuration and age of development. Pearsons correlation of these different variables to human indicator loads will be used to determine risk factors of failing infrastructure. We expect multiple variables will be indicative of a high likelihood of human indicator “hot spots”.

Objective 2: Samples across the hydrograph will be taken in the Milwaukee Estuary and the Milwaukee, Menomonee and Kinnickinnic Rivers in order to calculate event loads of sewage contamination entering the Estuary and begin to understand which rivers are the major contributors to the contamination. The R statistics suite of programs as well as various packages and functions developed for R will be used to build and analyze datasets. The dataRetrieval package (https://github.com/USGS-R/dataRetrieval) will be used to retrieve discharge data from the USGS gauging stations at or near each of the sampling locations by accessing the USGS National Water Information System (NWIS) available online. Discharge data is generally available in five minute intervals and will be downloaded for the time intervals which were sampled. The LoadInstantaneous function from the GSHydroTools package (https://github.com/USGS-R/GSHydroTools), developed by USGS, will be used to compute instantaneous loads of human indicator bacteria across the hydrograph for each event. Event loads will be calculated by taking the sum of each instantaneous load value calculated for the event. Rain events will be defined as periods of precipitation of at least 0.1 inches (2.25 cm) bounded by periods of at least six hours of dry weather before and after the period of precipitation. This time period will be used as a starting point for defining rain events, but may be slightly adjusted for each individual river. The USGS Environmental Data Discovery and Transformation (EnDDaT) online support tool will be used to build precipitation datasets for the sampling periods and the Rainmaker package will be used in R to define each rain event. Baseflow loads will be calculated over a 24 hour sampling period and CSO event loads will be calculated over the period of one to three days depending on the amount of untreated sewage discharged during the event and the duration and intensity of rainfall during the event. A regression analysis will be used to determine the strength of correlation between human-specific indicator concentrations and river discharge. This will allow us to determine

whether there is a clear relationship between sewage contamination and rainfall/runoff in the rivers and estuary, and whether this relationship changes between sites and weather conditions.

Objective 3: The Milwaukee River Basin is currently developing TMDLs for three rivers and the estuary. Our study site covers two of these watersheds; the Menomonee and Kinnickinnic Rivers. Our data will not be used in the TMDL development, but can inform the implementation stage. We will provide the comprehensive maps and a technical report highlighting the findings for each assessment reach in the Menomonee and Kinnickinnic Rivers. Historical data demonstrates the assessment points with the highest levels of fecal coliforms but the lowest concentrations of sewage. This project will provide information on the level of sewage concentration in each sample (in equivalents of untreated sewage influent; i.e. 1:100 dilution of influent, etc.). We have previously analyzed and reported levels of human markers human *Bacteroides* and human *Lachnospiraceae* in untreated sewage influent from Jones Island wastewater treatment plant and South Shore waste water treatment plant (Newton et al. 2011) and these levels have remained steady for the past three years in subsequent analysis. This allows us to calculate the amount of sewage markers in the river samples and report as equivalents of untreated sewage. Since multiple samples are taken on the sample day, assessment points can be compared under different conditions. The TMDL team (consultants Camp, Dresser and McGee and MMSD) will relate the sewage impact rankings (sites ordered from highest to lowest) and data to load reductions and consider this in prioritizing remediation actions. These results will be conveyed in a technical memo to Camp Dresser McGee (CDM) who is the consulting team on the TMDL project. We will also prepare a policy brief in collaboration with the Center for Water Policy reporting the comparative amount of sewage influent per unit volume (in this case 100 ml) of the river reaches analyzed in the Menomonee and Kinnickinnic Rivers. This policy brief will be disseminated to the entire TMDL team (MMSD, CDM, SEWRPC, and DNR) and will be shared with additional stakeholders. The TMDL team make the final decision on how to act upon this data.

APPENDIX A

SAMPLING PROTOCOL

A-1. Stormwater Sampling Protocol

Stormwater sampling at outfalls is conducted using the following protocol:

1. Label a clean 500 ml bottle with the following information.
   a. Sample location (Site ID)
   b. Date
   c. Initials of person collecting sample

2. Record information on log sheet (i.e. weather conditions, site description, GPS location, any unusual/helpful information).

3. Rinse bottle with sample water 2-3x with cap on bottle before collecting sample.

4. Capture free flowing water being discharged from the stormwater outfall being careful not to contaminate the rim of the bottle (i.e. soil or algae on rim).

5. Place bottles on ice or at 4ºC.

6. Deliver samples to the McLellan laboratory Monday through Thursday within 4-6 hours or ship overnight on ice.

A-2. ISCO Sampling Protocol

River and estuary samples will be collected using an automated ISCO sampler using the following protocol:

- Sampler Description:
  - A Teledyne ISCO 3700 full size portable sequential sampler is used for sampling.
  - ISCO sampler consists of a rosette of 24, one-liter polypropylene bottles in which samples are collected at regular intervals.
  - Includes a pump, which pumps water through a ¼-inch, flexible sample line into sample bottles. Sample line is automatically rinsed before each new sample is collected.
  - Controller on ISCO sampler allows for sample programming.

- Sampler Installation:
  - The ISCO samplers are installed within a sturdy, locked enclosure that is protected from damage due to tampering and environmental hazards (usually installed within a U.S. Geological Survey gaging station).
  - ISCO samplers are installed by extending the ¼-inch suction line from the sampler into the water body being sampled.
  - Sample line is secured using PVC pipe attached to a permanent structure, such as a river wall or concrete river bed.

- A Campbell® Scientific CR1000 datalogger and AirLink® LS300 Raven cell modem is installed to communicate with the ISCO, allowing for remote control and monitoring of the sampler.
- ISCO samplers are generally programmed to collect 250 mL every 15 minutes for up to 24 hours (each bottle represents a composite, one-hour sample).

- Field Collection:
  - ISCO samplers are turned on remotely using the Campbell® Scientific LoggerLink mobile application, which communicates with the datalogger.
  - Sampler is filled with ice to ensure sample preservation and ice is replaced once per day while the sampler is running.
    - When samples are collected, if ice is melted, the temperature of the melted ice water is recorded on a field sheet.
  - Water samples are collected in two-liter plastic Nalgene bottles, which are sanitized with dish soap after each use.
    - A small quantity of sample water from each sample bottle is poured into a Nalgene bottle, capped, shaken, and dumped to remove any soap residue from the bottle before the sample is collected (x3).
    - During and within 24 hours of an event, two ISCO sample bottles are combined into one, two-liter Nalgene bottle when the samples are collected (results in a two hour composite sample).
    - 24 hours after an event, four ISCO sample bottles are combined into one, two-liter Nalgene bottle when the samples are collected (results in a four hour composite sample).
    - Samples are immediately placed on ice and taken back to the lab for filtering.
  - One-liter ISCO sample bottles are rinsed with deionized water (x3) and replaced in the sampler after each sample is removed.
  - Bottle blanks are collected once per event at each site by collecting a sample of deionized water using the same procedure that a field sample would be collected.

A-3. MMSD Investigative Stormwater (Up-the-pipe) Sampling Protocol:

Investigative stormwater sampling will be conducted by MMSD using the following procedures:

Investigative Stormwater Sampling

**Procedure Number:**	MON-STORM-030	**Creation Date:**	February 28, 2014
**Prepared by:**	TMG	**Revision Date:**	
**Procedure Description:**	This procedure outlines the process to follow when conducting an investigative stormwater samples.		
**Procedure Responsibility:**	Monitoring Specialist, Monitoring Technician		
**Procedure Purpose:**	To collect samples and flow data from identified storm sewer manholes within areas of concern to analyze for pollutants in stormwater. Sampling events will include both wet and dry weather storm sewer sampling.		

Definitions

- **AOC –** Areas of Concern – Areas that have been preselected for sampling for various reasons (e.g. suspected cross-connections.)
- **COC –** Chain of Custody - Refers to the chronological documentation or "paper trail", showing the collection, custody, control, transfer, analysis, and disposition of samples collected in the field.
- **UWM SFS –** University of Wisconsin – Milwaukee, School of Freshwater Sciences
- **LIMS –** Laboratory Information Management System
- **In-line-**stormsewer sampling that occurs with the drainage area or manhole upstream from the outfall identified as of concern.
- **SW –** Stormwater
- **Buchner funnel** - A piece of laboratory equipment used in filtration. Removes larger particles from sample to be analyzed.
- **Area Velocity Sensor -** A submerged probe sensor capable of measuring both level and velocity. Level is measured using an internal differential pressure transducer housed within the AV sensor. Velocity is measured using ultrasonic sound waves emitted through the AV sensor, which are reflected off of particles and bubbles in the stream back towards the AV sensor.

Background

MMSD provides in-line SW sampling in support of District initiatives, municipalities within the District's Service Area, non-profit environmental groups, and academic institutions. A SW program will be completed annually identifying storm sewer locations of interest. Sampling events will include both wet and dry weather storm sewer sampling using automated samplers or grab sample techniques.

The sites selected for stormwater sampling are in response to MMSD and UWM SFS stormwater quality and bacteria results which identified areas of concern in need of additional in-line sampling. In-line sampling refers to stormwater sampling performed upstream of a location with an identified concern. Individual pollutants/parameters to be analyzed from each identified sampling location are generally decided upon in the spring of each year prior to commencing sampling.

Automated samplers are often utilized to perform Wet Weather stormwater sampling. Typically these are Sigma 900MAX Portable samplers with a base set up with eight discrete one-quart bottles. Samplers are generally initiated utilizing an area velocity probe and a minimum level set point.

SW Sampling often requires Confined Space Entry. To ensure compliance with the District's safety procedures, all confined entry sampling must be performed with a three-person crew. Refer to *District Policy, Field Monitoring Safety Manual* and *Monitoring Department Work Rules* for detailed information on required safety procedures for confined space entry.

Procedures

Installing SW Samplers

1. Prior to installing SW samplers, each location will be examined to ensure that sampling at the manhole is feasible. If the sampling at the identified SW sewer is not feasible an alternate sampling location will be sought.
2. Field Monitoring Department crews will initially install SW samplers/flow meters in April or early May of each year. Samples will be collected from April through October. Site-specific sampler/flow meter installation information will be recorded on the *Stormwater Meter Installation Form.*

3. Installation data will include coordinates, field activities, and observations which will be entered into the Site Maintenance Application.
4. Samplers will be programmed to initiate when the flow level reaches 0.2 feet. Each sampler contains 8 one-quart bottles. The first four bottles collect the first flush sample. The second flush sample is triggered two hours after the first samples were collected. If the second set of bottles is not filled, it typically means that there was not enough rainfall to generate a second flush.

Preparing for Wet Weather SW Sampling

1. After a rainfall, a Field Monitoring Supervisor will check the rain gauges in the AOC and notify the laboratory that there will be samples to receive. Wet Weather SW samples will be collected no more than once per week when the following conditions occur:
   - At least 0.1" of rainfall over sampling area; and
   - 72 hours of dry weather (<=0.1") prior to sampling;
2. On the morning of the sampling event, generate LIMS numbers for COC and bottle labels for the samples to be collected at each location.

Conducting Wet Weather SW Sampling

1. Field Monitoring crews will collect SW samples from each location when a first flush sample event has occurred.
   - Combine the samples collected in the first flush (set of first four) bottles into a one-gallon glass jar filtering them through a Buchner funnel.
   - If a second flush has been triggered, combine samples collected in the second flush bottles into a separate one-gallon glass jar also filtering them using a Buchner funnel.
2. After collecting samples, download stormwater flow data utilizing *Vision* from the samplers. Record appropriate information in *LIMS*, *Site Maintenance*, and the *Stormwater Wet Weather Route Sheet*.
3. After sample collection is completed, deliver SW samples to the laboratory utilizing proper COC procedures.
4. When wet weather sampling is complete, send *Vision* and *Site Maintenance* data to Field Data on FPS101 server. Then an email message will be sent to the Water Resource Program Supervisor and Systems Monitoring Database Analyst, noting that data was uploaded.
5. SW Samplers will be maintained (i.e. batteries changed, samplers checked) until the wet weather sampling at that location has been completed. Samplers will be removed at that time.

Preparing for Dry Weather SW Sampling

1. A Monitoring Supervisor will select times when dry weather SW sampling will be conducted and notify the laboratory of possible samples. Dry weather SW samples will be collected if possible when 72 hours of dry weather conditions (<=0.1" rainfall) have occurred, from locations where flow is observed. For each wet weather site, if possible, at least one dry weather background sample will be collected.
2. On the morning of the planned sampling event, generate LIMS numbers for COC and bottle labels for the samples to be collected at each location.

Conducting Dry Weather SW Sampling

1. Monitoring crews will collect dry weather storm sewer grab samples from each location if possible.
2. Fill out *Installation Forms* including locational coordinates, field activities, and observations. Enter

appropriate information into the Site Maintenance Application for future visits.

3. After collecting samples, record required information d in *LIMS*, *Site Maintenance*, and the *Dry Weather Stormwater Sampling Form.*
4. After sample collection is completed, deliver SW samples to the laboratory under proper COC procedures.
6. When a dry weather sampling event is complete, send *Site Maintenance* to Field Data on FPS101 server. Then send an email message to the Water Resource Program Supervisor and Systems Monitoring Database Analyst, informing them that you have uploaded Dry Weather SW Sampling Event data to the server.

A-4. MMSD Water Sample Collection Protocol

River water samples collected by MMSD field crews and delivered to the McLellan Lab will be collected using the following protocol:

A. Purpose

Describe procedures to be followed when collecting water samples aboard the WQR monitoring boats or the monitoring van. Samples are collected in one of five ways depending on professional judgment, site accessibility, and water depth. These five ways include:

- Sampling bucket
- Kemmerer
- Van Dorn
- ISCO pump
- Dipping the bottle in the water

B. Definitions

Bug Pole Sampling device used to hold sampling bottle for sterile collection of bacteria samples.

Equipment Blank A blank sample used to verify that sample collection equipment and the laboratory is not introducing contamination.

Field Blank A blank sample used to verify that field conditions and the laboratory are not introducing contamination.

Field Duplicate a replicate, sequential sample that is stored in a separate container, and analyzed independently.

FRM Freshwater Resources Monitoring.

FRMS Freshwater Resources Monitoring Supervisor.

Hold Time Amount of time a sample can be held after collection and before analysis.

ISCO Pump used to collect water from sites too shallow to sample with other instruments.

Kemmerer Sampler Device used to obtain water samples at different depths. The sampler is an open tube (oriented vertically) with two sealing ends that can be triggered at a specified depth to close. This sampler is used primarily for sites with multiple depths.

Messenger Metal weight threaded over a rope that is dropped to trigger a sampling device to close and capture a sample.

MSDS Material Safety Data Sheet.

PFD Personal Flotation Device.

Sampling Bucket Pail with a rope attached to it used to obtain water samples at shallow depths. This sampler is used primarily for sites with a single depth.

Trip Blank A blank sample used to verify that sample bottles, travel conditions, and the laboratory are not introducing contamination.

Van Dorn Device used to obtain water samples at shallow depths. The sampler is an open tube (oriented horizontally) with two sealing ends that can be triggered at a specified depth to close. This sampler is used primarily for sites with a single depth.

C. Responsibilities

All FRM staff

D. Equipment and Supplies

Multiprobe sonde, Sampling bucket/Kemmerer/Van Dorn, Coolers, Ice, Ice breaking weight, sample bottles with spares, computer, road salt alternative, ISCO, water proof boots, Nitrile gloves, Field Sheet.

E. Health and Safety Warnings

Some sample bottles are preserved with Sulfuric or Nitric acid prior to a survey. These acids can be painful when allowed to contact any part of the body, including inhalation of vapors. MSDS are located in L-150.

Loaded coolers can weigh in excess of 50lbs; while filled Kemmerers and Van Dorns weigh approximately 25lbs. Proper lifting form should be used when moving them (**especially when held away from the body**). Precautions need to be taken when exposed to traffic. Multiple sites have relatively high speed limits and so warning lights on the sampling vans need to be used to alert drivers to your presence.

Due to the nature of sampling water, ice can form at the sampling sites creating hazardous conditions. Take necessary measures to avoid slipping and never walk out on an ice shelf that may collapse. If the sampler gets splashed or falls into the water and is at risk for hypothermia, be sure to get to a warm place and change into dry clothes or dry blanket if available.

Bridges can be dangerous due to their height. Losing your footing and falling off the bridge is a risk where sand and other debris line the sides. Also, be cautious that the sampling equipment does not snag any floating material that could potentially pull you over.

On occasion, sampling needs to be done during rain events. These events can produce lightning and if any is spotted, samplers should retreat off of bridges, off any other exposed area, go inside Pelagos and if possible, find safe harbor. PFDs should be in use when underway on any of the FRM sampling vessels.

Never allow a battery or ISCO pump to be submerged in the water. Electrical current can be transferred to you through the water.

F. Procedures

Collecting Samples (Boat and Van Surveys)

Before collecting a water sample, record the sample date on each sample bottle, take depth reading on multiple depth sites using available equipment (a multiprobe sonde can be used on van sites while the depth gauge is typically used when on board a sampling vessel), and calculate sample depths. Multiple depth sites can require up to three samples, 1) one meter depth, 2) one meter off the bottom, and 3) midpoint. For example, if depth is 10 meters, samples will be taken at 1 meter, 5 meters, and 9 meters. If a site is not deep enough to require three depths, it may be limited to a surface and bottom or just a surface. Record any useful notes on the field data sheet. Useful notes commonly include weather details, unusual conditions at the sampling site, possible sources of contamination, reasons for missing a site, to record sampling time of a field blank or site that does not require a multiprobe sonde measurement.

When collecting a water sample, use good field practices as outlined in Appendix A.

1. Sampling bucket
   a. Dip sample bucket into water. Allow the sampling bucket to partially fill with water and after rinsing, discard away from where the actual sample will be taken.
   b. Dip the sampling bucket again to collect sample and retrieve from water.
   c. Allow sample water to flow from sampling bucket into bottles (do not overfill preserved bottles). Cover immediately with correct caps once bottles are full.
2. Kemmerer
   a. Open the Kemmerer by firmly pulling the end seals apart until they lock open.
   b. Lower the Kemmerer to the desired depth either by hand or crane if available.
   c. Move the open Kemmerer through the water letting it rinse off the interior and exterior of the device. Keep the rope taught and drop the messenger down. The messenger will trigger the end seals to close once it makes contact with the Kemmerer.
   d. Retrieve the Kemmerer from the water.
   e. Allow sample water to flow from Kemmerer into bottles (do not overfill preserved bottles). Cover immediately with correct caps once bottles are full.
3. Van Dorn
   a. The Van Dorn has a wire on each end seal that should be pulled up to the trip assembly of the tube. There are two metal pegs in the trip assembly that the loop of each wire should fit over in order to hold the device open.
   b. Lower the Van Dorn to the desired depth either by hand or crane if available.
   c. Move the open Van Dorn through the water letting it rinse off the interior and exterior of the device. Keep the rope taught and drop the messenger down. The messenger will trigger the end sealsto close once it makes contact with the Van Dorn.
   d. Retrieve the Van Dorn from the water
   e. Allow sample water to flow from Van Dorn into bottles (do not overfill preserved bottles). Cover immediately with correct caps once bottles are full.
4. The ISCO pump
   a. Attach a charged, 12 volt battery to the ISCO in the battery holder.
   b. Plug cable from battery into “12 VDC” outlet.
   c. Pump speed may be adjusted using the “Pump Speed” knob
   d. Use the power knob to control the direction of pumping water. “Stop” shuts off power to the pump while “Run” starts the pump. Operating the pump in forward or reverse depends on how the tubing is oriented and may require you to change to the other “Run”.
   e. For help replacing the tubing inside the pump, please reference the ISCO Manual.
   f. Place tube end with the filter into sampling water in a way to avoid sediment from clogging the tubing. Run pump for at least 1 minute to flush out any residual water or debris from last site.
   g. Allow sample water to flow from outlet tube of the ISCO pump into bottles (do not overfill preserved bottles). Cover immediately with correct caps once bottles are full. Do not allow the outlet tube to touch the interior of the sample bottle.
5. Directly dipping the bottles in the water
   a. This method does not require rinsing.

b. Submerge bottles into water (do not overfill preserved bottles). Cover immediately with correct caps once bottles are full. Be careful to not accidently stir up the sediment or scrape any debris into the bottle.
    i. Keep hands away from or downstream of the opening to avoid sample contamination.

6. Using a bug pole (directly dipping the bottle may be used as a sampling technique if the site is accessible by foot. See step 5).
    a. Place the bottle in the harness and submerge it approximately 6-8 inches under the water when possible to grab a sample (see "References" section below for details about methods used). Empty some of the sample out of the bottle to leave one to one-half inch of air or "headspace" in the bottle.
    b. Do not contaminate the bottle cap of the fecal coliform/E. coli sample by allowing it to come into contact with any other surface.
    c. Place sample bottles in coolers on ice. Fecal Coliform bottles must be stored upright. The other bottles, if properly sealed may be stored on their sides.
7. If a site requires more sample than one of the sampling devices can hold, using multiple pulls fill the bottles as uniformly as possible.
    a. Take one pull of water and fill up every bottle to about 50% full.
    b. Collect a second pull of water from the same depth and location as the first pull and fill every bottle to full creating homogenized, composite samples.
8. When sampling at a site is complete, move filled bottles out of the sunlight, into coolers with ice. The sampling equipment should be closed and laid down on its side. This prevents debris from getting inside the equipment, keeps the nozzle from being contaminated by touching the ground and prevents damage to the sampling equipment and to the boat/van.
9. During colder months, ice may develop over sampling sites. If possible, use the ice breaking weight to attempt to break a hole large enough to sample from.
    a. After finishing sampling, an adequate amount of environmentally friendly deicing material should be placed on any spilled water to avoid creating a slipping hazard for the public on any bridges or walkways.

G. Quality Assurance and Quality Control

a. Collect a sufficient number of quality-control samples.

1. Field Duplicates
    i. Collect a duplicate for every variable except fecal coliform, E. coli, total solids, suspended solids, and volatile suspended solids every 20 sites.
    ii. Collect a duplicate for total solids, suspended solids, and volatile suspended solids every 10 sites.
2. Trip Blanks
    a. Fill trip blanks with RODI water immediately prior to the survey and place in a cooler to travel along with the survey.
    b. Every variable except fecal coliform and *E. coli* have a trip blank.
    c. Take a separate trip blank for each day of a multi-day survey.
    d. CP surveys require a trip blank on the van portion of as well as a separate trip blank on the boat portion for the day.
3. Field blanks (may be required on a case by case basis for special projects).
    a. Fill a clean container with blank RODI water and place in the cooler to travel with the survey.

b. Once you have arrived at the site. Pour the blank water from the container into sample bottles in the same location where the field samples will be filled.

4. Equipment blanks may be required on a case by case basis for special projects and QA surveys.
   a. Sampling equipment may need to be cleaned before the blank is collected. See WQR-OPS-045 for QA cleaning procedures.
   b. An equipment blank sample is collected sometime during the survey for both the van and boat portions of a survey.
   c. Pour deionized water through the sampler and collect it in the appropriate sample bottles. This sample is collected only once on both the van and boat portions.

H. References

ASTM Standard D888 – 05, “Standard Test Methods for Dissolved Oxygen in Water," ASTM International, West Conshohocken, PA, 2005, DOI: 10.1520/D0888-05

Standard Methods for the Examination of Water and Wastewater, Method 4500-O G, 4500-H+B, 20th ed., 1998. APHA, AWWA, WEF. Washington, DC.

National Field Manual of the Collection of Water-Quality Data, United States Geological Survey. Techniques of Water-Resources Investigations. 2006.

Wisconsin Administrative Code, Wisconsin Department of Natural Resources , NR 149, “Laboratory Certification and Registration”, Register November 2009;

Wisconsin Administrative Code, Wisconsin Department of Natural Resources , NR 218, “Method and Manner of Sampling”, Register August 1997; NR 219, “Analytical Test Methods and Procedures”, Register April 2010.

YSI 6-Series Multiparameter Water Quality Sondes User's Manual. Hach Environmental, Loveland, CO. November 2010

I. Appendices

Appendix A: Good Field Practices

- Be aware of and record potential sources of contamination at each field site.
- Wear nitrile disposable, powderless gloves:
  - Change gloves before each site during sample collection.
  - Avoid hand contact with contaminating surfaces (such as non-essential equipment, coins, food).
  - Gloved as well as ungloved hands must not contact the water sample or the interior of the bottle and bottle cap.
- Use equipment constructed of materials that are relatively inert with respect to the analytes of interest.
  - Field rinse equipment.
  - Hold sampling device at an angle so as to not allow water on the outside of the device to drip down into the bottles.
  - Do not touch or plug the nozzle of any sampling equipment.
- Use correct sample-handling procedures:
  - Minimize the number of sample-handling steps.
- Keep bottle caps organized to avoid cross contamination of preserved bottles with non-preserved bottles.

  - Place caps with the interior in a way to avoid contamination when filling bottles from splashed water and windblown debris.
- Minimize the amount of time that open bottles are exposed to the atmosphere and precipitation.
- Collect samples an adequate distance from sources of contamination (e.g. exhaust from van or sample boat).
- Follow a prescribed order for collecting samples.

APPENDIX B

LABORATORY PROTOCOL

B-1. Bacterial Enumeration Protocol

Outfall, river, and harbor samples received by the McLellan Lab will be filtered for the enumeration of *E. coli*, enterococcus, and total fecal coliforms using the following protocol:

1. Environmental water samples (EWS) are filtered through a 0.45 μm pore size 47mm nitrocellulose filter (Millipore, Bedford, MA)
2. Volumes filtered varied according to the site location and expected contamination level.
3. Place on the appropriate culture media.
4. Incubation temperature depends on the culture media and organism.

Media

Organism	Media
E coli	**Mod-mTech**
E. coli	**mTech**
E. coli **total coliform**	**MI**
**enterococcus**	**MEI**
**Fecal coliforms**	**mFC**

Cell Count Filtration

Site	Filter Volume Normal	Filter Volume Rain Event
**USGS/MMSD Rivers**	**10ml/100ml**	
**USGS/GLRI Rivers**	**10ml/100ml**	
**MKE/MMSD/SFS Rivers**	**1ml/10ml**	
**Stormwater**	**Dilute 10X plate 1ml**	
**Harbor/Lake**	**100ml**	
**ISCO**	**10ml/100ml**	
**Beaches**	**100ml**	**10ml, 100ml**
**Sand Elution**	**10ml**	
**Sand Elution Northern Beaches**	**100ml**	

Volumes are estimates
Samples may have to be re-plated at higher or lower volumes

B-2. DNA Filtering Protocols

Outfall, river, and harbor samples received by the McLellan Lab will be filtered for the collection of DNA, using the following protocol:

- Sterile 500 ml collection bottles are provided (if needed)

- Assemble vacuum pump filtration station – 6-cup filter manifold, magnetic filter funnels (top & bottom piece), pump (standard plug in), carboy for water collection, tubing connecting carboy-pump-filter manifold
- Lab gloves (latex, nitrile, etc.) should be worn while handling filter funnels and filters – to prevent transfer of bacteria from hands. If not always possible, keep hands etc. (not sure what etc. is, but best to keep it out too) out of inside of filter funnels and sample bottles.
- Forceps should be kept clean between filter transfer to manifold. Ethanol on a paper towel can be used to wipe them down, or if not available, a simple wipe down with a paper towel to remove any water/debris will suffice. Also attempt to keep forceps out of sample water and on a clean surface as much as possible.

1. After water collection – either distribute water to "clean" sample bottles or distribute directly from sampling apparatus. Keep sample water in cool & relatively dark place if they are not processed shortly after collection. Samples may sit in filter area for 0.5-1 hr during processing – if longer it would be best to keep cool (if possible).
2. Assemble filter funnels. Filter funnels have a top and bottom connected by magnetic force.
   a. Place bottom of filter funnel into filter manifold port – so that the rubber stopper is secure (doesn't need to be jammed on, but should be pressed down lightly to secure).
   b. With clean forceps, take 0.22 μm nitrocellulose filter (47 mm, Millipore, Bedford, MA) out of filter package – are individually wrapped. Blue disk, is for protection. Filter is white (hatched side is up). It is preferable to grab the filter by the edge & not the middle to minimize forceps contact. Filters will tear – if that happens discard & get a new one.
   c. Place filter hatched side up on the center black circle of the filter funnel bottom. The filter should be centered within the raised edges of the funnel. Take care that it is relatively centered – if not the water will be able to go around the filter. It does not need to be perfectly centered.
   d. Once in place, attach filter funnel top – will snap down via its magnetic force. Once down, it is in place – make sure it is not ajar.
3. Add 200 ml of water sample to filter funnel
   a. 200 ml for **rivers or stormwater** samples (x2)
   b. 200 ml for **ISCO** samples (x4)
4. Turn on vacuum pump (switch).
5. Turn filter port switch from horizontal to vertical – opens vacuum suction to port.
6. Check pressure on pump – should be in the 5-6 range. This can be adjusted via a circular disc on the right side of the pump, below the hose connector. The disk is connected via a vertical post and is horizontal to the tabletop. Small rotations in this disk will greatly adjust pressure, so move slowly and allow a few seconds for adjustment to take place. Usually once set it won't have to be moved again.
7. Wait for water to completely filter. Once finished, remove filter funnel top with vacuum still running. This allows in water that was stuck in along the edges to be sucked down.
8. Close filter port switch (move to horizontal position). Shuts off suction to only the port that has finished filtering.
9. With clean forceps fold filter in half 4 times (also can be folded in half and rolled with forceps (may take a couple of tries to figure out own personal best method).
10. Place filter into a 2 ml O-ring, screw capped microcentrifuge tube. Tube has McLellan lab tracking number (FT#) on it.
11. Place filter tube into cold storage box and freeze at -80°C.
12. Record sample ID on sampling sheet in conjunction with proper FT# & any associated notes.

13. Before reusing the filter funnels, reconnect the two halves & rinse with sterile (preferable) or "tap" water – vacuum pull this water through filter funnel, with no filter present. This will "clean" the filter funnel for the next sample. Also if extra water is available it is always a good idea to rinse the filter funnel with a little sample water, before filtering that sample.

2x filters per station/site/depth is preferable. If filtering sample duplicates (or more), there is no need to rinse filter funnels between replicate sample filtering.

Sample bottles can be re-used. Rinse (3x) with sterile (or tap) water before adding the next sample to the bottle.

Blanks for QA (sterile water) may be run 3x throughout the trip.

B-3. <u>DNA Isolation Protocol</u>

Outfall, river, and harbor samples received by the McLellan Lab will be filtered (as described above) and DNA will be isolated for qPCR analysis using the following protocol:

Environmental water samples

MP Biomedicals FastDNA Spin Kit For Soil.

1. Frozen filters are broken up into small fragments with a metal spatula.
2. DNA is extracted using the MP Biomedicals FastDNA Spin Kit For Soil (Solon, OH)
    a. Glass beads from kit added to pulverized filters
    b. DNA extraction proceeded in filter tube
3. Cells are mechanically lysed using the MiniBeadBeater-8 Cell Disruptor (BioSpec Products, Bartlesville, OK)
    a. homogenation setting for 1.0 minute at room temperature.
4. Follow Spin Kit extraction procedure
5. DNA is eluted in 150 μl of sterile, distilled water.
6. Samples stored in low retention tubes at -20°C
7. DNA concentration is determined using the NanoDrop ND-1000 Spectrophotometer (NanoDrop Technologies, Wilmington, DE).
8. Samples are stored at -20°C.

B-4. <u>Quantitative Polymerase Chain Reaction Protocols</u>

Outfall, river, and harbor samples received by the McLellan Lab will be analyzed for human-specific indicators using quantitative polymerase chain reaction (qPCR) protocols outlined below:

BASIC REAL TIME PCR CONDITIONS

25 μl qPCR reactions:

1. 2X Taqman® Gene Expression Master Mix Kit (Applied Biosystem; Foster City, CA)
2. Primers, probes, DNA Targets (Table 2)
    a. Final concentration 1.0 μM primers and 80nM probe
    b. Environmental Water Sample DNA
        i. Samples were diluted as outlined below;
        ii. 5ul of each DNA sample is added to each reaction

Environmental sample DNA preparation for real time PCR.

Genetic Marker	Sites	Dilution	DNA Extraction Method
All Markers	All	1:1	Spin Kit
All Markers	Beaches	None	Spin Kit
All Markers	Beaches/Drinking water	None	Crude Extract

c. Standards
   i. Range of quantification for plasmid and genomic standards
      1. 1.5E6 to 1.5E1 copies
   ii. New standard preparations
      1. prepare in triplicate
   iii. Subsequent standards from same preparation
      a. run two concentration standards e.g. 15000; 1500
   iv. Run two previous EWS in duplicate; testers

d. Inhibitor plasmid 100 copies, if required

3. PCR Conditions:
   a. 5 minutes at 50°C to activate the uracil-N-glycosylase (UNG),
   b. 10 minute incubation at 95°C to inactivate the UNG and activate the Taq polymerase;
   c. 40 cycles at 95°C for 15 seconds and 1 minute 60°C

Real Time PCR Primers and probes

TARGET	PRIMER/PROBE	SEQUENCE	AMPLICON SIZE	REFERENCE
***Bacteroides* Human**<br>**16s rDNA**<br>**4-7 genomic copies**	**HF183F**<br>**HF241R**<br>**HF193p**	5′ATCATGAGTTCACATGTCCG3'<br>5'CGTTACCCCGCCTACTATCTAATG3'<br>5'[6FAM]-TCCGGTAGACGATGGGGATGCGTT [MGB-NFQ] 3'	86bp	Bernhard and Field 2000<br>Kildare et al 2007
E. coli<br>***β-glucuronidase***<br>**1 genomic copy**	**uidA1663F**<br>**uidA1790R**<br>**uidA1729p**	5'GCGACCTCGCAAGGCATA3'<br>5'GATTCATTGTTTGCCTCCCTGCTGCG3'<br>5'[6FAM]TGCAGCAGAAAAGCCGCCGACTTCGG [MGB-NFQ] 3'	127bp	Li et al 2006
Enterococcus faecalis<br>**23s rDNA**<br>**4 genomic copies**	**Entero1F**<br>**Entero2R**<br>**Enterop**	5'AGAAATTCCAAACGAACTTG3'<br>5'CAGTGCTCTACCTCCATCATT3'<br>5'[6FAM]TGGTTCTCTCCGAAATAGCTTTAGGGCTA-[MGB-NFQ] 3'	92bp	Ludwig and Schleifer 2000<br>Haugland et al 2005
**Clostridiales**<br>**Group 2 assay**<br>**Lachno2**	**CcocR_RC**<br>**1032ClosR_2**<br>**989ClosP_2**	5'TTCGCAAGAATGAAACTCAAAG3'<br>5'AAGGAAAGATCCGGTTAAGGATC3'<br>5'[6FAM]ACCAAGTCTTGACATCCG[MGB-NFQ] 3' **Lachno2p**	137bp	Newton et al 2011
**Ruminant-Specific Bacteroidetes**	**RumBacR_f**<br>**RumBacR_r**<br>**RumBacR_p**	5'GCGTATCCAACCTTCCCG3'<br>5'CATCCCCATCCGTTACCG3'<br>5'[6FAM]CTTCCGAAAGGGAGATT-[MGB-NFQ]3'	118bp	Reischer et al 2006

DNA Name	Target Name	DNA Target	Vector	Host	Insert Size	Total Plasmid Size	Real Time Amplicon Size	Restriction Site	Antibiotic Resistance
**pSEW16**	*Bacteroides* human	16s rDNA	pCR2.1[a]	TOP10[a]	695 bp	4626 bp	80 bp	HindIII	Ampicillin[b]
**puidA1.8**	*E. coli*	β-glucuronidase	pCR2.1[a]	TOP10[a]	1773 bp	5704 bp	127 bp	HindIII	Ampicillin[b]
Enterococcus faecalis	Enterococcus	23s rDNA	Genomic				92 bp		
**Clostridiales Group 2 Clone:99H07**	Lachnospiraceae Human marker	16s rDNA	pCR2.1[a]	TOP10[a]	1015 bp	4946 bp	137bp	HindIII	Ampicillin[b]
**pRUM-G12**	Ruminant	16s rDNA	pCR2.1[a]	TOP10[a]	118 bp	4049 bp	118 bp	HindIII	Ampicillin[b]

Description of Plasmid and Genomic DNA Standards for Calibration Curves

[a] Invitrogen

[b] Ampicillin final concentration 100ug/ml

APPENDIX C

DATA ANALYSIS PROTOCOL

C-1. GIS Database and Mapping

GIS mapping and maintenance of GIS databases will be conducted by the following protocols:

1) **In collaboration with MMSD and Milwaukee Riverkeeper, we will maintain and improve an existing GIS database to continue to map all outfalls and manage collected data.**

   a. Milwaukee Riverkeeper has maintained a GIS database of stormwater outfall sampling locations since 2009. The outfalls were identified using existing municipal and MMSD information as well as through field survey by Milwaukee Riverkeeper. All outfalls, including municipal and MMSD outfalls, were field verified and GPS'd by Milwaukee Riverkeeper, using a Garmin GPSmap 60Cx handheld GPS unit. The coordinate system used by the GPS unit is WGS 84**.** We collect the GPS points with sub-10 meter which we feel is sufficient; we don't need post-collection rectification or correlation with known landmarks as the outfalls themselves are stationary and we can find them easily with this unit.

   b. GIS data for municipal and MMSD sewer infrastructure was shared with Milwaukee Riverkeeper through a user agreement with MMSD indicating that the information was to be used for internal project use only and should not be distributed. The data was to be used with the understanding that the layers are continually updated on an as-needed basis, so accuracy of the data is based on the date the data layers were created. Since GIS data originating from MMSD and the various municipalities is used solely as reference data and is not used for spatial analysis, having the most current data is not pertinent to Milwaukee Riverkeeper's GIS database.

   c. MMSD has provided Milwaukee Riverkeeper with most of the GIS layers we use for correlation and MMSD layers are projected in the NAD 1927 State Plane Wisconsin South FIPS 4803" coordinate system. Thus, our database stores information using the same coordinate system. ArcGIS automatically re-projects the GPS unit datum (WGS 84) into the NAD 1927 State Plane Wisconsin South FIPS 4803 coordinate system, so everything lines up nicely. In fact, ArcGIS re-projection capability aligns any new layers seamlessly with the GIS database. Again, at this time, our use of additional GIS layers is for correlation and reference, not precise analysis or computation.

   d. The GIS database contains the coordinates of stormwater outfalls, lab results from Sandra McClellan's lab at the Great Lakes Water Institute, and field notes gathered during stormwater sampling events by Milwaukee Riverkeeper.

   e. Lab results are first entered into a Microsoft Access database at the Great Lakes Water Institute. Lab employees download lab results from the database into an Excel

spreadsheet and share this information with Milwaukee Riverkeeper. Milwaukee Riverkeeper then manually enters the lab results into the GIS database.

f. Data quality and assurance of the lab results are ensured through rigorous review of all prior lab results per each outfall every time a set of new lab results are entered into the GIS database.

g. Any discrepancies in data relate mainly to outfalls not recorded by the municipality or MMSD, and are not due to coordinate system issues. The municipalities and MMSD are only required to report on outfalls of a certain size, so they often don't include smaller outfalls which Milwaukee Riverkeeper has identified.

2) The sample design is expected to provide comprehensive coverage of stormwater discharges to areas of the river hypothesized to be "high risk" as identified by surface water quality data for fecal coliform. Each outfall is monitored multiple times to assess flow during dry weather and to collect samples if possible; outfalls are also sampled during three separate wet weather events. These sample results are correlated with other GIS layers such as: municipal storm, municipal sanitary, MMSD infrastructure, current and pre-1930 combined sewer area boundaries, hydrology, topography, soil conditions, drainage, infiltration, urban growth, transportation and aerial photos. These layers were acquired by SEWRPC.

a. As lab results are compiled for each outfall (3 samples for outfalls flowing during rain events and 1 sample for outfalls flowing during dry weather), outfalls with multiple high levels of bacterial contamination come to light. GIS analysis can pinpoint concentrated areas of consistent high bacteria levels. By using additional related data layers, GIS analysis can enlighten possible sources and causes of this contamination as well as provide strategies for fixing the problems.

b. Geographic information relating to location of municipal storm, sanitary and combined sewer systems, along with MMSD infrastructure, allows insight into possible infrastructure failures and areas of cross-contamination between the sanitary, storm and combined sewer systems.

c. The Combined Sewer Areas from pre-1930 and the historical urban growth GIS layers identify areas of aging infrastructure as well as possible locations for incorrect or incomplete separation of historic combined sewers to separated sewer systems.

d. Hydrology, topography, soil conditions, drainage and infiltration layers all have the potential to add additional insight into bacterial contamination sources.

APPENDIX D

FIELD SHEETS

ISCO SAMPLE COLLECTION FIELD SHEETS

Site: ____ **Collection Date**: ____________

Field Crew: ____

Weather Conditions (circle one): Baseflow Light Rain Heavy Rain Blending CSO

Ice Melted? (circle one): No Yes (If yes) Melt water temperature: _______

First ISCO Sample Collected:

Time: ____________ Date: ___________
1 of 4, Bottle 1

Last ISCO Sample Collected:

Time: ____________ Date: __________
_______ of 4, Bottle_______

Site Checklist:

- ☐ Samples iced?
- ☐ Sampler on Bottle 1?
- ☐ Sampler reset or turned off in LoggerLink?
- ☐ Sampler Reset? Display reads "1 OF 4, BOTTLE 1 AFTER 1 PULSES"
- ☐ No kinks in the sample line?

Number of ISCO bottles (1-24): __________ Number of bottles composited (1-4): __________

Number of samples returned to lab: __________ Sample FT #'s: ____________ - ____________

Bottle blank collected? (circle one): Yes No (if yes) Blank sample FT#: _________

Additional field notes:

Last Update: 7/2/2014

References

Bernhard, A. E. and Katharine G. Field, 2000. A PCR Assay to Discriminate Human and Ruminant Feces on the Basis of Host Differences in *Bacteroides-Prevotella* Genes Encoding 16S rRNA. Appl Environ Microb 66: 4571-4574.

Dick, Linda K. and Katharine G. Field, 2004. Rapid Estimation of Numbers of Fecal *Bacteroidetes* by Use of a Quantitative PCR Assay for 16S rRNA Genes. Appl Environ Microb 70: 5695-5697.

Haugland, R. A., S. C. Siefring, L.J. Wymer, K. P. Brenner and A. P. Dufour, 2005. Comparison of *Enterococcus* Measurements in Freshwater at two Recreational Beaches by Quantitative Polymerase Chain Reaction and Membrane Filter Culture Analysis. Water Research 39: 559-568.

Kildare, Beverly J., Christian M. Leutenegger, Belinda S. McSwain, Dustin G. Bambic, Veronica B. Rajal, Stefan Wuertz,. 2007. 16S rRNA-based Assays for Quantitative Detection of Universal, Human-, Cow-, and Dog-specific Fecal *Bacteroidales*: A Bayesian Approach. Water Res 41: 3701-3715.

Li, J., McLellan, S. L., and Ogawa, S., 2006. Accumulation and Fate of Green Fluorescent Labeled *Escherichia coli* in Laboratory-scale Drinking Water Biofilters. Water Research 40: 3023-3028.

Lu, J., J. Santo Domingo, R. Lamendella, T. Edge, and S. Hill. 2008. Phylogenetic Diversity and Molecular Detection of Bacteria in Gull Feces. Appl Environ Microb 74, 13: 3969-3976

Ludwig, W. and K.-H. Schleifer 2000. How Quantitative is Quantitative PCR with Respect to Cell Counts? Sys. Appl Microbiol 23: 556-562.

McLellan, S. L. and D.K. Dila. 2014. Greater Milwaukee Watersheds Stormwater Report – March 19, 2008 through October 23, 2012. Report.

Newton, Ryan, Vandewalle, Jessica L, Borchardt, Mark A, Gorelick, Marc H and McLellan, Sandra L, 2011. *Lachnospiraceae* and *Bacteroidales* Alternative Fecal Indicatiors Reveal Chronic Human Sewage Contamination in an Urban Harbor. Appl Environ Microbiol 77: 6972-6981.

Reischer, G., D.C. Kasper, R. Steinborn, R.L. Mach, and A.H. Farnleitner. 2006. Quantitative PCR Method for Sensitive Detection of Ruminant Pollution in Freshwater and Evaluation of This Method in Alpine Karstic Regions. Appl Environ Microb 72, 8: 5610-5614

Shanks, Orin C., Emina Atikovic, A. Denene Blackwood, Jingrang Lu, Rachael T. Noble, Jorge Santo Domingo, Shawn Seifring, Mano Sivaganesan, and Richard A. Haugland, 2008. Quantitative PCR Detection of Enumeration of Genetic Markers of Bovine Fecal Pollution. Appl Environ Microb 74: 745-752.

Shanks, O.C., K. White, C.A. Kelty, S. Hayes, M. Sivaganesan, M. Jenkins, M. Varma, and R.A. Haugland. 2010. Performance Assessment PCR-Based Assays Targeting Bacteroidales Genetic Markers of Bovine Fecal Pollution. Appl Environ Microb 76, 5: 1359-1366

Siefring, S. M. Varma, E. Atikovic, L. Wymer and R. A. Haugland, 2008. Improved Real-Time PCR Assays for the Detection of Fecal Indicator Bacteria in Surface Waters with Different Instrument and Reagent Systems. J Water and Health 6: 225-237.

Sivaganesan, Mano, Shawn Seifring, Manju Varma, Richard A. Haugland and Orin Shanks, 2008. A Bayesian Method for Calculating Real-Time Quantitative PCR Calibration Curves Using Absolute Plasmid DNA Standards. BMC Bioinformatics 9: 120.

VandeWalle, J.L., Goetz, G.W., Huse, S.M., Morrison, H.G., Sogin, M.L., Hoffman, R.G., Yan, K. and Mclellan, S.L., 2012. Acinetobacter, Aeromonas and Trichococcus Populations Dominate the Microbial Community within Urban Sewer Infrastructure. Environ. Microbiol. 14: 2538-2552.

Zhang, Y., Zhang, D., Wenquan, L., Chen, J., Peng, Y., and Cao, W. 2003. A Novel Real-time Quantitative PCR Method Using Attached Universal Template Probe. Nucleic Acid Res 31: e123.
