# Supplementary material for: High levels of sewage contamination released from urban areas after storm events: A quantitative survey with sewage specific bacterial indicators
Source: PLoS Med. 2018 Jul 24;15(7):e1002614. doi: 10.1371/journal.pmed.1002614 (PMC6057621; doi:10.1371/journal.pmed.1002614)
Supplement: S1 Table — (PDF) [file pmed.1002614.s003.pdf]

S1 Table. Watershed characteristics of sampling sites in Milwaukee, Wisconsin. Base data from the National Land Cover Database (2011) Land Cover and Percent Developed Imperviousness datasets (Jin et al., 2013; Xian et al., 2011).

Sampling Location Name	Monitoring Location	USGS Site ID	Latitude, Longitude	Drainage Area (km^2)	Percent Impervious Surfaces	Percent Urban	Percent Agriculture	Percent Natural Areas[a]
Kinnickinnic (KK) River	Kinnickinnic River at 11th Street	04087159	42°59'51", -87°55'35"	51	52	99	0	1
Menomonee (MN) River	Menomonee River at 16th Street	04087142	43°02'02", -87°56'03"	349	28	69	17	14
Milwaukee (MKE) River	Milwaukee River at Cherry Street	n/a	43°02'54", -87°54'41"	1,781	7	19.5	50.5	30
Milwaukee estuary	Milwaukee River at Mouth	04087170	43°01'28", -87°53'54"	2,215	12	30	43	27

[a] Forests, grasslands, wetlands, and open water are classified as natural areas
