# Supplementary material for: High levels of sewage contamination released from urban areas after storm events: A quantitative survey with sewage specific bacterial indicators
Source: PLoS Med. 2018 Jul 24;15(7):e1002614. doi: 10.1371/journal.pmed.1002614 (PMC6057621; doi:10.1371/journal.pmed.1002614)
Supplement: S2 Table — USGS, US Geological Survey. (PDF) [file pmed.1002614.s004.pdf]

S2 Table. USGS flow-monitoring station used to retrieve continuous river discharge data. USGS, US Geological Survey

Monitoring Location	USGS Site Identification Number	Latitude	Longitude
Kinnickinnic River at 11th Street	04087159	42°59'51"	-87°55'35"
Menomonee River at Wauwatosa	04087120	43°02'44"	-87°59'59"
Milwaukee River at Milwaukee, WI	04087000	43°06'00"	-87°54'32"
