# Supplementary material for: High levels of sewage contamination released from urban areas after storm events: A quantitative survey with sewage specific bacterial indicators
Source: PLoS Med. 2018 Jul 24;15(7):e1002614. doi: 10.1371/journal.pmed.1002614 (PMC6057621; doi:10.1371/journal.pmed.1002614)
Supplement: S3 Table — HB, human Bacteroides; Lachno2, human Lachnospiraceae; qPCR, quantitative polymerase chain reaction. (PDF) [file pmed.1002614.s005.pdf]

S3 Table. qPCR assays slopes, Y intercepts, and efficiencies for Lachno2 and HB. HB, human Bacteroides; Lachno2, human Lachnospiraceae; qPCR, quantitative polymerase chain reaction

Target Name	Slope	Intercept	R^2	Efficiency (%)
Lachno2	-3.525	38.182	0.999	92.316
HB	-3.350	37.202	0.999	98.887
