# Supplementary material for: High levels of sewage contamination released from urban areas after storm events: A quantitative survey with sewage specific bacterial indicators
Source: PLoS Med. 2018 Jul 24;15(7):e1002614. doi: 10.1371/journal.pmed.1002614 (PMC6057621; doi:10.1371/journal.pmed.1002614)
Supplement: S4 Table — Loads of HB and Lachno2 for each river and the percentages of the total load (sum of three rivers) at automated sampling locations are shown. CSO, combined sewer overflow; HB, human Bacteroides; KK, Kinnickinnic; Lachno2, human Lachnospiraceae; MG, million gallons; MKE, Milwaukee; MMSD, Milwaukee Metropolitan Sewerage District; MN, Menomonee. (PDF) [file pmed.1002614.s006.pdf]

S4 Table. Volumes, in MG, and percentages of total volumes released from CSO outfalls upstream of automated sampling locations in the KK, MN, and MKE Rivers during CSO events in 2014 and 2015, as reported by the MMSD. Loads of HB and Lachno2 for each river and the percentages of the total load (sum of three rivers) at automated sampling locations are shown. CSO, combined sewer overflow; HB, human Bacteroides; KK, Kinnickinnic; Lachno2, human Lachnospiraceae; MG, million gallons; MKE, Milwaukee; MMSD, Milwaukee Metropolitan Sewerage District; MN, Menomonee

		Reported CSO volume upstream of sampling locations (MG)[a]	Percent of total CSO volume upstream of sampling locations	HB load/ Lachno2 load	Percent of total load of HB/ Lachno2[b]
2014 CSO	KK River	0.2	0.1	$2.6x10^{12}$/ $3.1x10^{12}$	0.3/ 0.1
	MN River	194.3	67.7	$7.7x10^{14}$/ $1.7x10^{15}$	75.8/ 78.3
	MKE River	92.5	32.2	$2.4x10^{14}$/ $4.7x10^{14}$	23.9/ 21.6
	Total	287		$1.0x10^{15}$/ $2.2x10^{15}$	
2015 CSO	KK River	0	0	$1.1x10^{12}$/ $2.6x10^{12}$	0.1/ 0.1
	MN River	323.4	74	$9.7x10^{14}$/ $1.9x10^{15}$	67.2/ 67.7
	MKE River	116.4	26	$4.7x10^{14}$/ $9.0x10^{14}$	32.7/ 32.1
	Total	439.8		$1.5x10^{15}$/ $2.8x10^{15}$	

[a] CSO volumes from outfalls downstream from sampling locations were subtracted from total CSO volumes to estimate CSO discharge upstream from monitoring stations.

[b] The load from each river was summed and values shown represent the % of this total that was measured in each river.
