# Supplementary material for: High levels of sewage contamination released from urban areas after storm events: A quantitative survey with sewage specific bacterial indicators
Source: PLoS Med. 2018 Jul 24;15(7):e1002614. doi: 10.1371/journal.pmed.1002614 (PMC6057621; doi:10.1371/journal.pmed.1002614)
Supplement: S1 Fig — Red dotted lines represent the ambient water quality standards for geometric means for E. coli (126 CFU/100 mL), enterococci (35 CFU/100 mL), and fecal coliforms (200 CFU/100 mL). CFU, colony-forming unit; KK, Kinnickinnic; MKE, Milwaukee; mL, milliliters; MN, Menomonee. (PDF) [file pmed.1002614.s010.pdf]

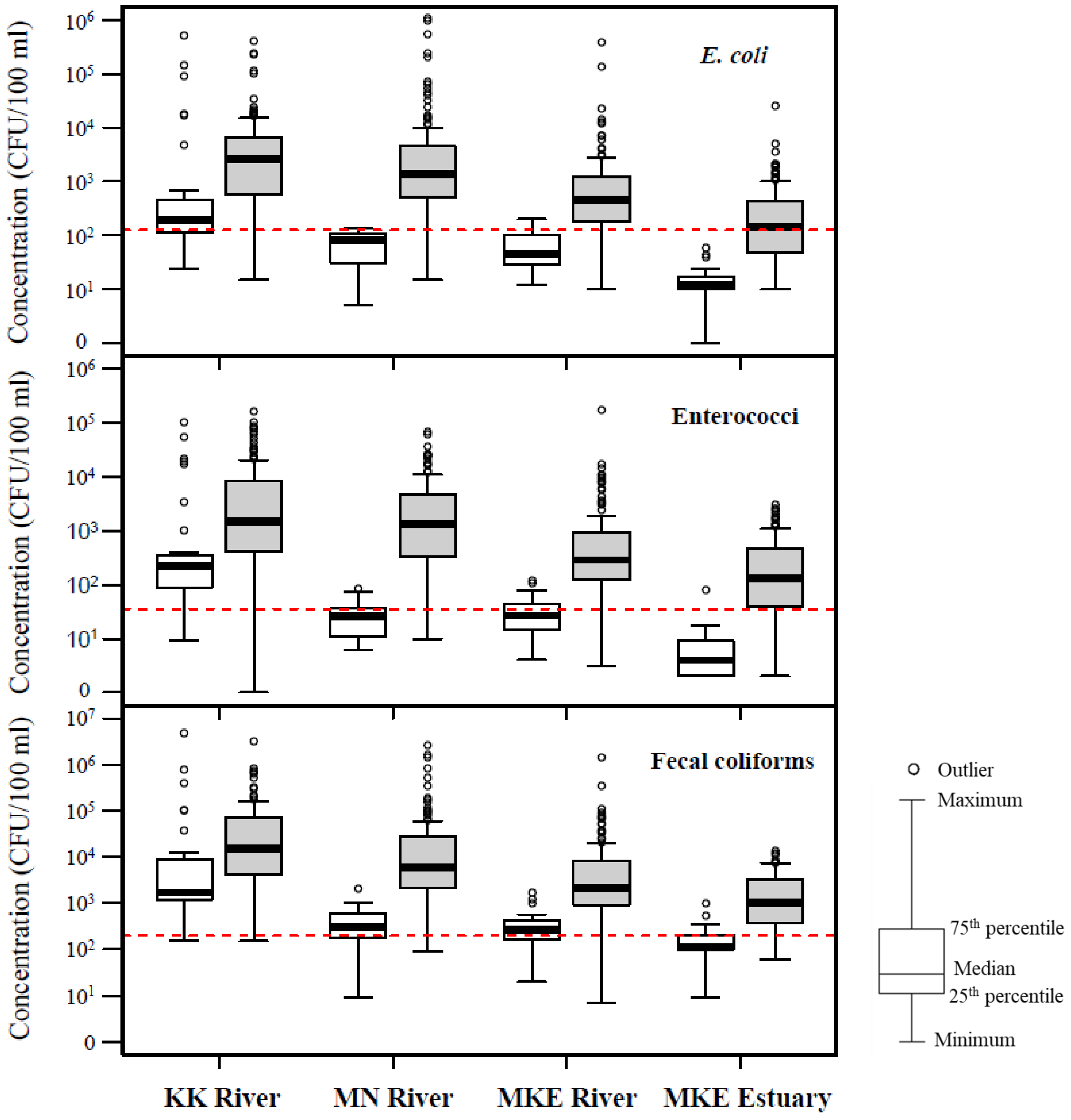

S1 Fig. Concentrations of standard fecal indicator bacteria, E. coli, enterococci, and fecal coliforms, measured in the KK, MN, and MKE Rivers, as well as the Milwaukee estuary during low-flow periods (white plots) and rain events (gray plots) in Milwaukee, Wisconsin in 2014 and 2015. Red dotted lines represent the ambient water quality standards for geometric means for E. coli (126 CFU/100 mL), enterococci (35 CFU/100 mL), and fecal coliforms (200 CFU/100 mL). CFU, colony-forming unit; KK, Kinnickinnic; MKE, Milwaukee; mL, milliliters; MN, Menomonee
