# Supplementary material for: High levels of sewage contamination released from urban areas after storm events: A quantitative survey with sewage specific bacterial indicators
Source: PLoS Med. 2018 Jul 24;15(7):e1002614. doi: 10.1371/journal.pmed.1002614 (PMC6057621; doi:10.1371/journal.pmed.1002614)
Supplement: S3 Fig — Streamflow (upper panel) and corresponding HB, Lachno2, E. coli, and enterococci indicator concentrations (lower panel) measured during rain event and low-flow periods in the KK River in Milwaukee, Wisconsin in 2014 and 2015. Each letter (A–N) represents a different sampling period. Vertical black dashed lines represent the beginning and ending dates and times that were defined for each event or low-flow period. HB, human Bacteroides; KK, Kinnickinnic; Lachno2, human Lachnospiraceae. (PDF) [file pmed.1002614.s012.pdf]

S3 Fig. Streamflow (upper panel) and corresponding HB, Lachno2, E. coli, and enterococci indicator concentrations (lower panel) measured during rain event and low-flow periods in the KK River in Milwaukee, Wisconsin in 2014 and 2015. Each letter (A–N) represents a different sampling period. Vertical black dashed lines represent the beginning and ending dates and times that were defined for each event or low-flow period. HB, human Bacteroides; KK, Kinnickinnic; Lachno2, human Lachnospiraceae

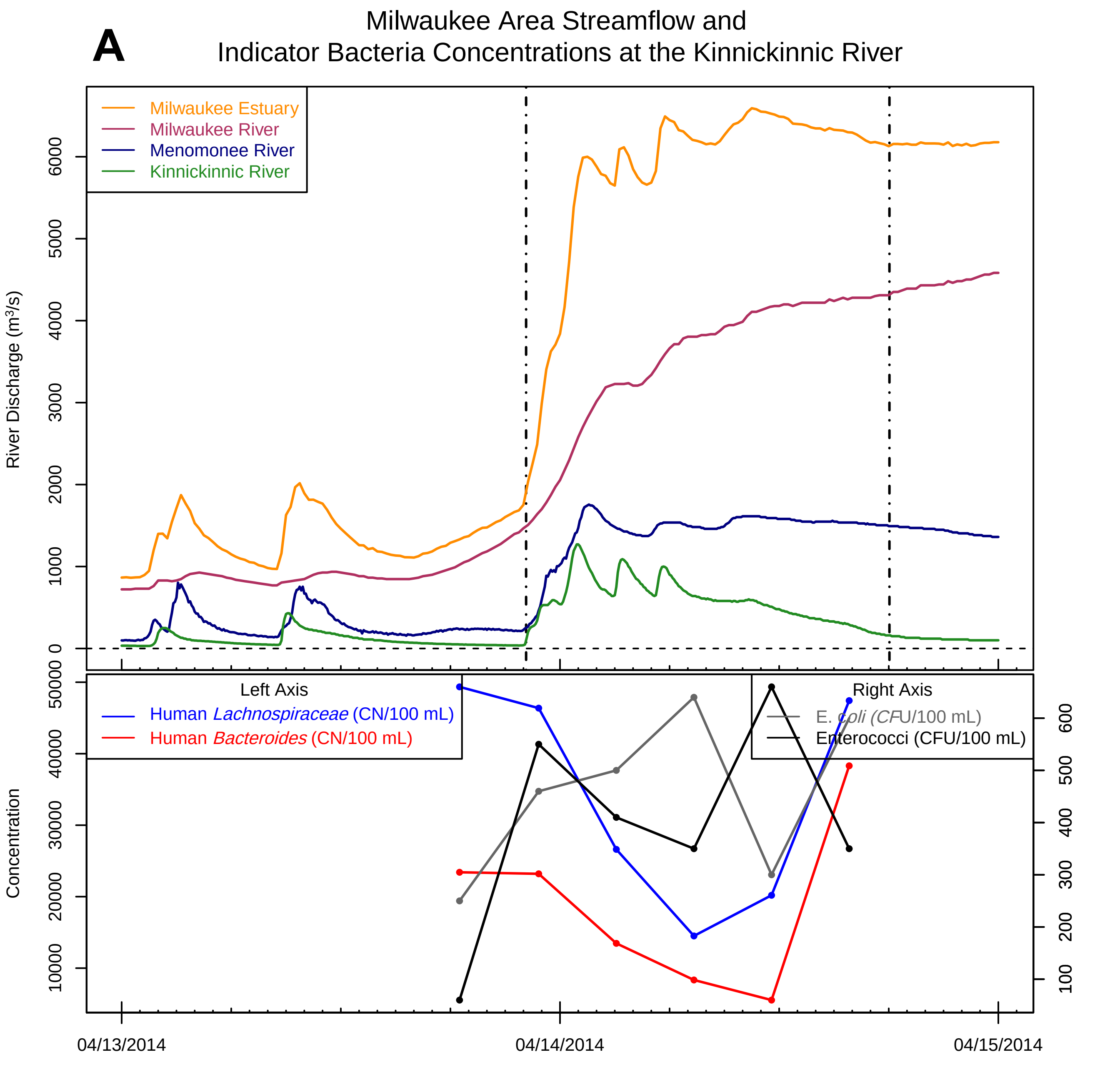

A
Milwaukee Area Streamflow and
Indicator Bacteria Concentrations at the Kinnickinnic River
Milwaukee Estuary
Milwaukee River
Menomonee River
Kinnickinnic River
River Discharge (m³/s)
0
1000
2000
3000
4000
5000
6000
Left Axis
Human Lachnospiraceae (CN/100 mL)
Human Bacteroides (CN/100 mL)
Right Axis
E. coli (CFU/100 mL)
Enterococci (CFU/100 mL)
Concentration
10000
20000
30000
40000
50000
100
200
300
400
500
600
04/13/2014
04/14/2014
04/15/2014

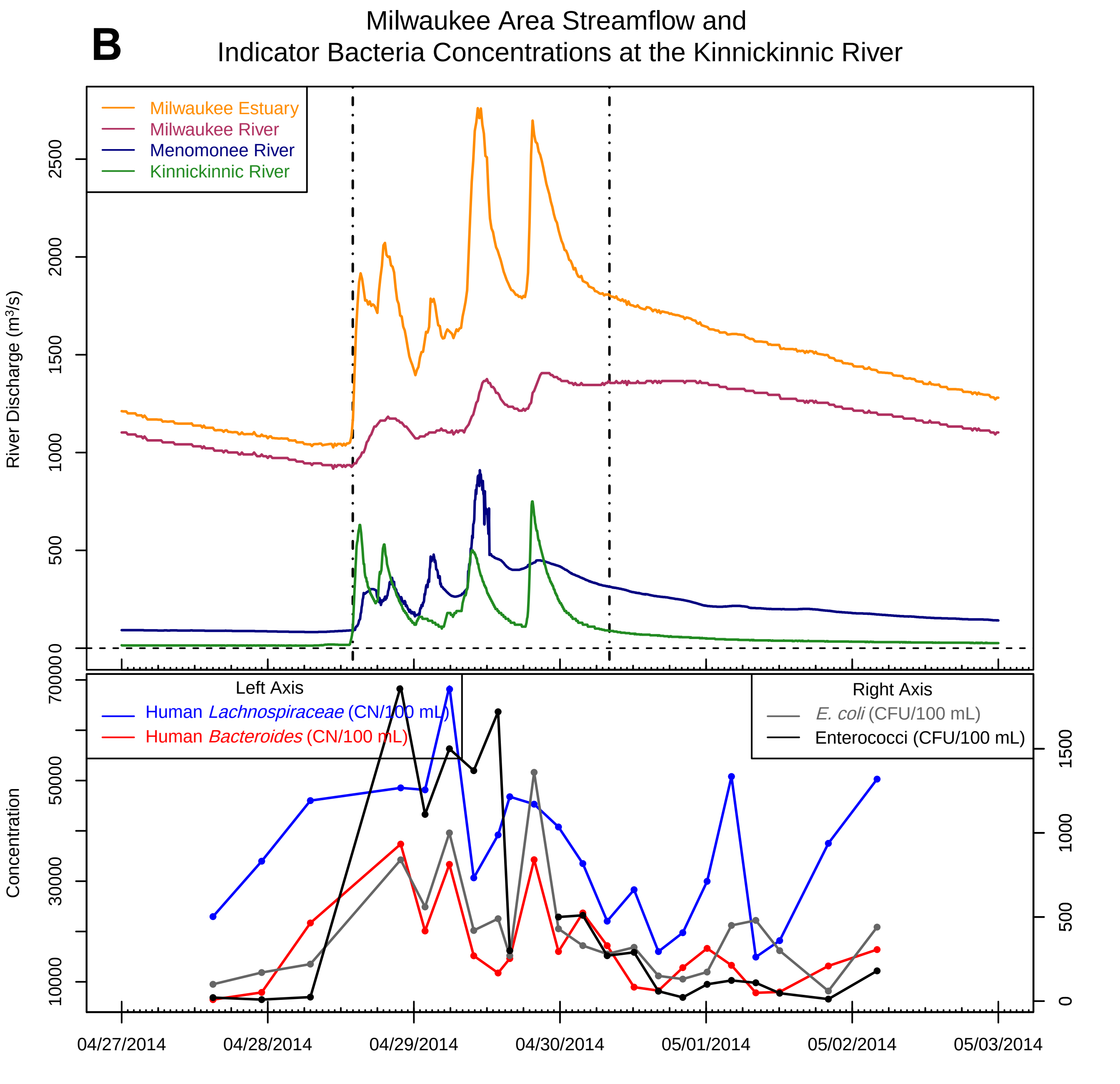
B
Milwaukee Area Streamflow and
Indicator Bacteria Concentrations at the Kinnickinnic River
Milwaukee Estuary
Milwaukee River
Menomonee River
Kinnickinnic River
River Discharge (m³/s)
0
500
1000
1500
2000
2500
Left Axis
Human Lachnospiraceae (CN/100 mL)
Human Bacteroides (CN/100 mL)
Right Axis
E. coli (CFU/100 mL)
Enterococci (CFU/100 mL)
Concentration
10000
30000
50000
70000
0
500
1000
1500
04/27/2014
04/28/2014
04/29/2014
04/30/2014
05/01/2014
05/02/2014
05/03/2014

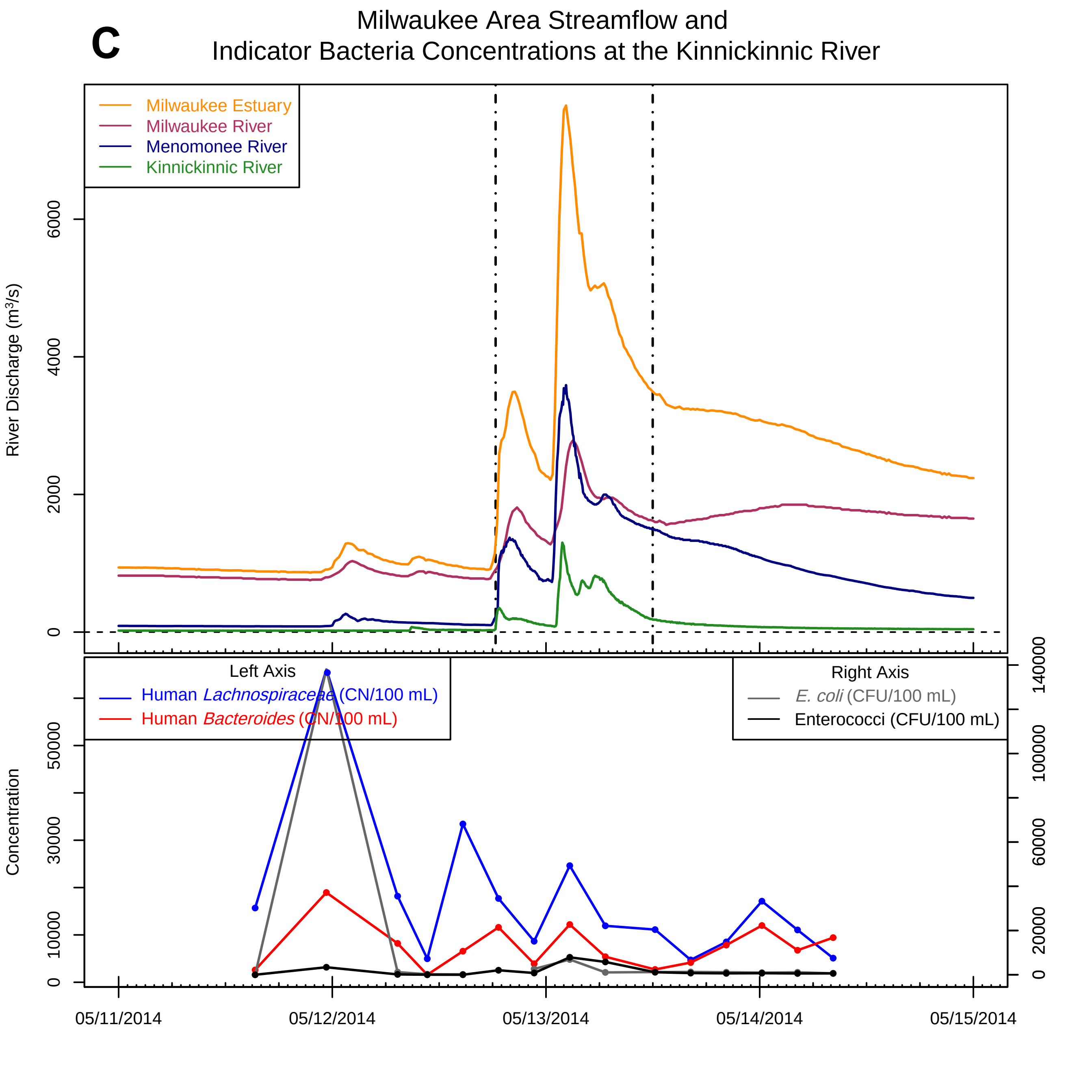

C
Milwaukee Area Streamflow and
Indicator Bacteria Concentrations at the Kinnickinnic River
Milwaukee Estuary
Milwaukee River
Menomonee River
Kinnickinnic River
River Discharge (m³/s)
0
2000
4000
6000
Left Axis
Human Lachnospiraceae (CN/100 mL)
Human Bacteroides (CN/100 mL)
Right Axis
E. coli (CFU/100 mL)
Enterococci (CFU/100 mL)
Concentration
0
10000
30000
50000
0
20000
60000
100000
140000
05/11/2014
05/12/2014
05/13/2014
05/14/2014
05/15/2014

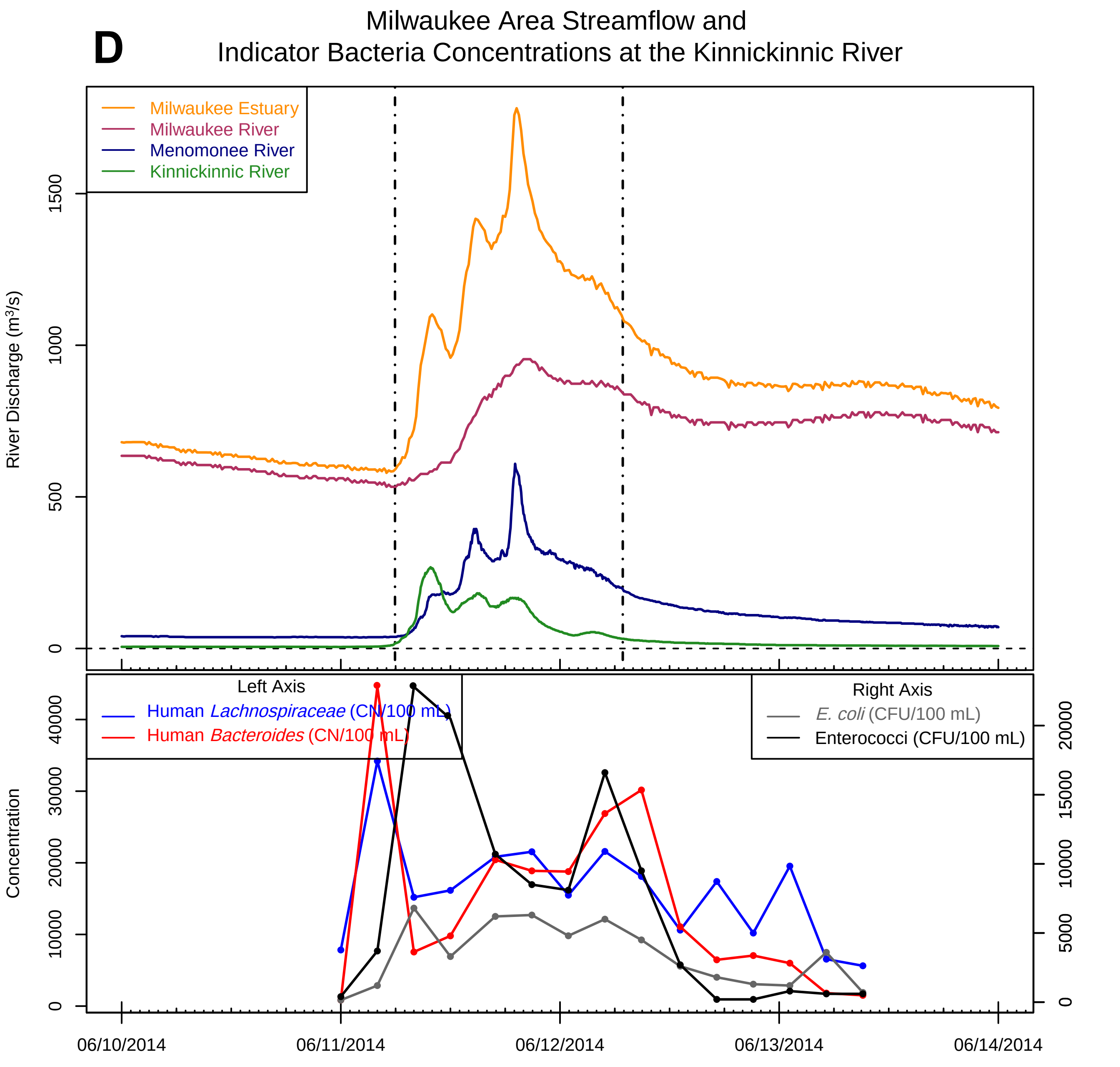

D
Milwaukee Area Streamflow and
Indicator Bacteria Concentrations at the Kinnickinnic River
Milwaukee Estuary
Milwaukee River
Menomonee River
Kinnickinnic River
River Discharge (m³/s)
0
500
1000
1500
Left Axis
Human Lachnospiraceae (CN/100 mL)
Human Bacteroides (CN/100 mL)
Right Axis
E. coli (CFU/100 mL)
Enterococci (CFU/100 mL)
Concentration
0
10000
20000
30000
40000
0
5000
10000
15000
20000
06/10/2014
06/11/2014
06/12/2014
06/13/2014
06/14/2014

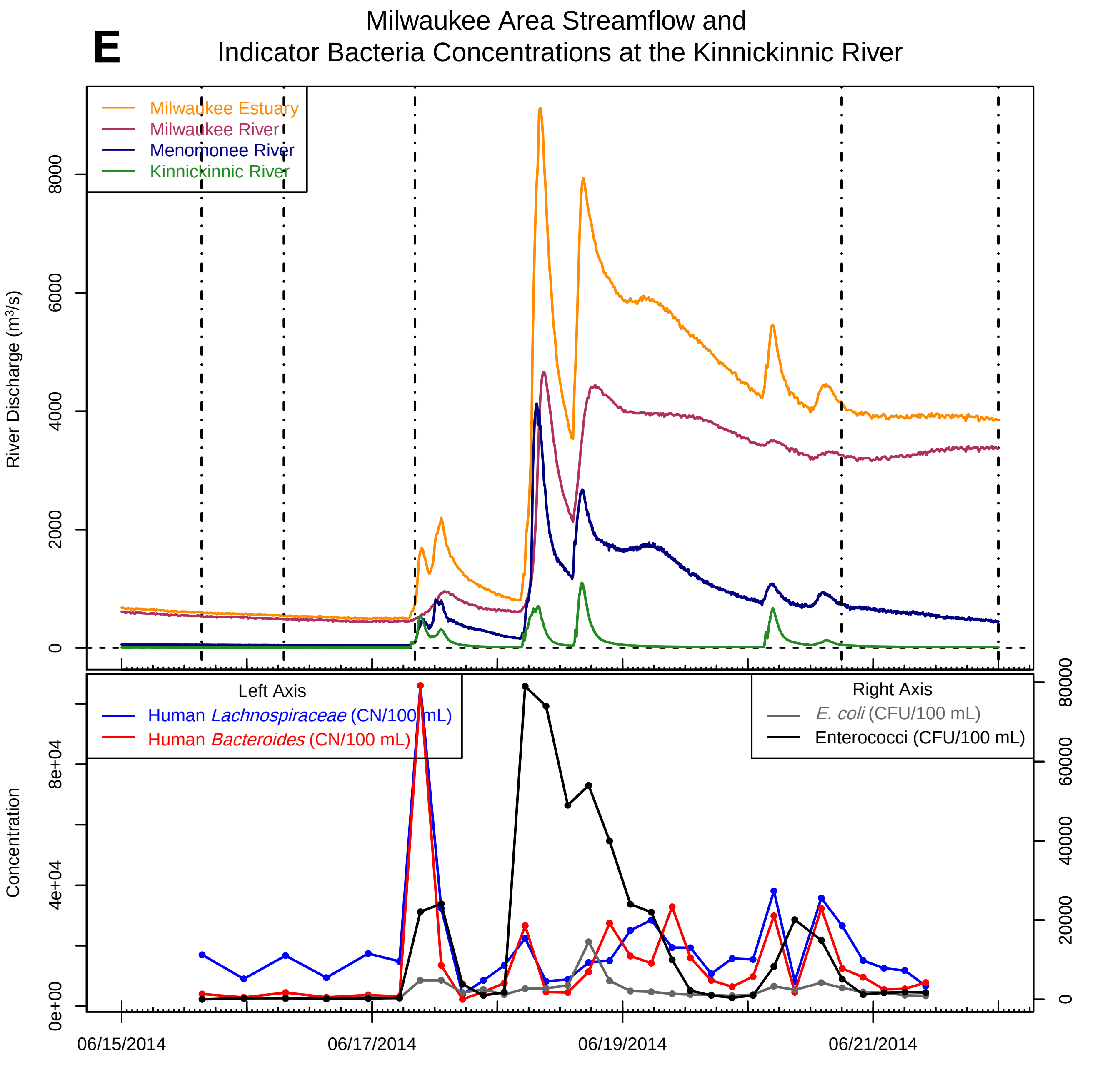

E
Milwaukee Area Streamflow and
Indicator Bacteria Concentrations at the Kinnickinnic River
Milwaukee Estuary
Milwaukee River
Menomonee River
Kinnickinnic River
River Discharge (m³/s)
0
2000
4000
6000
8000
Left Axis
Human Lachnospiraceae (CN/100 mL)
Human Bacteroides (CN/100 mL)
Right Axis
E. coli (CFU/100 mL)
Enterococci (CFU/100 mL)
Concentration
0e+00
4e+04
8e+04
0
20000
40000
60000
80000
06/15/2014
06/17/2014
06/19/2014
06/21/2014

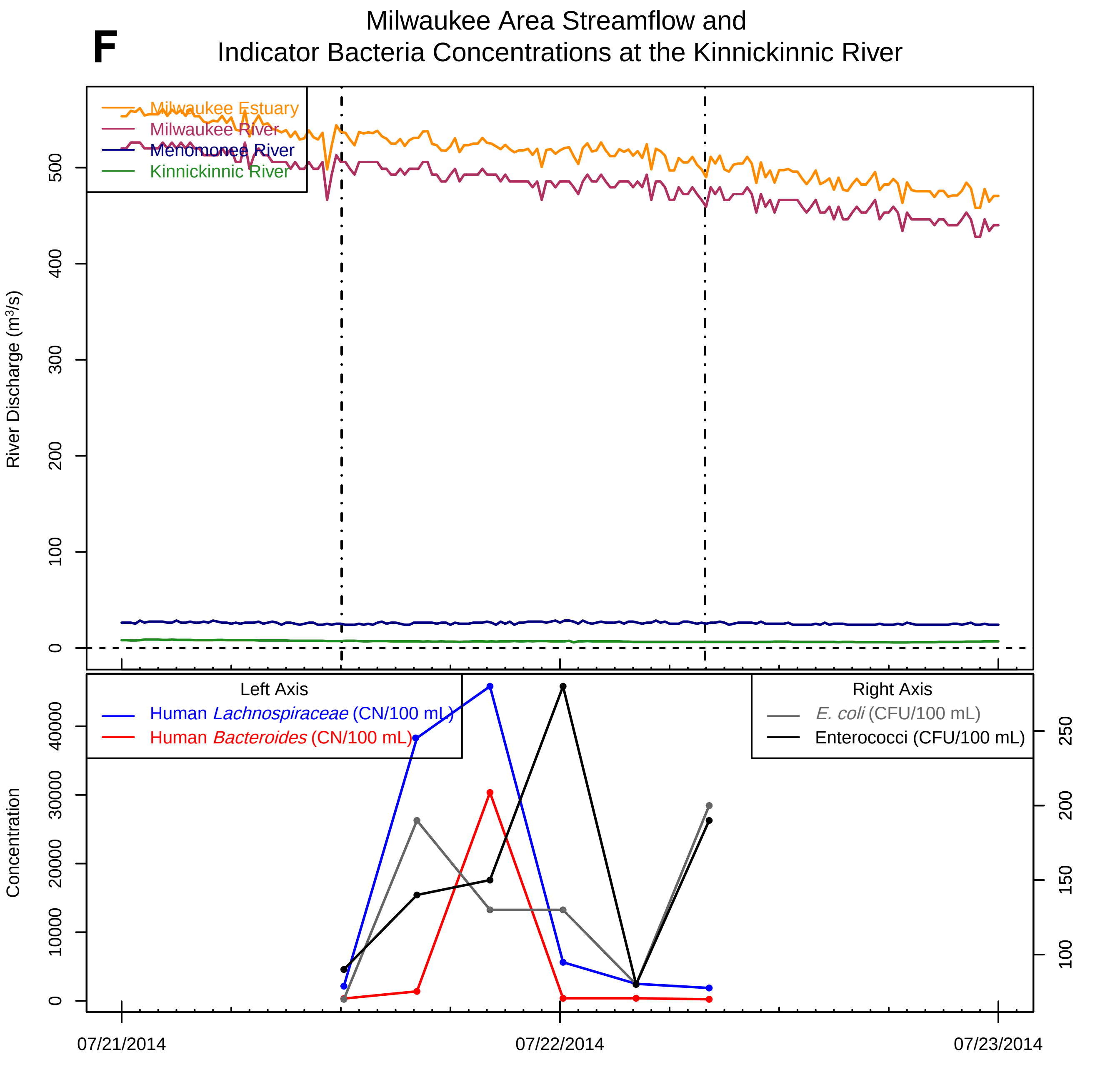
F
Milwaukee Area Streamflow and
Indicator Bacteria Concentrations at the Kinnickinnic River
Milwaukee Estuary
Milwaukee River
Menomonee River
Kinnickinnic River
River Discharge (m³/s)
0
100
200
300
400
500
Left Axis
Human Lachnospiraceae (CN/100 mL)
Human Bacteroides (CN/100 mL)
Right Axis
E. coli (CFU/100 mL)
Enterococci (CFU/100 mL)
Concentration
0
10000
20000
30000
40000
100
150
200
250
07/21/2014
07/22/2014
07/23/2014

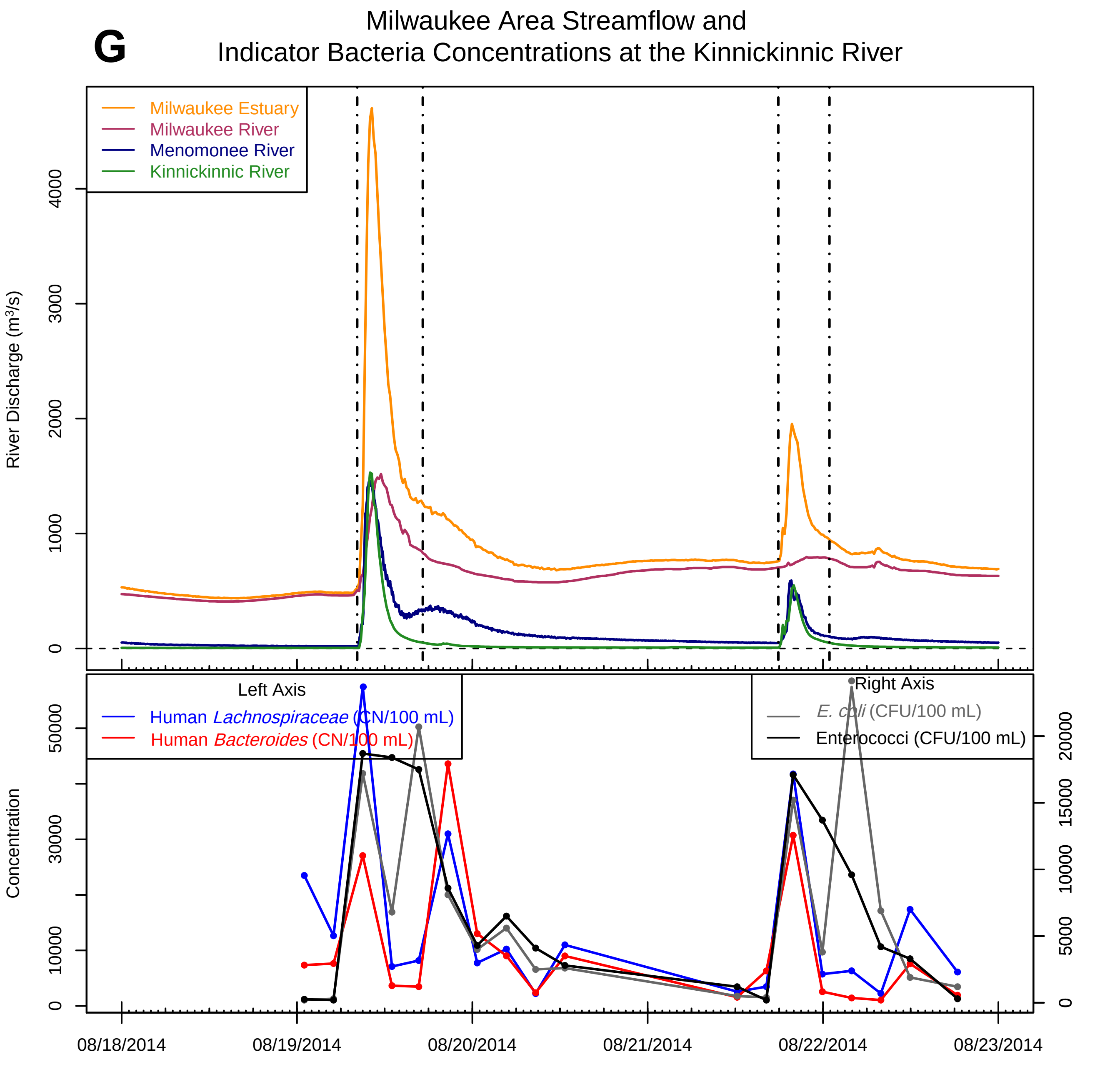

G
Milwaukee Area Streamflow and
Indicator Bacteria Concentrations at the Kinnickinnic River
Milwaukee Estuary
Milwaukee River
Menomonee River
Kinnickinnic River
River Discharge (m³/s)
0
1000
2000
3000
4000
Left Axis
Human *Lachnospiraceae* (CN/100 mL)
Human *Bacteroides* (CN/100 mL)
Right Axis
E. coli (CFU/100 mL)
Enterococci (CFU/100 mL)
Concentration
0
10000
30000
50000
5000
15000
20000
08/18/2014
08/19/2014
08/20/2014
08/21/2014
08/22/2014
08/23/2014

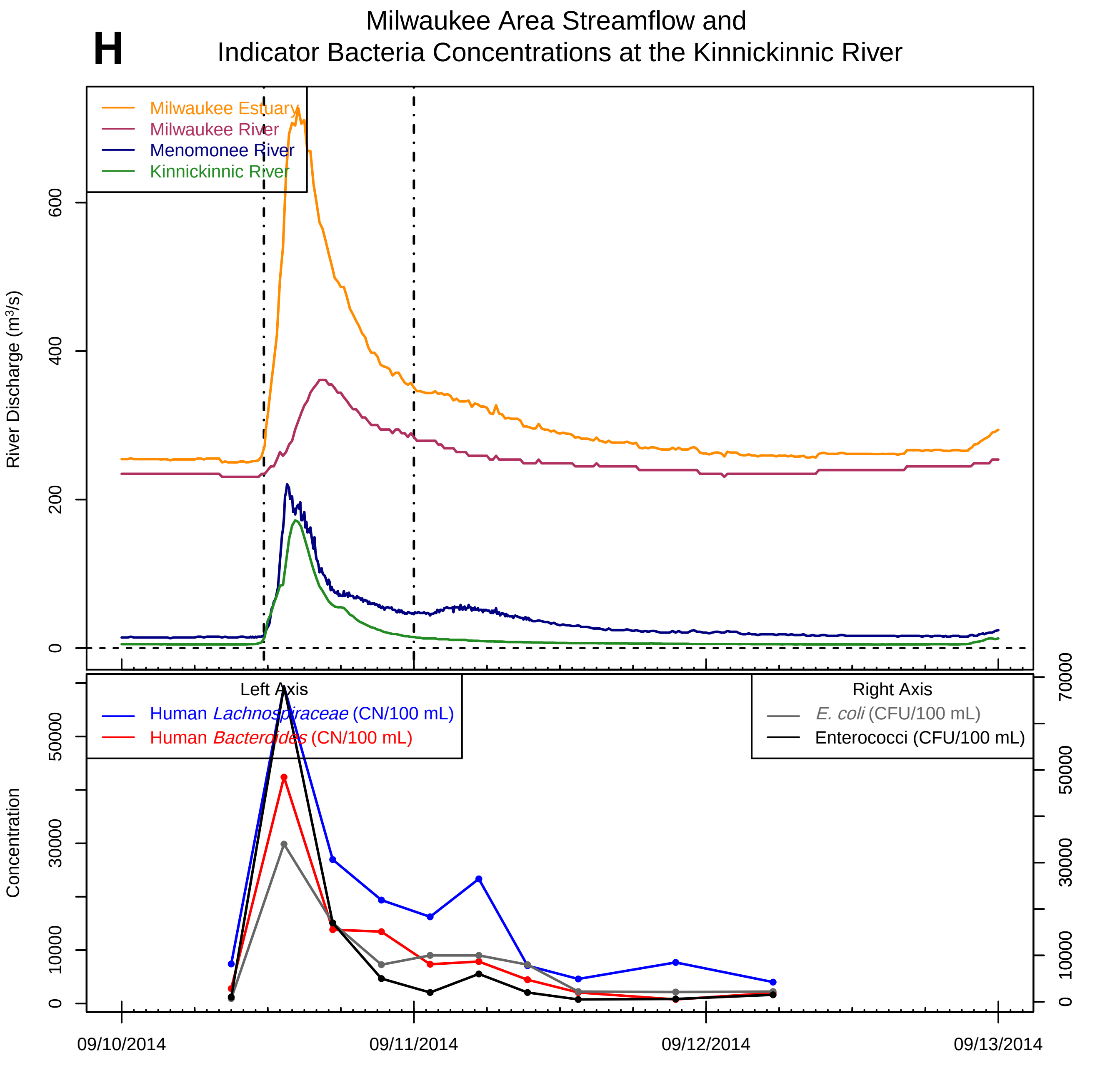
H
Milwaukee Area Streamflow and
Indicator Bacteria Concentrations at the Kinnickinnic River
Milwaukee Estuary
Milwaukee River
Menomonee River
Kinnickinnic River
River Discharge (m³/s)
600
400
200
0
Left Axis
Human *Lachnospiraceae* (CN/100 mL)
Human *Bacteroides* (CN/100 mL)
Right Axis
E. coli (CFU/100 mL)
Enterococci (CFU/100 mL)
Concentration
50000
30000
10000
0
70000
50000
30000
10000
0
09/10/2014
09/11/2014
09/12/2014
09/13/2014

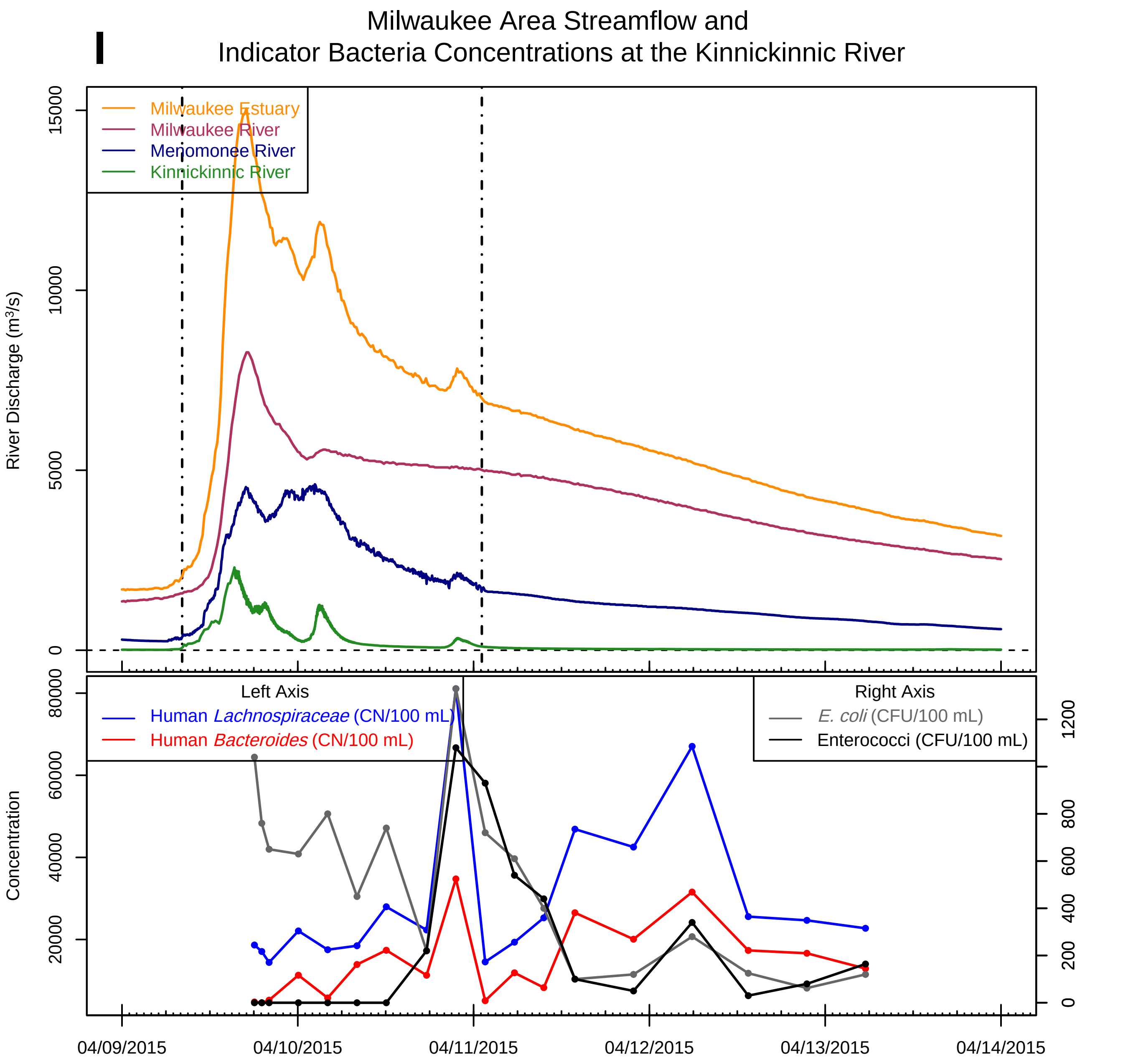

Milwaukee Area Streamflow and
Indicator Bacteria Concentrations at the Kinnickinnic River
Milwaukee Estuary
Milwaukee River
Menomonee River
Kinnickinnic River
River Discharge (m³/s)
0
5000
10000
15000
Left Axis
Human Lachnospiraceae (CN/100 mL)
Human Bacteroides (CN/100 mL)
Right Axis
E. coli (CFU/100 mL)
Enterococci (CFU/100 mL)
Concentration
20000
40000
60000
80000
0
200
400
600
800
1200
04/09/2015
04/10/2015
04/11/2015
04/12/2015
04/13/2015
04/14/2015

J

Milwaukee Area Streamflow and
Indicator Bacteria Concentrations at the Kinnickinnic River

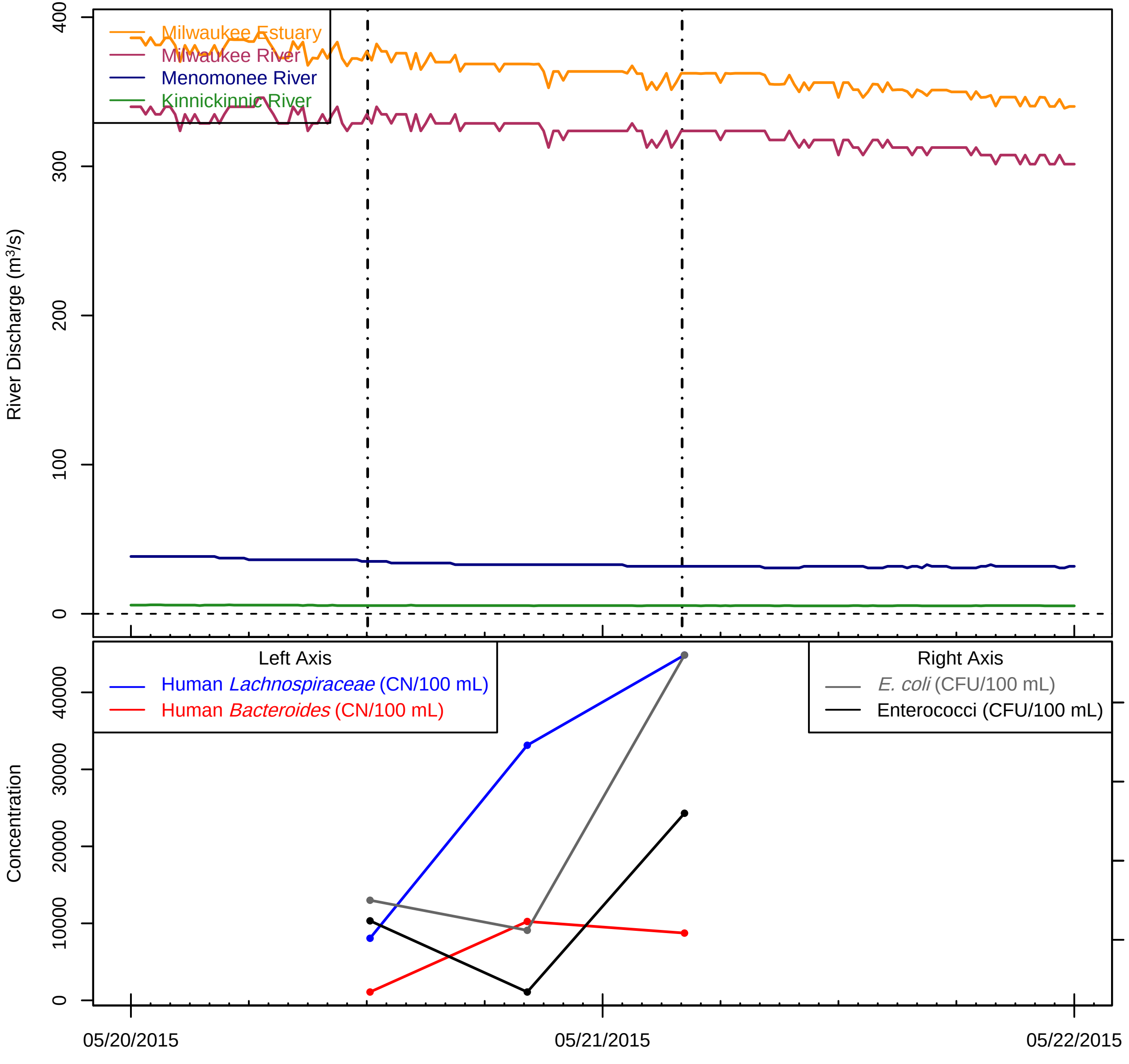

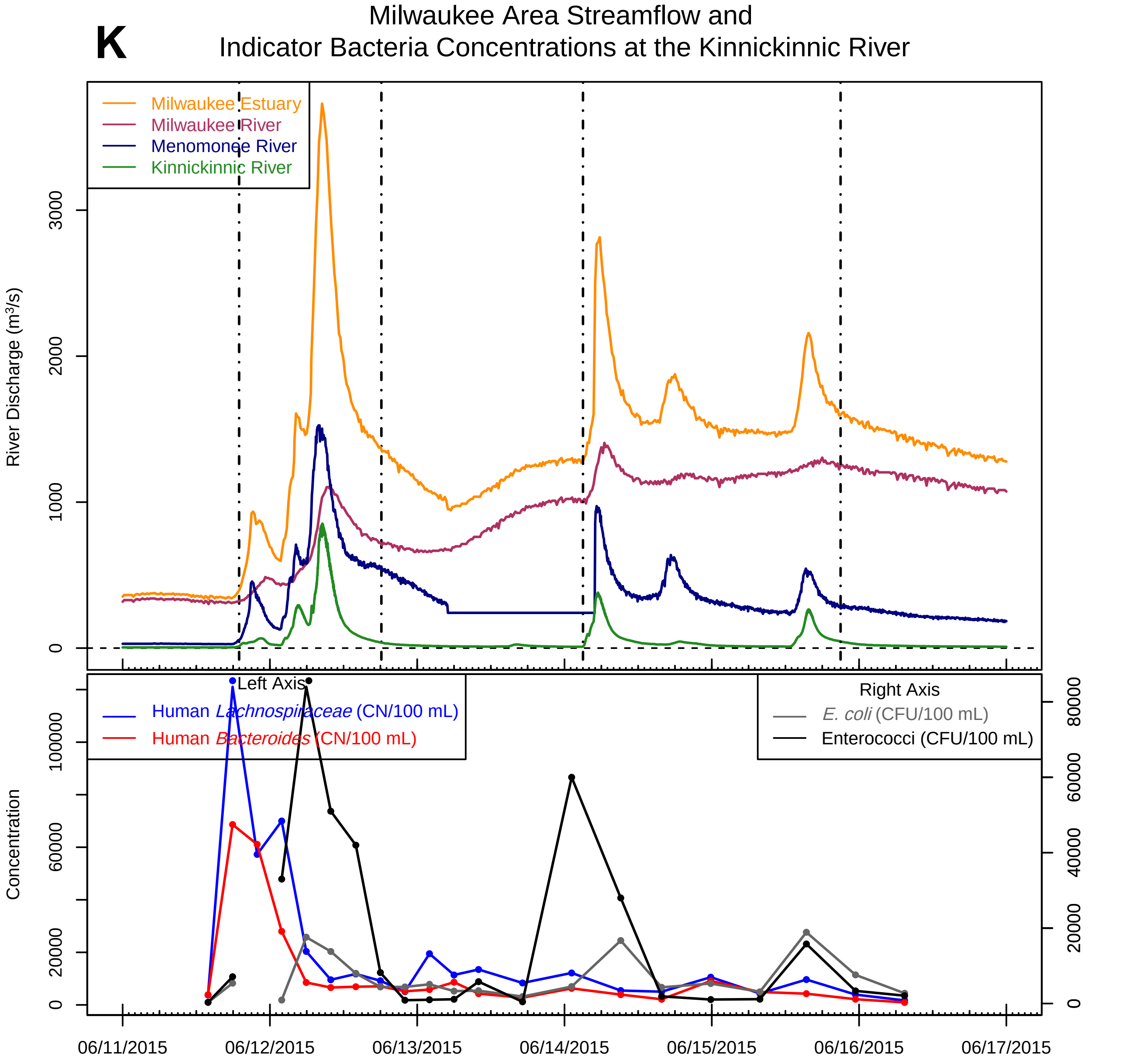

K
Milwaukee Area Streamflow and
Indicator Bacteria Concentrations at the Kinnickinnic River
Milwaukee Estuary
Milwaukee River
Menomonee River
Kinnickinnic River
River Discharge (m³/s)
0
1000
2000
3000
Left Axis
Human Lachnospiraceae (CN/100 mL)
Human Bacteroides (CN/100 mL)
Right Axis
E. coli (CFU/100 mL)
Enterococci (CFU/100 mL)
Concentration
0
20000
60000
100000
80000
40000
06/11/2015
06/12/2015
06/13/2015
06/14/2015
06/15/2015
06/16/2015
06/17/2015

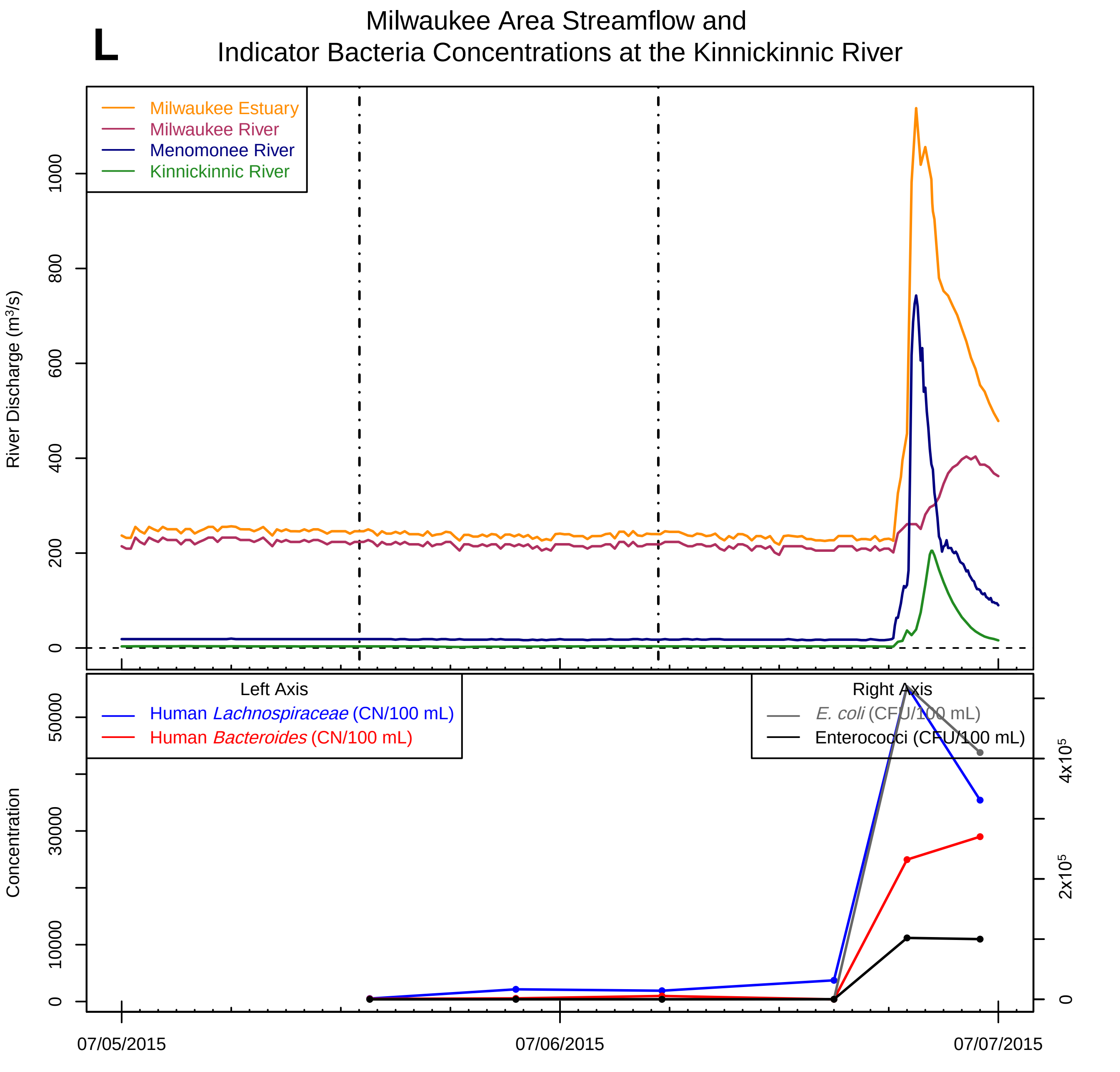
L
Milwaukee Area Streamflow and
Indicator Bacteria Concentrations at the Kinnickinnic River
Milwaukee Estuary
Milwaukee River
Menomonee River
Kinnickinnic River
River Discharge (m³/s)
0
200
400
600
800
1000
Left Axis
Human Lachnospiraceae (CN/100 mL)
Human Bacteroides (CN/100 mL)
Right Axis
E. coli (CFU/100 mL)
Enterococci (CFU/100 mL)
Concentration
0
10000
30000
50000
0
2x10⁵
4x10⁵
07/05/2015
07/06/2015
07/07/2015

M

Milwaukee Area Streamflow and
Indicator Bacteria Concentrations at the Kinnickinnic River

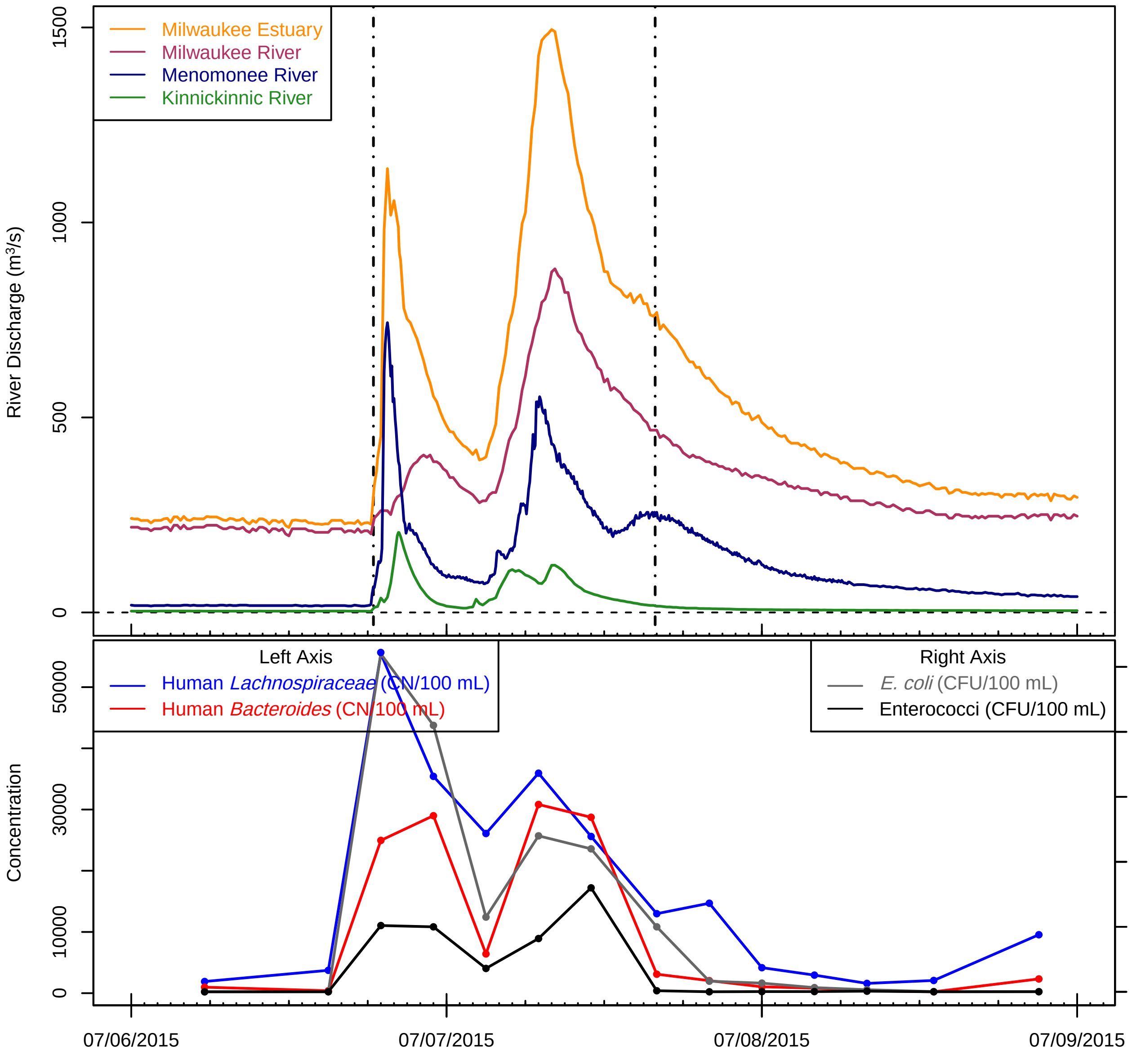

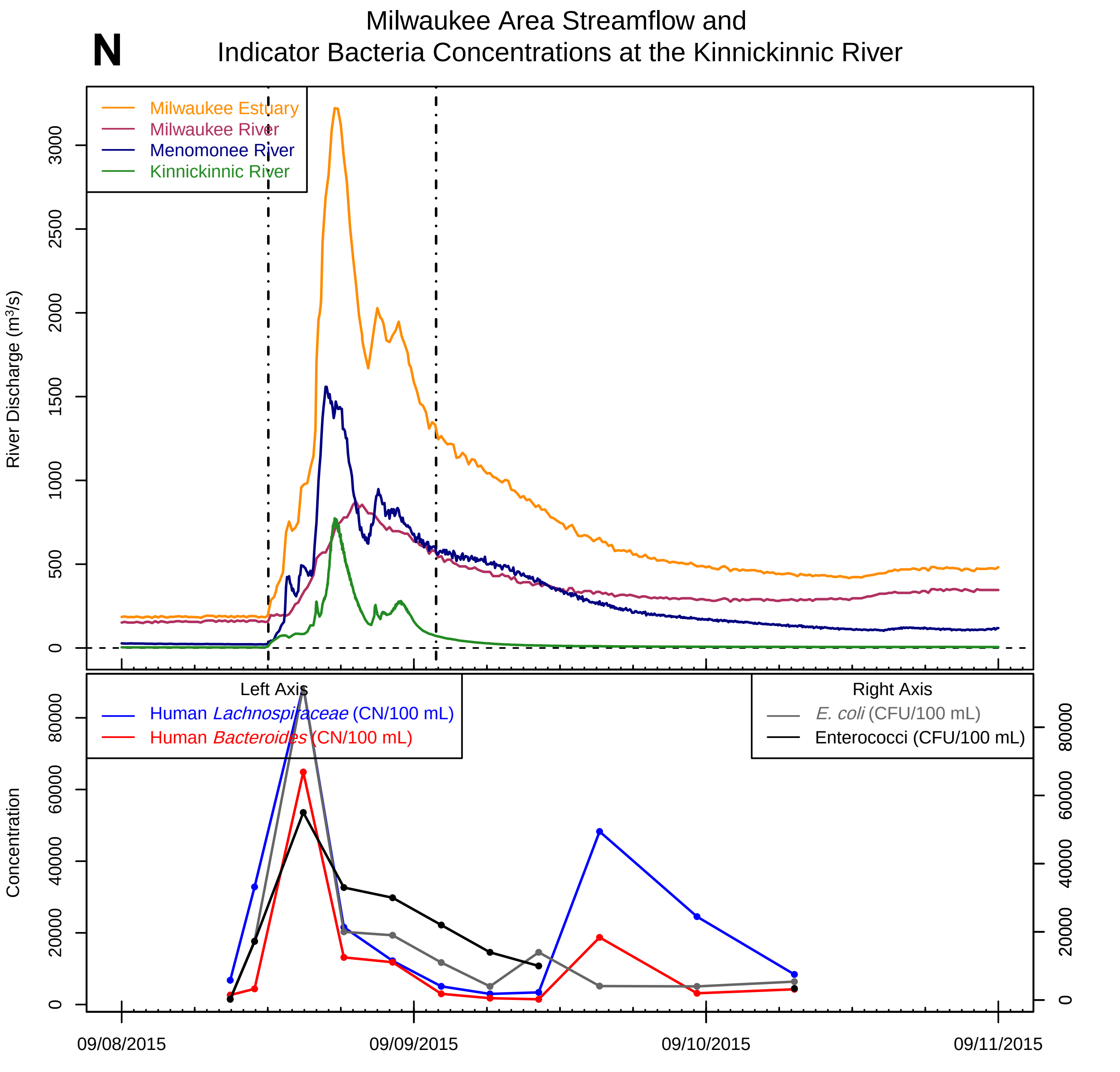
N
Milwaukee Area Streamflow and
Indicator Bacteria Concentrations at the Kinnickinnic River
Milwaukee Estuary
Milwaukee River
Menomonee River
Kinnickinnic River
River Discharge (m³/s)
0
500
1000
1500
2000
2500
3000
Left Axis
Human Lachnospiraceae (CN/100 mL)
Human Bacteroides (CN/100 mL)
Right Axis
E. coli (CFU/100 mL)
Enterococci (CFU/100 mL)
Concentration
0
20000
40000
60000
80000
09/08/2015
09/09/2015
09/10/2015
09/11/2015
